# The relationship between digital literacy and employment quality: Evidence from a Chinese Household Survey

**DOI:** 10.1371/journal.pone.0358194

**Published:** 2026-09-22

**Authors:** Xiaoyun Wu, Danting Cao, Beihe Wu

**Affiliations:** 1 College of Economics and Management, Yuzhang Normal University, Nanchang, China; 2 Modernization Development Research Center of Nanchang Metropolitan Area, Yuzhang Normal University, Nanchang, China; 3 College of Economics and Management, Jiangxi Agricultural University, Nanchang, China; Bangladesh Army University of Science and Technology, BANGLADESH

## Abstract

In the digital era, digital literacy has emerged as a critical determinant of employment quality in the labor market. Using nationally representative data from the 2022 China Family Panel Studies (CFPS), this study constructs multidimensional indices of digital literacy and employment quality. Ordinary least squares (OLS) regressions, robustness checks, and moderation models are employed to examine the relationships between these variables and the underlying mechanisms. The findings suggest that digital literacy is significantly associated with employment quality. This relationship is positively moderated by return expectations and negatively moderated by human capital, indicating a stronger association among low-skilled workers with high return expectations. Regional heterogeneity reveals the most significant associations in western China and urban agglomerations—attributable to higher marginal returns on digital skills in underdeveloped regions and denser digital economies in clustered zones. Digital literacy emerges as a pivotal correlate of employment quality, particularly for vulnerable groups and lagging regions. Policy interventions should prioritize (1) inclusive, tiered digital training programs targeting low-skilled workers; (2) regionally differentiated strategies; and (3) leveraging urban agglomerations as hubs for cross-regional resource sharing. These approaches can help narrow digital divides and advance sustainable, equitable, and high-quality employment in the digital era.

## Introduction

As a crucial indicator for measuring the health of the labor market in a country or region, employment quality is not only intricately linked to the well-being of individual workers but also plays a significant role in maintaining social stability and promoting sustainable economic development. Amid the global digital transformation, China’s economy is transitioning from a phase of high-speed growth to one of high-quality development. Concomitant with this transition, China’s labor market has shifted from quantitative expansion to qualitative enhancement, placing greater emphasis on the sustainability, fairness, and stability of employment [1]. The 2025 Government Work Report of China clearly outlines the new-era objective of promoting high-quality and comprehensive employment, identifying it as a strategic priority for employment-related initiatives. The report emphasizes that achieving this objective requires the implementation of more robust and coordinated measures to ensure the stability of the labor market and the sustained expansion of employment opportunities. Furthermore, with the rapid advancement of information technology and the deepening of digital transformation, China's labor market is experiencing unprecedented disruption and restructuring. Many traditional roles that rely on repetitive tasks are being replaced by automated systems, while the demand for advanced skills in emerging digital occupations continues to rise [2,3].

Against this backdrop, the relationship between digital literacy and employment quality has emerged as a central issue. This study conceptualizes “digital literacy” as workers’ comprehensive capacity to acquire, process, and apply digital information and technology in digital environments—encompassing both objective operational skills and subjective cognitive dispositions [4]. Meanwhile, drawing on the International Labour Organization’s (ILO) “Decent Work” framework, this study defines “employment quality” as the comprehensive manifestation of both objective economic benefits and subjective value realization experienced by workers during employment, and assesses it across multiple dimensions—including the objective dimension (e.g., wages, employment security, and career development opportunities) and the subjective dimension (e.g., job satisfaction) [5].

Digital literacy—the ability of individuals to effectively navigate, evaluate, and use digital technologies to enhance work efficiency and quality of life—has become an essential core competency in the labor market and is increasingly recognized as a critical determinant of employment quality [6]. However, the digital divide, which produces disparities in digital literacy across populations, not only restricts access to employment opportunities but also significantly affects job stability, income levels, and long-term career development [7]. Therefore, examining how digital literacy influences employment quality and identifying the underlying mechanisms are critically important for improving labor market efficiency and promoting sustainable economic growth.

Regarding the definition of digital literacy, the academic community initially emphasized its importance as a fundamental life skill, defining it as an individual’s ability to use computers and the Internet for information retrieval, communication, and processing [8]. However, as digital transformation progresses and deepens, the concept of digital literacy has expanded and evolved. It now encompasses not only basic technical competencies—such as computer and network operations—but also higher-order skills, including the identification, evaluation, synthesis, and creative application of information, as well as the ability to solve problems effectively using digital tools [4]. Consequently, the understanding of digital literacy has moved beyond the purely technical level to incorporate a broader set of knowledge, skills, and attitudes. Scholars emphasize the essential, integrated competencies individuals must cultivate in the digital age, with key components including digital skills and digital awareness [9]. The development of these dimensions of digital literacy plays a crucial role in enhancing individuals’ employability, job competitiveness, and career resilience.

To ground this study in established theoretical discourse, it is essential to acknowledge foundational digital literacy frameworks. Digital divide theory conceptualizes digital inequality across multiple dimensions—from access and skills to usage patterns and tangible outcomes—providing a structural lens for analyzing how literacy disparities translate into labor market inequities [10]. Complementing this, UNESCO’s Global Digital Literacy Framework defines digital literacy as a life skill encompassing seven core competency areas—including device and software operation, information and data literacy, communication and collaboration, and digital safety—emphasizing its role in personal development and social participation [11]. At the policy level, the European Union’s DigComp framework has been instrumental in operationalizing digital competence for citizens, outlining five key areas—information and data literacy, communication and collaboration, digital content creation, safety, and problem solving—that align closely with the demands of evolving labor markets. These theoretical and policy-oriented frameworks collectively affirm that digital literacy is not merely a technical skill but a multidimensional capability critical for socio-economic integration and opportunity.

A substantial body of research has demonstrated that digital literacy significantly contributes to facilitating access to employment opportunities [12], enhancing employability [13], fostering entrepreneurship [14], and promoting innovative work behaviors [15]. However, when it comes to the more holistic and policy-critical outcome of “high-quality employment,” the extant literature exhibits discernible gaps at both methodological and conceptual levels, which this study aims to address.

Methodologically, three main shortcomings are evident. First, there is a prevalent reliance on oversimplified measurement. Employment outcomes are often reduced to binary employment status or singular metrics like income, failing to capture the multidimensional nature of employment quality that encompasses both objective conditions (e.g., security, prospects) and subjective perceptions (e.g., satisfaction) [16]. Similarly, digital literacy is frequently proxied by basic indicators such as internet access or usage frequency [17], neglecting its composite character involving both operational skills and cognitive dispositions. Second, there is a lack of systematic investigation into boundary conditions. While a direct positive link is often assumed, the moderating mechanisms (e.g., how individual expectations or existing human capital alter the strength of this link) remain underexplored and poorly tested. Third, studies often overlook substantial heterogeneity across regions and developmental contexts, treating the digital literacy-employment quality relationship as uniform despite plausible variations due to differing economic structures and digital infrastructure.

Conceptually, the literature is limited by narrow framing. On one hand, the conceptualization of digital literacy has not kept pace with its evolving complexity, often remaining confined to technical proficiency without integrating the awareness and evaluative capacities crucial for effective application in the workplace [9]. On the other hand, the concept of employment quality is seldom grounded in integrated frameworks like the ILO’s Decent Work agenda [5], which would allow for a balanced assessment of both economic and psychosocial dimensions.

Therefore, this study seeks to bridge these gaps by: (1) conceptually advancing multidimensional frameworks for both digital literacy (encompassing objective ability and subjective awareness) and employment quality (aligned with the Decent Work dimensions), and (2) methodologically constructing composite indices based on these frameworks; empirically testing the direct relationship; rigorously examining the moderating roles of return expectations and human capital; and conducting detailed heterogeneity analyses across regions and agglomeration zones. This integrated approach not only offers theoretical refinement but also yields nuanced empirical evidence to inform targeted and effective policy interventions aimed at fostering inclusive and sustainable employment in the digital era.

Based on this, this study employs data from the 2022 China Family Panel Studies (CFPS). Through empirical analysis, it aims to achieve the following specific research objectives: (1) to construct a multi-dimensional, comprehensive evaluation index system for digital literacy and employment quality; (2) to empirically test the relationship between digital literacy and the employment quality of workers; (3) to identify the moderating roles of return expectations and human capital in this relationship; (4) to investigate the heterogeneity of these effects across regions (eastern, central, and western China) and across areas with differing levels of resource agglomeration (urban agglomerations versus non-urban agglomerations). This study aims to provide micro-level evidence and policy implications to promote higher-quality, fairer, and more sustainable labor market development amid digital transformation.

The remainder of this paper is organized as follows: Section 2 consists of the theoretical analysis and research hypotheses. Section 3 describes the methodology and data, including the research methodology, variable selection, and data sources. Section 4 presents and analyzes the main findings. Further discussion is provided in Section 5. Finally, key conclusions and policy implications are summarized in Section 6.

## Theoretical analysis and research hypothesis

### The direct promoting effect of digital literacy on employment quality

Digital literacy, as a critical indicator for evaluating workers’ employability, not only reflects their comprehensive capabilities in acquiring, processing, and applying information but is also strongly associated with their adaptability and competitiveness in the digital economy [18]. Specifically, workers with higher levels of digital literacy are better equipped to utilize digital tools efficiently, thereby improving work productivity. This enhanced capacity enables them not only to access a wider array of job opportunities but, more crucially, to obtain positions featuring higher wages, better employment security, and greater prospects for career development—core aspects of employment quality [14,19]. Furthermore, enhancing digital literacy enables workers to more effectively comprehend and adapt to the continuously evolving professional environment, thereby establishing a strong foundation for their personal career growth and advancement [20]. Moreover, digital literacy broadens workers’ access to information, mitigates information asymmetry, and reduces information acquisition costs. These factors collectively contribute to alleviating structural imbalances in the labor market and promoting an overall improvement in the quality of employment [21,22]. Based on the foregoing, this paper formulates Research Hypothesis 1.

**Hypothesis 1:** Digital literacy is significantly positively associated with labor employment quality.

### The positive moderating effect of expected returns

The expectancy theory of returns posits that an individual's investment in a particular skill or ability is driven by the anticipation of future benefits [23]. In the context of digital literacy, workers generally believe that enhancing their digital competencies can lead to improved employment opportunities, increased remuneration, and expanded career advancement prospects [24]. This anticipated return acts as a crucial motivational factor, encouraging individuals to acquire digital skills and elevate their digital proficiency proactively. When employees perceive that digital competencies significantly enhance their career trajectories and job quality, they are more likely to commit time and effort toward mastering these skills, thereby continuously improving their overall digital literacy [25]. Specifically, when employees anticipate that improving their digital literacy will result in enhanced professional status and greater economic benefits, they are more likely to actively acquire and apply digital competencies, thereby contributing to an improvement in the quality of their employment. The positive moderating effect is not limited to the individual level; it may also trigger a cascading impact across the broader labor market, thereby promoting an overall improvement in employment quality. From the perspective of sustainable labor market development, the “incentive effect” generated by high return expectations can motivate workers to continuously invest in digital human capital. This contributes to the dynamic updating of labor skills, helps address the challenges posed by digital transformation, and constitutes a crucial psychological mechanism for achieving long-term human capital accumulation and market vitality. Therefore, return expectations serve as a moderating variable between digital literacy and employment quality. Based on the foregoing, this paper formulates Research Hypothesis 2.

**Hypothesis 2:** Return expectations positively moderates the association of digital literacy on labor employment quality.

### The negative moderating effect of human capital

Human capital theory suggests that an individual’s educational attainment, professional skills, and practical experience serve as fundamental pillars for career development and employment quality [26,27]. Digital literacy is not only a key indicator of human capital accumulation, but its influence on the quality of labour employment is also significantly mediated by the individual’s existing level of human capital [15].

We propose a negative moderating effect of human capital based on three complementary mechanisms. First, diminishing marginal returns: workers with high levels of human capital—such as advanced education and extensive work experience—have already developed strong employability through formal educational investments. Their employment quality is largely determined by these established credentials, leaving relatively less room for digital literacy to contribute additional improvements. In contrast, workers with low human capital face more substantial skill deficits, and the acquisition of digital literacy can serve as a powerful “skill compensator,” generating larger marginal improvements in their employment outcomes.

Second, substitution and opportunity cost effects: highly educated workers may have already acquired a broad set of competencies that partially overlap with or substitute for digital skills (e.g., analytical reasoning, problem‑solving, and information evaluation). Moreover, they may face higher opportunity costs in terms of time and effort required to acquire new digital skills, potentially reducing their relative motivation to invest in digital literacy enhancement [28]. Workers with lower educational attainment, by contrast, may perceive digital skills as a more novel and valuable addition to their skill set, leading to greater relative gains.

Third, cognitive and behavioral inertia: individuals with high human capital may exhibit over‑reliance on their existing competencies, leading to lower engagement with and adoption of emerging digital skill sets [28]. This behavioral pattern can attenuate the potential benefits of digital literacy for this group. Conversely, low‑human‑capital workers often face disadvantages in the labour market [29], making them more receptive to new skill acquisition as a strategy for improving their employment prospects.

In summary, while digital literacy contributes positively to employment quality overall, the marginal benefit tends to be larger for individuals with lower human capital—a pattern consistent with the “inclusive growth” potential of digital upskilling policies. Based on the above analysis, this paper formulates Research Hypothesis 3.

**Hypothesis 3:** Human capital negatively moderates the association of digital literacy on labor employment quality.

**Fig 1:**
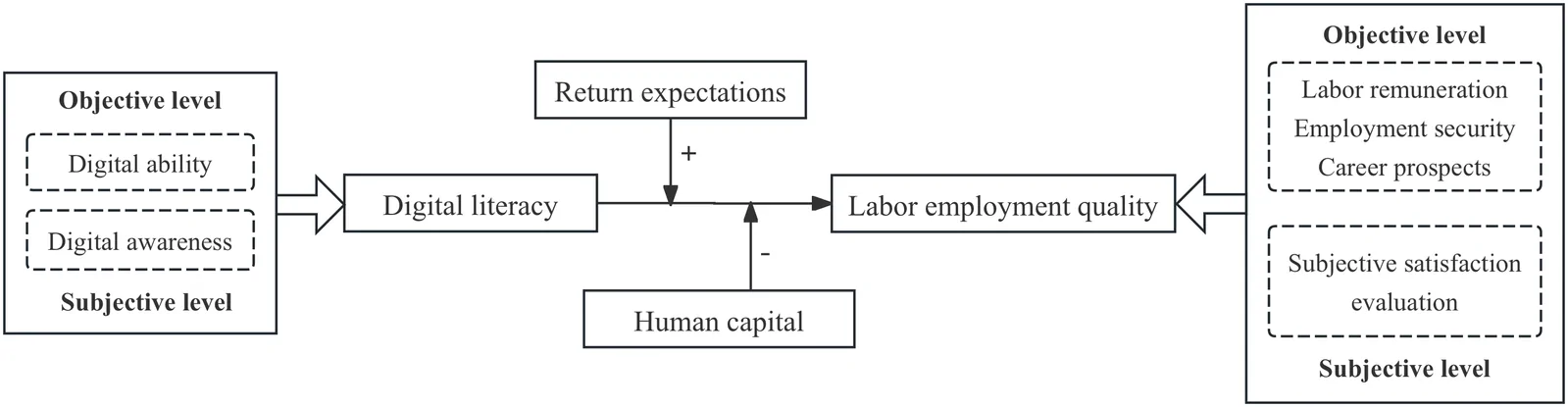
The empirical analysis framework. Note: Source: Authors’ construction.

Based on the aforementioned analysis, this study presents the empirical analysis framework illustrated in Fig 1. This framework not only delineates the direct path through which digital literacy influences employment quality (H1) and two crucial boundary conditions (H2 and H3), but also links it with the multi-dimensional objectives of sustainable employment development at the underlying logic level. Digital literacy directly contributes to the efficiency and stability of the labor market by improving individual employment quality (H1); the moderating effect of return expectations (H2) reveals the psychological motivation for sustaining investment in human capital; whereas the moderating effect of human capital (H3) emphasizes the potential of digital literacy to facilitate inclusive growth and reduce group disparities. The verification of these three hypotheses constitutes a systematic exploration, from the micro-individual level, of the impact of individual digital literacy on employment quality in the digital age. It visually presents a summary of the relationships among the core variables (digital literacy, employment quality, return expectations, human capital), along with the three major hypotheses (H1, H2, H3) that this study intends to test. Among these, the core explanatory variable—“digital literacy”—directly influences the dependent variable—“employment quality”—via the main path (corresponding to Hypothesis H1). The two moderating paths respectively illustrate the moderating effects of “return expectations” and “human capital” on this main effect (corresponding to Hypotheses H2 and H3). We hypothesize that “return expectations” positively strengthen the effect of digital literacy (denoted by “+”), whereas “human capital” negatively attenuates it (denoted by “−”). All analyses are carried out while controlling for a series of individual characteristic variables. This framework is designed to visualize the causal relationship network derived from the theoretical analysis section of this paper.

## Methodology and data

### Data source

This study utilizes data from the China Family Panel Studies (CFPS), administered by the Institute of Social Science Survey (ISSS) at Peking University. The CFPS adopts a multi-stage, multi-level random probability sampling approach across the country. The dataset encompasses various domains, including economics, sociology, demography, and education, and is characterized by strong sample representativeness. Given the specific selection of variables relevant to this research, the empirical analysis draws on data collected in 2022. The study focuses on individuals within the working-age population, specifically those aged between 18 and 60. After selecting the relevant indicators and excluding responses that were missing, abnormal, or indicated “do not know,” a final sample of 7,625 valid observations was obtained.

It is noteworthy that the CFPS data are publicly available for academic research upon application through its official website (http://www.isss.pku.edu.cn/cfps/). The authors accessed the 2022 wave of data under the terms of the CFPS data use agreement, which mandates compliance with confidentiality and usage regulations. The original CFPS survey protocol received ethical approval from the Biomedical Ethics Review Committee of Peking University. As this study involves secondary analysis of anonymized, publicly available data, it did not require additional institutional review board approval. All procedures performed in the original studies involving human participants were in accordance with the ethical standards of the institutional and national research committees and with the 1964 Helsinki Declaration and its later amendments or comparable ethical standards.

### Variable selection

#### Explained variable.

The selection of indicators for employment quality is grounded in the International Labour Organization’s ‘Decent Work’ framework [5], which emphasizes both objective conditions (remuneration, security, and career progression) and subjective perceptions (satisfaction with income, job security, work environment, working hours, and promotion prospects). We include labor remuneration indicators (wage, bonus, and fringe benefits) to capture economic returns; employment security indicators (labor contract, pension insurance, health insurance, and housing provident fund) to reflect job stability and social protection; and career prospects to capture upward mobility. Subjective satisfaction indicators are included to capture workers’ perceived well-being, which is increasingly recognized as a core dimension of employment quality [5]. All indicators are aligned with the multidimensional nature of employment quality and are commonly used in the existing literature [16]. Moreover, to address the limitations associated with relying on single indicators, this study employs the Entropy Method to construct a comprehensive employment quality index. The detailed evaluation framework and the weight of indicators are presented in Table 1.

**Table 1:** Employment quality evaluation system.

Dimension	Variable Description		Variable Assignment	Indicator weights
Objective level	Labor remuneration	Wage income	Log-transformed monthly after-tax wage (¥/month)	0.253
		Bonus income	Log-transformed monthly after-tax bonus (¥/month)	0.228
		Fringe benefits income	Log-transformed monthly after-tax fringe benefits amount (¥/month)	0.131
	Employment security	Labor contract	1 = Yes; 0 = No	0.049
		Pension insurance	1 = Yes; 0 = No	0.079
		Health insurance	1 = Yes; 0 = No	0.078
		Housing provident fund	1 = Yes; 0 = No	0.006
	Career prospects	Career promotion	Promoted in the past 12 months: 1 = Yes; 0 = No	0.069
Subjective level	Subjective satisfaction evaluation	Income satisfaction	1–5 (Very dissatisfied – Very satisfied)	0.032
		Job security perception	1–5 (Very dissatisfied – Very satisfied)	0.018
		Work environment satisfaction	1–5 (Very dissatisfied – Very satisfied)	0.015
		Working hours satisfaction	1–5 (Very dissatisfied – Very satisfied)	0.020
		Promotion prospects satisfaction	1–5 (Very dissatisfied – Very satisfied)	0.021

Note: Data source: China Family Panel Studies (CFPS), 2022 (the same applies to the following tables).

#### Core explanatory variable.

The selection of indicators for digital literacy is guided by established digital literacy frameworks, including UNESCO’s Global Digital Literacy Framework [11] and the European Union’s DigComp framework, which conceptualize digital literacy as a multidimensional construct encompassing both technical skills and cognitive awareness. The ‘digital ability’ dimension captures behavioral engagement with digital tools across four domains—learning, shopping, entertainment, and social activities—which reflect the breadth and diversity of digital tool utilization. Sustained and diverse engagement with digital tools is a fundamental prerequisite for and behavioral manifestation of skill acquisition and development [18]. The ‘digital cognition’ dimension captures subjective perceived importance of the Internet across the same domains, reflecting the motivational and evaluative dimension of digital literacy that influences the depth and purposefulness of technology use. This dual-dimensional approach—combining observed behavioral engagement with subjective cognitive appraisal—provides a more holistic approximation of an individual’s digital literacy than usage alone, acknowledging that in real-world contexts, skill is often exercised and demonstrated through purposeful use [20]. The detailed evaluation indicator system and indicator weights are presented in Table 2.

**Table 2:** Digital literacy evaluation system.

Dimension	Variable Description	Variable Assignment	Indicator weights
Digital ability	Online learning activities	1 = Yes; 0 = No	0.218
	Online shopping activities	1 = Yes; 0 = No	0.128
	Online entertainment activities	1 = Yes; 0 = No	0.364
	Online social activities	1 = Yes; 0 = No	0.073
Digital Application Cognition	The importance of using the internet for working	1–5 (Very unimportant – Very important)	0.065
	The importance of using the internet for learning	1–5 (Very unimportant – Very important)	0.056
	The importance of using the internet for entertainment	1–5 (Very unimportant – Very important)	0.041
	The importance of using the internet for social activities	1–5 (Very unimportant – Very important)	0.054

It is worth noting that the selection of indicators for “digital capabilities”—namely online learning, online shopping, online entertainment, and online social activities—requires conceptual justification. While these items reflect usage frequency or participation rather than directly assessing proficiency levels, they serve as valid proxy measures within the constraints of large-scale social survey data like the CFPS. Grounded in digital literacy theory, sustained and diverse engagement with digital tools is a fundamental prerequisite for and a behavioral manifestation of skill acquisition and development [21,22]. Furthermore, the inclusion of “digital cognition” (subjective perceived importance across the same domains) complements these behavioral measures by capturing the motivational and evaluative dimension of digital literacy, which influences the depth and purposefulness of technology use. This combined approach of observed behavioral engagement and subjective cognitive appraisal provides a more holistic approximation of an individual’s digital literacy than usage alone, acknowledging that in real-world contexts, skill is often exercised and demonstrated through purposeful use.

#### Moderating variables.

The moderating variables in this study are return expectations and human capital. Return expectations refer to an individual's subjective belief regarding whether their efforts will lead to commensurate rewards and whether they can improve their living conditions through personal actions [30]. These expectations affect the extent to which individuals are motivated to invest in skill development, such as improving digital literacy. When individuals believe that their efforts will be recognized and rewarded, they are more likely to translate digital literacy into enhanced employability actively. Drawing on existing research, this study utilizes two items from the database—“In today’s society, hard work will be rewarded” and “In today’s society, people like me have a great chance to improve their living standards”—to measure individuals’ expectation of return. Both items are rated on a five-point Likert scale (1 = Strongly Disagree, 5 = Strongly Agree), and the expectation of return is calculated by averaging the scores of the two items. Human capital refers to the total knowledge and skills accumulated by an individual through education, training, experience, and other related factors, reflecting their overall ability [31]. According to Becker’s theory of human capital, formal education constitutes a primary pathway for human capital accumulation, as it directly influences workers’ skill development and productivity [32]. Moreover, educational attainment is an objective and quantifiable indicator that allows for cross-group comparisons. Therefore, this study adopts educational attainment as a proxy variable for human capital.

#### Control variables.

Based on existing research [33,34], this study incorporates five control variables: gender, age, marital status, political affiliation, and household registration type. Detailed definitions and descriptive statistics for these variables are provided in Table 3.

**Table 3:** Variable definition and descriptive statistics.

Variable		Variable Description	Mean	Std. Dev.	Min.	Max.	Med.
Explained variable	Digital literacy	Comprehensive index calculated using the entropy method	0.584	0.256	0.028	0.999	0.580
Core explanatory variable	Employment quality	Comprehensive index calculated using the entropy method	0.973	0.224	0.015	1.551	0.978
Moderator variables	Expected returns	Mean score of measurement items	3.261	0.561	1	5	3
	Human capital	Educational attainment: 1 = Primary school or below; 2 = Junior high school; 3 = Senior high school; 4 = College or above	2.650	1.098	1	4	3
Control variables	Gender	1 = Male; 0 = Female	0.557	0.497	0	1	1
	Age	Actual age	37.989	10.491	18	60	36
	Marital status	1 = Married; 0 = Unmarried	0.753	0.431	0	1	1
	Political status	1 = Communist Party member; 0 = Non-Communist Party member	0.120	0.325	0	1	0
	Household registration type	1 = Non-agricultural; 0 = Agricultural	0.371	0.483	0	1	0

### Model specification

#### Comprehensive index construction method.

The entropy weight method is an objective weighting approach in which the weights are solely determined by the degree of dispersion (information entropy) of the data for each indicator. This approach avoids the problem in principal component analysis (PCA) and exploratory factor analysis (EFA), where the weights are predominantly influenced by the linear correlation structure among variables, and the factor meanings may require post-hoc interpretation. When calculating the comprehensive index, this method enables an accurate reflection of the true information contribution of each indicator within the preset dimension framework, without being distorted by collinearity among variables. Moreover, the indicator system of this study encompasses continuous variables (e.g., the logarithm of salary), binary dummy variables (e.g., whether a contract is signed), and ordered categorical variables (e.g., satisfaction scores), which have different measurement scales and distributions. The entropy weight method conducts calculations based on standardized data and has no strict requirements for the distribution shape of the indicators. Therefore, it is highly suitable for mixed-type data. In contrast, PCA and EFA are generally more applicable to continuous variables that approximately follow a multivariate normal distribution. Directly applying these methods to the mixed-type data in this study may challenge their underlying assumptions and compromise the robustness of the results. Taking the above factors into account, this study ultimately decides to construct a comprehensive index of digital literacy and employment quality using the entropy weight method. The calculation steps are as follows:

Step 1: Dimensional normalization processing. To eliminate scale effects, we normalize each indicator using min – max normalization:


$$\begin{array}{c} X_{ij}=\left\{ \begin{array}{c} @l\frac{x_{ij}-min\left(x_{ij}\right)}{max\left(x_{ij}\right)-min\left(x_{ij}\right)},\;ifx_{ij}isapositiveindicator\\ \frac{max\left(x_{ij}\right)-x_{ij}}{max\left(x_{ij}\right)-min\left(x_{ij}\right)},\;ifx_{ij}isanegativeindicator \end{array}\right. \end{array}$$ (1)

Step 2: Calculation of entropy value. For each indicator *j*, we calculate the entropy value *d*_*j*_ as:


$$\begin{array}{c} d_{j}=\sum_{i=\text{1}}^{n}\left\{\frac{X_{ij}}{\int_{n=\text{1}}^{n}X_{ij}}\times ln\frac{X_{ij}}{\int_{n=\text{1}}^{n}X_{ij}}\right\} \end{array}$$ (2)

Step 3: Determination of indicator weights. The weight *W*_*j*_ for indicator *j* is calculated as:


$$\begin{array}{c} W_{j}=\frac{\text{1}-d_{i}}{\sum_{j=\text{1}}^{m}\left(\text{1}-d_{i}\right)} \end{array}$$ (3)

Step 4: Calculation of the comprehensive evaluation index. The final composite index *S*_*i*_ for each individual *i* is calculated as:


$$\begin{array}{c} S_{i}=\sum_{j=\text{1}}^{m}\left(W_{j}\times X_{ij}\right) \end{array}$$ (4)

In Equations (1)–(4), *X*_*ij*_ denotes the dimensionless normalized value of the *j*th evaluation index for the *i*th research unit; *n* represents the number of research units, and *m* represents the number of indicators; *d*_*j*_ is the entropy value of the *j*th indicator; *W*_*j*_ is the weight of the *j*th indicator; *S*_*i*_ is the comprehensive evaluation index of the *i*th research unit.

#### Benchmark regression model.

Considering that the dependent variable in this research is continuous, the Ordinary Least Squares (OLS) regression method is employed to establish the baseline model for examining the impact of digital literacy on labor employment quality. The model is specified as follows:


$$\begin{array}{c} Y_{i}=\alpha_{\text{0}}+\alpha_{\text{1}}X_{i}+\alpha_{\text{2}}C_{i}+\epsilon_{i} \end{array}$$ (5)

In the equation (5), *Y*_*i*_ denotes the employment quality of individual labor force *i*, serving as the dependent variable; *X*_*i*_ denotes the digital literacy level of individual labor force *i*, serving as the independent variable; *C*_*i*_ denotes a set of control variables that are associated with the employment quality of individual labor force *i*. *a*_*0*_ is the intercept term, while *a*_*1*_ and *a*_*2*_ are the parameters to be estimated, and $\epsilon_{i}$ represents the random error term. In all OLS regressions, robust standard errors are employed to ensure the reliability of statistical inference. Furthermore, it is important to note that the benchmark regression model employed in this study does not account for regional fixed effects (e.g., provinces or the eastern, central, and western regions). This design is based on the following considerations: One of the primary objectives of this study is to subsequently investigate the regional heterogeneity of the impact of digital literacy. If regional fixed effects were controlled for in the benchmark model, it would imply that the marginal effect of digital literacy is uniform across different regions, which contradicts the research objective and would obscure potential heterogeneity. Therefore, the benchmark model is intended to estimate the overall average impact of digital literacy on employment quality, while deferring the in-depth analysis of regional differences to subsequent group regressions—a more straightforward and interpretable approach.

#### Moderation effect model.

To investigate the moderating effects of return expectations and human capital on the relationship between digital literacy and labor force employment quality, interaction terms were introduced—specifically, the interaction between digital literacy and return expectations, and the interaction between digital literacy and human capital, respectively. Subsequently, a regression analysis was performed on the moderating effect model incorporating these interaction terms. The model is specified as follows:


$$\begin{array}{c} Y_{i}=\beta_{\text{0}}+\beta_{\text{1}}X_{i}+\beta_{\text{2}}X_{i}Z_{i}+\beta_{\text{3}}C_{i}+\varepsilon_{i} \end{array}$$ (6)

$$\begin{array}{c} Y_{i}=\omega_{\text{0}}+\omega_{\text{1}}X_{i}+\omega_{\text{2}}X_{i}Z_{i}+\omega_{\text{3}}C_{i}+\epsilon_{i} \end{array}$$ (7)

In the formula (6)-(7), *Z*_*i*_ denotes the adjustment variable, representing the expected return of labor force *i* and the status of human capital; $\beta_{\text{0}}$ and $\omega_{\text{0}}$ are constant terms. $\beta_{\text{1}}$*,* $\beta_{\text{2}}$*,* $\beta_{\text{3}}$*,* $\omega_{\text{1}}$*,* $\omega_{\text{2}}$*,* and $\omega_{\text{3}}$ are parameters to be estimated. The meanings of the remaining variable symbols are consistent with those described above.

## Results and analysis

### Multicollinearity test

Prior to conducting the regression analysis, this study carried out a variance inflation factor (VIF) diagnosis on all variables to examine whether the model suffered from severe multicollinearity. Generally speaking, if the VIF value exceeds 10, it indicates severe multicollinearity. As presented in Table 4, the VIF values of all variables are significantly lower than 10, with an average VIF of merely 1.240. These findings suggest that the regression models in this study were not affected by severe multicollinearity, and the subsequent parameter estimates are stable and reliable.

**Table 4:** VIF test results.

Variables	Digital literacy	Gender	Age	Expected returns	Mean value of VIF
VIF	1.310	1.030	1.530	1.010	
Variables	Marital status	Political status	Household registration type	Human capital	1.240
VIF	1.260	1.120	1.230	1.450	

#### The impact of digital literacy on employment quality of the labor force.

This study employed Stata 17.0 software for data analysis. Table 5 presents the benchmark regression results regarding the impact of digital literacy on labor employment quality. Model (1) examines the bivariate relationship between digital literacy and labor employment quality, while Model (2) incorporates additional control variables that may influence labor employment quality. Across all regression results, the coefficient for the digital literacy variable remains positive and statistically significant at the 1% level. These findings indicate that digital literacy contributes to the enhancement of labor employment quality, thereby supporting the verification of Hypothesis H1.

**Table 5:** Benchmark regression results.

Variable	Employment Quality	
	Model (1)	Model (2)
Digital literacy	0.154***	0.119***
	(0.010)	(0.011)
Gender		−0.025***
		(0.005)
Age		0.001
		(0.003)
Marital status		0.016**
		(0.007)
Political status		0.037***
		(0.008)
Household registration type		0.047***
		(0.005)
Constant	0.883***	0.979***
	(0.006)	(0.077)
N	7,625	7,625
R^2^	0.031	0.048

Note: Significance at the 1%, 5%, and 10% levels are expressed by ***, **, and *, respectively; Robust standard errors are presented in parentheses (the same applies to the following tables).

#### Robustness test.

To verify the robustness of the benchmark regression results, three alternative approaches were employed: First, to examine whether the benchmark regression results are sensitive to the choice of weighting method, we re-estimated the composite index of digital literacy and employment quality using factor analysis, where weights were derived from the factor loadings. Subsequently, we re-ran the benchmark regression analysis using these alternative indices (see Table 6, Model (3)). The results show that in Model (3), the coefficient on digital literacy remains positive and statistically significant at the 1% level. These findings demonstrate that the main conclusions of this paper are robust to different weighting schemes and are not driven by the use of entropy weights; Second, Model (4) is derived by adjusting the sample size—specifically, by randomly selecting 90% of the original sample and repeating the regression process; Third, Model (5) performs a regression analysis after removing the extreme values in the top and bottom 1% of the sample data to mitigate the impact of potential outliers. The corresponding results are presented in Models (3) to (5) of Table 6. The findings reveal that the correlation and statistical significance of digital literacy with employment quality remain largely unchanged across all robustness models. This consistency supports the robustness and reliability of the benchmark regression results, thereby confirming the validity of Hypothesis 1.

**Table 6:** Robustness test and instrumental variable test.

Variable	Replace the Explained Variable	Change the Sample Size	Winsorization	Digital Literacy	Employment Quality
	Model (3)	Model (4)	Model (5)	Model (6)	Model (7)
Digital literacy	1.040***	0.116***	0.092***		0.318***
	(0.043)	(0.012)	(0.010)		(0.040)
Instrumental variable				0.056***	
				(0.002)	
Control variable	Yes	Yes	Yes	Yes	Yes
Constant	−1.853***	0.934***	0.950***	0.034	0.947***
	(0.312)	(0.081)	(0.074)	(0.078)	(0.078)
F-value				597.53	
LM-value				488.28***	
N	7,625	6,862	7,444	7,625	7,625
R^2^	0.231	0.047	0.037		

Note: Model (6) showcases the first-stage regression results of the instrumental variable approach, and Model (7) showcases the corresponding second-stage results.

### Endogenous discussion

#### Propensity score matching (PSM).

To address potential endogeneity issues arising from self-selection bias in digital literacy, the propensity score matching (PSM) approach is employed for endogeneity testing. Worker employment quality is operationalized as a binary variable: observations with values greater than or equal to the sample mean are assigned to the treatment group (coded as 1), while those below the mean are classified into the control group (coded as 0). The average treatment effect on the treated (ATT) is estimated using both radius matching and kernel matching techniques. As shown in Table 7, enhanced digital literacy demonstrates a statistically significant positive impact on employment quality, aligning with the findings from the benchmark regression analysis. The aforementioned results suggest that although the binarization of continuous variables using the PSM method does result in a certain degree of information loss, this approach focuses on testing “probability differences.” Moreover, its conclusion aligns with those of the benchmark regression and the instrumental variable method, collectively strengthening the robustness of the research findings.

**Table 7:** Results of PSM.

Variable	Method	Treatment Group	Control Group	ATT	S.E.	t-Value
Employment quality	Radius matching	0.621	0.580	0.041***	0.006	6.86
	kernel matching	0.621	0.575	0.046***	0.006	7.67

#### Instrumental variable test.

To avoid potential endogeneity issues caused by omitted variables, an instrumental variable (IV) approach was employed for further testing. The instrumental variable selected for digital literacy was “Perceived value of the Internet,” which was measured using the survey item: “Personal perception of the importance of the Internet in daily life.” This paper posits that the subjective attitude of “individuals’ perception of the significance of the Internet in daily life” indirectly influences their performance in the labor market (employment quality), primarily through its impact on individuals’ investment in and learning of digital technologies (i.e., digital literacy).

The exclusion restriction requires that the instrument has no direct effect on employment quality other than through digital literacy. We argue that this condition is plausibly satisfied for three interconnected reasons.

First, the perceived value of the Internet is strongly correlated with the endogenous explanatory variable, digital literacy. Individuals who regard the Internet as highly important in their daily routines are more likely to engage actively with digital devices and online platforms, thereby accumulating practical digital skills and knowledge through routine use, self-directed learning, or social interaction. This positive association between perceived importance and actual digital competence has been well-documented in prior literature [4,12]. Second, the perceived value of the Internet affects the quality of employment solely by influencing digital literacy, and not through any other direct means. An individual's general attitude toward the Internet's importance in daily life is a broad, non‑specific psychological predisposition, rather than a targeted evaluation of its role in career advancement. Such a general perception is unlikely to directly determine specific labor market outcomes such as wage levels, the existence of a labor contract, social security coverage, or promotion opportunities—these are predominantly shaped by structural market factors, individual professional skills, work experience, and industry and occupation‑specific characteristics [18]. Third, we emphasize that this variable measures “importance in daily life” rather than specifically “importance in career development,” which conceptually differentiates it from direct labor market drivers. While a person who values the Internet highly may also be more motivated to improve digital literacy, that same perception does not, in itself, influence employers’ hiring, contracting, or promotion decisions, nor does it affect job characteristics such as industry or occupational rank, except through the digital skills it fosters, thereby satisfying the exclusion restriction required for a valid instrument [20]. Specifically, the instrumental variable should be correlated with the endogenous explanatory variable (digital literacy) and uncorrelated with the error term.

The IV estimation results are reported in Models (6) and (7) of Table 6. The findings indicate that the instrumental variable is significantly positively correlated with digital literacy. Moreover, the first-stage F-statistic is 597.53, which exceeds the critical value of 16.38 at the 10% significance level, thereby effectively eliminating the presence of weak instrumental variables [35]. The LM statistic is significant at the 1% significance level, indicating that the instrumental variable passes the test for underidentification. After correcting for endogeneity bias, digital literacy continues to exhibit a statistically significant positive association with employment quality in the labor market, and the absolute value of the coefficient increases. This further substantiates the findings of the benchmark regression.

While the exclusion restriction remains empirically untestable, we believe the theoretical reasoning provided above offers a credible basis for its plausibility. We also acknowledge this limitation explicitly in the Discussion section and call for future research using more exogenous instruments.

#### Moderating effect test.

To investigate the boundary conditions of digital literacy’s impact on labor force employment quality, this study incorporated interaction terms between digital literacy and return expectations, as well as digital literacy and human capital, to examine potential moderating effects. The results are presented in Models (8) and (9) of Table 8. The findings reveal that the interaction term between digital literacy and return expectations exhibits a positive and statistically significant coefficient at the 10% level. This result implies that an individual's conviction that “effort can be rewarded” might serve as a psychological impetus, augmenting their incentive to transform digital skills into tangible employment benefits. The relatively low statistical significance could indicate that the magnitude of this moderating effect is moderate within the entire population or is influenced by measurement inaccuracies. Nevertheless, when considered in conjunction with the heterogeneity finding that the effect is more pronounced in the western and urban agglomeration regions where the perception of return expectations might be higher, the cumulative evidence in this study tends to corroborate the positive moderating role of return expectations. Future research can employ more sophisticated expectation measurements or experimental designs to further validate and quantify the strength of this psychological mechanism. In contrast, the interaction term between digital literacy and human capital shows a negative and statistically significant coefficient at the 1% level. Following the standard approach for assessing moderating effects [36], it can be concluded that return expectations positively moderate the influence of digital literacy on employment quality, while human capital negatively moderates this relationship. These results support Hypotheses H2 and H3. Specifically, higher return expectations enhance the likelihood that individuals will actively leverage digital literacy to improve their employment competitiveness, thereby strengthening the positive effect of digital literacy on employment quality. Conversely, as the educational attainment of the labor force increases, the marginal benefit of digital literacy diminishes, as individuals with higher human capital have already developed strong employment capabilities through educational investments. In contrast, individuals with lower human capital tend to rely more heavily on digital literacy to compensate for skill deficiencies and enhance their employment quality.

**Table 8:** The results of the moderation effect test.

Variable	Employment Quality	
	Model (8)	Model (9)
Digital literacy	0.055	0.130***
	(0.036)	(0.014)
Expected returns	0.028***	
	(0.008)	
Human capital		0.054***
		(0.004)
Digital literacy × Expected returns	0.020*	
	(0.011)	
Digital literacy × Human capital		−0.013***
		(0.002)
Control variable	Yes	Yes
Constant	0.867***	0.962***
	(0.080)	(0.076)
N	7,625	7,625
R^2^	0.059	0.078

### Heterogeneity analysis

China's regional development exhibits notable gradient differences [37]. Systematic disparities exist among the eastern, central, and western regions in terms of the density of digital infrastructure and industrial structure, resulting in regional variations in the marginal benefits associated with laborers’ digital literacy. To address this, this study categorizes the samples into three major regions—eastern, central, and western—based on the classification criteria of the National Bureau of Statistics. It further investigates the regional heterogeneity in the impact of digital literacy on employment quality. The findings are presented in Model (10) to Model (12) of Table 9, which reveal that digital literacy exerts the most significant positive influence on employment quality in the western region, followed by the eastern region, with the least impact observed in the central region. One possible explanation is that the digital infrastructure in the western region is currently undergoing rapid development, resulting in a relatively high marginal return on workers’ digital skills. The acquisition of basic digital competencies can confer a significant competitive advantage in the local labor market. In contrast, the eastern region exhibits a highly mature digital economy, where workers generally already possess a baseline level of digital proficiency. Consequently, the marginal benefits associated with further improvements in digital literacy tend to diminish. In the central region, structural challenges persist in the process of industrial transformation. Traditional manufacturing sectors exhibit limited demand for advanced digital skills, while emerging service industries have not yet achieved full market penetration. As a result, the marginal contribution of digital literacy to overall employment quality is lowest in this region [20,22,38].

**Table 9:** Results of heterogeneity analysis.

Variable	Eastern Region	Middle Region	Western Region	Urban Agglomerations	Non-Urban Agglomerations
	Model (10)	Model (11)	Model (12)	Model (13)	Model (14)
Digital literacy	0.127***	0.083***	0.138***	0.132***	0.111***
	(0.015)	(0.021)	(0.022)	(0.019)	(0.013)
Control variable	Yes	Yes	Yes	Yes	Yes
Constant	0.964***	0.962***	1.142***	1.095***	0.952***
	(0.107)	(0.159)	(0.153)	(0.134)	(0.093)
N	3,983	1,850	1,792	2,521	5,104
R^2^	0.047	0.040	0.055	0.057	0.042

To further elucidate the influence of resource agglomeration on digital literacy, this study categorized the Beijing-Tianjin-Hebei region, the Yangtze River Delta, and the Pearl River Delta as urban agglomerations, while other cities were grouped as non-urban agglomerations. The regional heterogeneity in the impact of digital literacy on employment quality was analyzed. The findings are presented in Model (13) and Model (14) of Table 9. The results demonstrate that the positive effect of digital literacy on labor employment quality is significantly more substantial in urban agglomeration areas compared to non-urban agglomeration areas. Urban agglomerations serve as central hubs for regional economic and digital resource concentration, with industrial structures dominated by high-end service and high-tech industries. Consequently, the demand for digital skills and the availability of relevant application scenarios are markedly higher in these areas [39]. In contrast, non-urban agglomeration areas are characterized by a larger share of traditional industries, slower digital transformation, and fewer opportunities for the application of digital literacy, which results in a relatively weaker impact on employment quality compared to urban agglomeration areas [40].

## Discussion

This study utilizes CFPS data to conduct an empirical investigation into the relationship between digital literacy and the quality of labor employment, as well as the underlying mechanisms. Our findings reveal that digital literacy is significantly positively associated with labor employment quality, underscoring the growing importance of digital competencies in the contemporary labor market. Furthermore, through robustness checks and discussions on endogeneity, we confirm the stability and reliability of this association. The research outcomes offer valuable insights for policymakers and educational institutions, highlighting that improving workers’ digital literacy is a crucial strategy for enhancing employment outcomes amid digital transformation. These findings not only align with existing academic literature but also contribute novel empirical evidence to the field, providing theoretical support for further exploration of micro-level labor market dynamics during the digital transformation era.

Firstly, this study confirmed the positive impact of digital literacy on the improvement of labor employment quality by utilizing representative data, and the reliability of causal inference was enhanced through robustness tests and discussions on endogeneity, leading to conclusions consistent with those of numerous scholars. For example, existing literature suggests that various aspects of digital literacy, such as digital skills and internet usage, can increase labor remuneration by expanding employment opportunities and facilitating cross-regional labor mobility [41], or enhance employment stability by improving the efficiency of information utilization [42]. However, in contrast to previous studies that typically characterized digital literacy merely through indicators such as internet usage and digital technology application [43,44], this study innovatively proposed a dual-dimensional framework of “objective digital ability – subjective digital awareness.” A comprehensive index was then constructed using the entropy method to quantitatively assess the level of digital literacy, thereby offering a more holistic understanding of its role. At the same time, this study extends beyond traditional research that has primarily focused on objective aspects of employment quality, such as labor remuneration [20]. It systematically integrates key indicators—including employment security, career development prospects, and employment satisfaction—into the analysis, thereby offering a more comprehensive understanding of how digital literacy enhances overall employment quality. This approach provides micro-level empirical support for macro-level policies aimed at digitally empowering the workforce.

Next, this study employed moderating effect analysis to deconstruct the impact mechanisms of digital literacy. Firstly, the positive moderating effect of return expectation (H2 is supported) highlights the “catalyst” role of psychological motivation. The significant positive interaction term is consistent with the view that for workers who firmly believe that “efforts will be rewarded,” the association between digital skills and employment benefits may be stronger. This finding is highly consistent with expectancy theory [45], which connects micro-level individual psychological expectations with macro-level skill investment behavior. In practice, this implies that if digital training programs can be integrated with clear career prospects and successful case demonstrations to stimulate learners’ positive expectations, they will be more effective in converting skills into actual improvements in employment quality [46,47]. This offers important implications for the design of “incentive-compatible” skill intervention policies. Secondly, the negative moderating effect of human capital (H3) reveals a phenomenon with significant policy implications. The significant negative interaction term indicates that the positive association between digital literacy and employment quality is more pronounced among workers with lower education levels and weaker traditional human capital. This challenges the simplistic assumption that the returns to digital skills increase linearly with educational attainment [22]. One plausible explanation for the negative moderating effect of human capital is the presence of ceiling effects. For workers with high educational attainment—particularly those holding college degrees or above—their employment quality may already be near the upper bound of the distribution due to their strong human capital endowments. In such cases, the marginal contribution of digital literacy to further employment quality gains is naturally constrained by the limited remaining room for improvement. This interpretation is consistent with the concept of diminishing marginal returns to skill investment, which predicts that additional skill accumulation yields progressively smaller benefits as individuals approach higher competence levels [48]. However, ceiling effects alone cannot fully explain the observed pattern. If the negative moderation were driven solely by ceiling effects, we would expect the relationship between digital literacy and employment quality to be weakest among the highest‑educated workers but not necessarily stronger among the lowest‑educated workers. Our results, however, show a particularly pronounced positive association among low‑human‑capital workers, suggesting that digital literacy actively compensates for skill deficits rather than merely operating in a context of low baseline employment quality. This ‘skill compensation’ mechanism is an important complement to the ceiling effect explanation and underscores the potential of digital upskilling to reduce employment inequality.

Finally, this study conducted a heterogeneity analysis based on spatial logic, uncovering new regional differentiation patterns in how digital literacy influences the quality of labor employment. The findings align with the digital divide theory and the theory of resource agglomeration [49,50]. In terms of regional gradients, the positive association of digital literacy with employment quality is most pronounced in the western region, followed by the eastern region, and relatively least significant in the central region. This contradicts the intuitive assumption that “the more developed the economy, the higher the return,” yet it aligns with the theory of marginal returns of digital infrastructure [51]. In the western region, where digital infrastructure is in the catch-up phase, workers can acquire a substantial competitive edge by mastering basic digital skills, yielding high marginal returns. In the eastern region, where the digital economy is well-established, the penetration rate of digital skills is high and competition is intense, leading to a relatively smaller excess return from further improvement. In the central region, which is in the “difficult period” of industrial transformation and upgrading, the demand for high-level digital skills in traditional industries is inadequate, and emerging industries have not fully matured, resulting in relatively limited application opportunities for digital skills and impeding their value realization [38]. This pattern implies that digital literacy policies must be precisely tailored to the regional development stage and industrial structure.

In the context of agglomeration patterns, the intensity of the effect in urban agglomeration regions is notably higher than that in non-urban agglomeration regions. This emphasizes the amplification effect of resource agglomeration [52,53]. Urban agglomerations are not merely the “high ground” of digital infrastructure but also the “incubators” and “clusters” of new business models and new occupations in the digital economy. The dense industrial network, frequent knowledge dissemination, and abundant application scenarios offer a favorable environment for the realization of the value of digital skills. Conversely, the digital transformation of industries in non-urban agglomeration regions progresses relatively slowly, and the application scenarios of digital skills are restricted, thus diminishing their role in enhancing employment quality. This highlights the significance of leveraging urban agglomerations as hubs to promote the digital transformation and coordinated development of digital talents in the surrounding areas.

However, this study has certain limitations that warrant further investigation. Firstly, the measurement of digital literacy, constrained by data availability, primarily captures the breadth and frequency of digital tool usage (e.g., online activities) and subjective awareness, rather than directly assessing granular cognitive or technical skills (e.g., coding, data analysis, cybersecurity practices, or digital content creation). While we have conceptually justified these behavioral indicators as foundational proxies that correlate with and enable skill application, they cannot fully represent the depth or sophistication of an individual’s digital competencies. This reliance on self-reported usage frequency and perceived importance introduces potential measurement biases. Future research would benefit from employing specialized assessments or surveys incorporating items aligned with detailed digital literacy frameworks to more precisely measure specific skill dimensions and their differential impacts on various facets of employment quality.

Secondly, similar measurement considerations apply to our dependent variable. The multi-dimensional index of employment quality, while an advancement, is also based on self-reported data for key components like income, satisfaction, and promotion prospects. Self-reported income may be subject to recall error or rounding, and subjective satisfaction measures can be influenced by transient affective states or individual response tendencies. The potential for common method variance, given that both core variables are derived from the same survey respondent, cannot be entirely ruled out, although the use of composite indices and the inclusion of objective indicators (e.g., contract status, insurance) mitigate this concern to some extent. Future studies could strengthen measurement by linking survey data with administrative records (e.g., social security data for income and contract details) or employing multi-source evaluations.

Thirdly, our empirical strategy primarily relies on cross-sectional observational data and instrumental variable estimation, which, despite our efforts to address endogeneity, still rests on the untestable exclusion restriction assumption. Therefore, we interpret our main findings as associational evidence rather than definitive causal effects. While we have provided theoretical and empirical justifications for the instrument's validity, we acknowledge that the IV approach cannot fully rule out all alternative explanations. Future research could further strengthen causal identification by exploiting natural experiments or employing more exogenous instruments—such as historical or early-stage regional disparities in digital infrastructure construction—which would allow for a more rigorous assessment of the causal mechanisms underlying the associations we observe.

Fourthly, our empirical strategy primarily relies on cross‑sectional observational data from the 2022 CFPS and OLS regressions. While we have employed propensity score matching and instrumental variable estimation to mitigate endogeneity concerns, these methods do not fully eliminate the possibility of unobserved confounding or reverse causality. The IV approach, in particular, rests on the untestable exclusion restriction assumption, which we have justified on theoretical grounds but cannot verify directly. Therefore, we interpret all our findings as associative evidence rather than causal effects. Readers and policymakers should exercise caution when attributing changes in employment quality to improvements in digital literacy based solely on this study.

### Conclusions and implications

Based on the CFPS database, this study empirically investigates the association between digital literacy and labor force employment quality and the underlying mechanisms. The main findings are summarized as follows:

Digital literacy is significantly positively associated with the employment quality of the labor force. Higher levels of digital literacy correspond to better overall employment quality, and these results remain robust following various robustness checks and endogeneity analyses.

Return expectations positively moderate the relationship between digital literacy and employment quality, whereas human capital negatively moderates this effect. Specifically, higher return expectations and lower levels of human capital amplify the positive impact of digital literacy on employment quality.

The effects of digital literacy on employment quality exhibit heterogeneity across regions. From the perspective of regional development, the observed positive relationship is most potent in the western region, followed by the eastern region, with the weakest effect observed in the central region. From the perspective of resource agglomeration, the positive impact of digital literacy on employment quality is significantly greater in urban agglomeration areas compared to non-agglomeration areas.

Given the correlational nature of our empirical evidence, the following policy implications should be interpreted as preliminary and tentative. They are intended to stimulate discussion and inform hypothesis generation for future evaluation, rather than to serve as definitive causal prescriptions. Any actual policy design should be preceded by rigorous pilot testing and contextual feasibility assessments.

Based on these associative findings, we offer the following tentative policy considerations:

Firstly, it may be worthwhile to consider formulating precise and incentive-compatible intervention programs tailored to different human capital groups. Considering that the positive correlation between digital literacy and employment quality is more pronounced among low-human-capital (low educational attainment) workers, policymakers could give priority to and concentrate on this group. It may be advisable to design and implement “digital skills compensation” training programs that closely align basic and advanced digital skills with specific and localized non-agricultural job requirements. Such programs could help address their traditional skill shortages and potentially maximize the utility of digital literacy as a “tool for crossing the employment threshold.” Simultaneously, to potentially improve training conversion efficiency, “incentive modules” could be systematically incorporated into the courses. This might involve presenting real-life cases of learners with similar backgrounds who have successfully enhanced their employment quality through improving digital skills, offering clear career development path guidance, and exploring linkages between training certificates and employment recommendations, small-scale entrepreneurship support, and other incentive policies—thus possibly increasing participants’ expected returns and stimulating their intrinsic motivation to learn and apply digital skills.

Secondly, policymakers may consider implementing a differentiated strategy to enhance digital literacy and promote industrial development in a coordinated manner. In the western regions, considering that the digital infrastructure is in a catch-up stage and the marginal returns of digital skills are high, two aspects deserve policy attention: First, expediting the popularization of broadband networks and public digital service platforms and lowering the access threshold. Second, vigorously launching the “Basic Digital Skills Popularization Campaign,” focusing on training practical skills related to the digital transformation of local characteristic industries, so as to potentially improve the competitiveness of workers in the local labor market. Meanwhile, in the developed eastern regions, the policy focus might transition from “popularization” to “deepening” and “certification.” Promoting the establishment of an advanced digital skills certification system, encouraging enterprises, educational institutions, and training organizations to offer relevant courses, and moderately linking them to salary systems and professional title evaluations could help meet the demand for high-level digital talents in industrial upgrading and assist workers in obtaining potential returns. Additionally, considering the characteristics of industrial structure transformation in the central regions, policies could emphasize “demand-driven” approaches. In combination with local manufacturing upgrade plans and service industry development initiatives, tailoring digital skills training content—such as intelligent equipment operation, industrial internet applications, and digital cultural and creative industries—may better align skill supply with future industrial demands and facilitate the realization of digital skill value.

Thirdly, it may be beneficial to utilize the radiation effect of urban agglomerations to construct cross-regional hubs for digital talent development and resource sharing. Fully capitalizing on the advantages of major urban agglomerations—such as the Beijing-Tianjin-Hebei region, the Yangtze River Delta, and the Pearl River Delta—as digital resource hubs, supporting core cities in these urban agglomerations to establish “Digital Talent Training and Exchange Centers” and develop high-quality, standardized training courses and online learning platforms could be considered. Through a “pairing assistance” mechanism, encouraging these centers to export course resources, teaching personnel, and training opportunities to non-agglomeration areas in surrounding regions may help narrow regional gaps. Simultaneously, promoting the mutual recognition of digital skills certifications and facilitating talent mobility within and among urban agglomerations could ensure that returns on digital skills are not confined to local areas but can be realized through broader job markets, thereby potentially amplifying the positive implications of improving digital literacy.

## Supporting information

S1 Text Data. (ZIP)

## References

[1] Zeng X, Chu S, Chen X. China’s labor market demand in the shadow of COVID-19: Evidence from an online job board. J Asian Econ. 2025;97:101885. doi: 10.1016/j.asieco.2025.101885

[2] Li W, Chen Y. The Digital Economy and Gender Disparities in Rural Non-Agricultural Employment: Challenges or Opportunities for Sustainable Development? Sustainability. 2025;17(9):3911. doi: 10.3390/su17093911

[3] Zhou Y, Shi X. How does digital technology adoption affect corporate employment? Evidence from China. Econ Modell. 2025;147:107045. doi: 10.1016/j.econmod.2025.107045

[4] Eshet-Alkalai Y. Thinking in the Digital Era: A Revised Model for Digital Literacy. IISIT. 2012;9:267–76. doi: 10.28945/1621

[5] Blustein DL, Lysova EI, Duffy RD. Understanding Decent Work and Meaningful Work. Annu Rev Organ Psychol Organ Behav. 2023;10(1):289–314. doi: 10.1146/annurev-orgpsych-031921-024847

[6] Wang S, Qu C, Yin L. Digital literacy, labor migration and employment, and rural household income disparities. Int Rev Econ Fin. 2025;99:104040. doi: 10.1016/j.iref.2025.104040

[7] Galperin H, Arcidiacono M. Employment and the gender digital divide in Latin America: A decomposition analysis. Telecommun Policy. 2021;45(7):102166. doi: 10.1016/j.telpol.2021.102166

[8] Martin A, Grudziecki J. DigEuLit: Concepts and tools for digital literacy development. Innov Teach Learn Inform Comput Sci. 2006;5(4):249–67. doi: 10.11120/ital.2006.05040249

[9] Nedungadi PP, Menon R, Gutjahr G, Erickson L, Raman R. Towards an inclusive digital literacy framework for digital India. ET. 2018;60(6):516–28. doi: 10.1108/et-03-2018-0061

[10] Nulens G. The digital divide and development communication theory. 2003. doi: 10.1080/02500160308538021

[11] Feerrar J. Development of a framework for digital literacy. RSR. 2019;47(2):91–105. doi: 10.1108/rsr-01-2019-0002

[12] Wang X, Yi F, Yang C. “Promoting employment, supporting intelligence first”: the impact of digital literacy on non-farm employment of rural labour. Appl Econ Lett. 2024;31(19):2020–5. doi: 10.1080/13504851.2023.2209306

[13] Nguyen TP. Employability Discourse in Digital Literacy Frameworks: Analysis in Australian University Libraries. J Aust Libr Inf Assoc. 2024;73(4):485–504. doi: 10.1080/24750158.2024.2380541

[14] Shatila K, Aránega AY, Soga LR, Hernández-Lara AB. Digital literacy, digital accessibility, human capital, and entrepreneurial resilience: a case for dynamic business ecosystems. J Innov Knowl. 2025;10(3):100709. doi: 10.1016/j.jik.2025.100709

[15] Caroline A, Coun MJH, Gunawan A, Stoffers J. A systematic literature review on digital literacy, employability, and innovative work behavior: emphasizing the contextual approaches in HRM research. Front Psychol. 2025;15:1448555. doi: 10.3389/fpsyg.2024.1448555 39895978 PMC11783849

[16] Zhan Y, Yang S. Does internet use improve employment?--Empirical evidence from China. PLoS One. 2024;19(4):e0301465. doi: 10.1371/journal.pone.0301465 38626033 PMC11020373

[17] Cai Y, Liu S, Zhang X. The digital economy and job quality: Facilitator or inhibitor? --Evidence from micro-individuals. Heliyon. 2024;10(4):e26536. doi: 10.1016/j.heliyon.2024.e26536 38404788 PMC10884905

[18] Luan D, Xu B, Chen L. Financial pension security, digital literacy, and the well-being perception of the elderly population. Finance Res Lett. 2025;107857. doi: 10.1016/j.frl.2025.107857

[19] Wang J. Can digital literacy improve income mobility? Evidence from China. Telecommun Policy. 2025;49(6):102960. doi: 10.1016/j.telpol.2025.102960

[20] Chen Z, Cui R, Tang C, Wang Z. Can digital literacy improve individuals’ incomes and narrow the income gap?. Technol Forecast Soc Change. 2024;203:123332. doi: 10.1016/j.techfore.2024.123332

[21] Bejaković P, Mrnjavac Ž. The importance of digital literacy on the labour market. ER. 2020;42(4):921–32. doi: 10.1108/er-07-2019-0274

[22] Reddy P, Sharma B, Chaudhary K. Digital literacy: A review of literature. Int J Technoethics. 2020;11(2):65–94. doi: 10.4018/IJT.20200701.oa1

[23] Biberman G, Baril GL, Kopelman RE. Comparison of Return-on-Effort and Conventional Expectancy Theory Predictions of Work Effort and Job Performance: Results from Three Field Studies. J Psychol. 1986;120(3):229–37. doi: 10.1080/00223980.1986.10545249

[24] Tinmaz H, Lee Y-T, Fanea-Ivanovici M, Baber H. A systematic review on digital literacy. Smart Learn Environ. 2022;9(1):21. doi: 10.1186/s40561-022-00204-y 40478098 PMC9175160

[25] Li S. Digital literacy and farmers’ non-farm employment-an empirical study based on CFPS. Sustain Futures. 2025;10:100999. doi: 10.1016/j.sftr.2025.100999

[26] Winters JV. Human capital externalities and employment differences across metropolitan areas of the USA. J Econ Geogr. 2013;13(5):799–822. doi: 10.1093/jeg/lbs046

[27] Pastore F. Productivity, investment in human capital and the challenge of youth employment. Taylor & Francis; 2013.

[28] Ali A, Yang Q, Ali A, Ali Z, Noman M. How and when AI-driven capabilities foster organizational sustainability: A moderated mediation analysis of digital literacy and green HRM. Int J Inf Manage. 2025;85:102956. doi: 10.1016/j.ijinfomgt.2025.102956

[29] Shiyuan Y, Jinxiu Y, Jingfei X, Yuling Z, Longhua Y, Houjian L, et al. Impact of human capital and social capital on employability of Chinese college students under COVID-19 epidemic-Joint moderating effects of perception reduction of employment opportunities and future career clarity. Front Psychol. 2022;13:1046952. doi: 10.3389/fpsyg.2022.1046952 36605287 PMC9809468

[30] Kontu K, Vimpari J, Penttinen P, Junnila S. Individual ground source heat pumps: Can district heating compete with real estate owners’ return expectations? Sustainable Cities Soc. 2020;53:101982. doi: 10.1016/j.scs.2019.101982

[31] Vithana K, Jayasekera R, Choudhry T, Baruch Y. Human Capital resource as cost or investment: A market-based analysis. Int J Hum Resour Manag. 2023;34(6):1213–45. doi: 10.1080/09585192.2021.1986106

[32] Becker GS. Investment in Human Capital: A Theoretical Analysis. J Polit Econ. 1962;70(5, Part 2):9–49. doi: 10.1086/258724

[33] Riad A, Bahavan B, Koščik M. From institutional trust to digital literacy: Socioeconomic and political determinants of COVID-19 vaccine hesitancy among Czech adults based on a national panel survey. Hum Vaccin Immunother. 2025;21(1):2533639. doi: 10.1080/21645515.2025.2533639 40665470 PMC12269670

[34] Zhao R, Xu J, Zhao Y, Feng Y. Resource allocation pattern to green technology innovation efficiency: Synergy between environmental resource orchestration and firms’ digital capabilities. J Innov Knowl. 2025;10(4):100760. doi: 10.1016/j.jik.2025.100760

[35] Wu B, Guo Y, Chen Z, Wang L. Do agricultural productive services impact the carbon emissions of the planting industry in China: Promotion or inhibition? Sustainability. 2024;16(16):6850. doi: 10.3390/su16166850

[36] Cheng X, Chen J, Jiang S, Dai Y, Shuai C, Li W, et al. The impact of rural land consolidation on household poverty alleviation: The moderating effects of human capital endowment. Land Use Policy. 2021;109:105692. doi: 10.1016/j.landusepol.2021.105692

[37] Zhao J, Li W. The Impact of Digital Finance on the Urban–Rural Income Gap in China: The Mediating Role of Employment Structural Transformation. Sustainability. 2024;16(19):8365. doi: 10.3390/su16198365

[38] Du Z-Y, Wang Q. Digital infrastructure and innovation: Digital divide or digital dividend? J Innov Knowl. 2024;9(3):100542. doi: 10.1016/j.jik.2024.100542

[39] Ma J, Wang J, Szmedra P. Economic Efficiency and Its Influencing Factors on Urban Agglomeration—An Analysis Based on China’s Top 10 Urban Agglomerations. Sustainability. 2019;11(19):5380. doi: 10.3390/su11195380

[40] He Y, Zhou G, Tang C, Fan S, Guo X. The spatial organization pattern of urban-rural integration in urban agglomerations in China: An agglomeration-diffusion analysis of the population and firms. Habitat Int. 2019;87:54–65. doi: 10.1016/j.habitatint.2019.04.003

[41] Chen H, Chen C, Li Y, Qin L, Qin M. How Internet usage contributes to livelihood resilience of migrant peasant workers? Evidence from China. J Rural Stud. 2022;96:112–20. doi: 10.1016/j.jrurstud.2022.09.028

[42] Cetindamar D, Abedin B, Shirahada K. The Role of Employees in Digital Transformation: A Preliminary Study on How Employees’ Digital Literacy Impacts Use of Digital Technologies. IEEE Trans Eng Manage. 2021;71:7837–48. doi: 10.1109/tem.2021.3087724

[43] Wang X, Huang Y, Zhao Y, Feng J. Digital Revolution and Employment Choice of Rural Labor Force: Evidence from the Perspective of Digital Skills. Agriculture. 2023;13(6):1260. doi: 10.3390/agriculture13061260

[44] Dettling LJ. Broadband in the labor market: The impact of residential high-speed internet on married women’s labor force participation. ILR Rev. 2017;70(2):451–82. doi: 10.1177/0019793916644721

[45] Vroom V, Porter L, Lawler E. Expectancy theories. Organizational behavior 1. Routledge; 2015. p. 94–113.

[46] Seiger CP, Wiese BS. Social Support, Unfulfilled Expectations, and Affective Well-being on Return to Employment. J Marriage Family. 2011;73(2):446–58. doi: 10.1111/j.1741-3737.2010.00817.x

[47] Heymans MW, de Vet HCW, Knol DL, Bongers PM, Koes BW, van Mechelen W. Workers’ beliefs and expectations affect return to work over 12 months. J Occup Rehabil. 2006;16(4):685–95. doi: 10.1007/s10926-006-9058-8 17063403

[48] Ali A, Raza AA, Qazi IA. Validated digital literacy measures for populations with low levels of internet experiences. Dev Eng. 2023;8:100107. doi: 10.1016/j.deveng.2023.100107

[49] Fong MWL. Digital Divide Between Urban and Rural Regions in China. E J Info Sys Dev Countries. 2009;36(1):1–12. doi: 10.1002/j.1681-4835.2009.tb00253.x

[50] Lythreatis S, Singh SK, El-Kassar A-N. The digital divide: A review and future research agenda. Technol Forecasting Social Change. 2022;175:121359. doi: 10.1016/j.techfore.2021.121359

[51] de Clercq M, D’Haese M, Buysse J. Economic growth and broadband access: The European urban-rural digital divide. Telecommun Policy. 2023;47(6):102579. doi: 10.1016/j.telpol.2023.102579

[52] Thomä J. An urban-rural divide (or not?): Small firm location and the use of digital technologies. J Rural Stud. 2023;97:214–23. doi: 10.1016/j.jrurstud.2022.12.020

[53] Salemink K, Strijker D, Bosworth G. Rural development in the digital age: A systematic literature review on unequal ICT availability, adoption, and use in rural areas. J Rural Stud. 2017;54:360–71. doi: 10.1016/j.jrurstud.2015.09.001

