# Supplementary material for: The relationship between digital literacy and employment quality: Evidence from a Chinese Household Survey
Source: PLoS One. 2026 Sep 22;21(9):e0358194. doi: 10.1371/journal.pone.0358194 (PMC13596816; doi:10.1371/journal.pone.0358194)
Supplement: Supplementary file 2 [file pone.0358194.s004.docx]

**Response to Reviewers**

**Manuscript ID:** [PONE-D-25-44300R1]

**Title:** The Relationship Between Digital Literacy and Employment Quality: Evidence from a Chinese Household Survey

**Dear Editors and Reviewers,**

We express our sincere gratitude to the editor and reviewers for dedicating their valuable time and providing insightful comments on this manuscript. Following the second round of external review, we believe these detailed suggestions will significantly enhance the quality and clarity of our paper. We have carefully considered all feedback and implemented corresponding revisions to the manuscript. Specific responses to each comment are provided below. In the “revised manuscript with tracked changes,” all modifications have been highlighted in red for your convenience.

**Response to Editor Comments**

**Requirement 1:** I kindly request that the authors have the entire manuscript carefully reviewed and edited by a native English speaker to address language issues, grammatical errors, and overly technical jargon.

**Response:** Thank you very much for your careful and constructive feedback on our manuscript (PONE-D-25-44300R1). We appreciate the time and effort you and the reviewers have invested in evaluating our work. We also sincerely apologize for the language issues and the use of overly technical jargon that may have hindered the clarity and readability of our paper.

In response to your request that the entire manuscript be carefully reviewed and edited by a native English speaker, we have taken the following measures:

**1. Professional Language Editing** – We engaged a native English‑speaking editor with extensive experience in social science and economics manuscripts to perform a comprehensive language review. The editor scrutinized every section—from the abstract and introduction to the methodology, results, discussion, and conclusions—correcting grammatical errors, improving sentence structure, and ensuring consistent and precise use of terminology.

**2. Reduction of Jargon** – In addition to grammatical corrections, we simplified and clarified technical expressions that might be overly specialized. For example:

In the **Methodology** section, we replaced phrases such as “entropy method” with “entropy weighting method” and provided a plain‑language explanation of the calculation steps to make the procedure more accessible.

In the **Theoretical Analysis** section, we rephrased complex theoretical terms (e.g., “diminishing marginal benefit of digital literacy”) with more straightforward expressions while retaining academic rigor.

The **Discussion and Conclusions** have been rewritten to avoid unnecessary acronyms and to clearly state policy implications in plain English, ensuring that readers from diverse backgrounds can easily grasp the practical takeaways.

**3. Consistency and Flow** – The editor also checked for coherence across paragraphs, improved transition sentences, and harmonized the use of key terms (e.g., “digital literacy,” “employment quality”) throughout the manuscript to avoid ambiguity.

All changes are marked in the revised manuscript using the “Track Changes” function. We believe that the revised text now meets the high linguistic standards expected by PLOS ONE and that the content is both scientifically precise and broadly understandable.

We are grateful for your guidance and hope that the revised version is now suitable for publication. Should you or the reviewers have any further concerns, we are happy to address them promptly.

**Response to Reviewer 1**

**Minor Comment 1:** Something is wrong with the mathematical symbols in Equation (1) – I see only empty boxes in that equation.

**Response:** Thank you for your careful reading and for bringing to our attention the display issue with the mathematical symbols in Equation (1) of our manuscript. We sincerely apologize for the inconvenience this has caused.

To address this issue, we have thoroughly rewritten Equation (1) in the revised manuscript using a more robust and widely compatible format, and we have also submitted a PDF version of the manuscript. For your convenience, the corrected equation is shown below as an image:

We have verified that the revised equation displays properly in the PDF generated from the updated manuscript. We have also checked the other equations (2)–(7) to ensure they are free of similar issues.

We are grateful for your vigilance, and we hope that this revision fully addresses your concern. Should you encounter any further technical difficulties, please do not hesitate to let us know, and we will be happy to provide alternative formats if needed. Thank you again for your valuable guidance.

**Minor Comment 2:** A similar comment concerns line 452-457: empty boxes instead of mathematical symbols.

**Response:** We sincerely thank you for your careful review and for pointing out the display issue with mathematical symbols in lines 452–457 of our original manuscript. We apologize for the inconvenience caused by these rendering problems.

To ensure complete clarity and correct display, we have thoroughly revised all equations in the manuscript. Specifically:

In Equations (1) - (4), *X_ij_* denotes the dimensionless normalized value of the *j*th evaluation index for the *i*th research unit; *n* represents the number of research units, and *m* represents the number of indicators; *d_j_* is the entropy value of the *j*th indicator; *W_j_* is the weight of the *j*th indicator; *S_i_* is the comprehensive evaluation index of the *i*th research unit.

We have now verified that the revised manuscript, when compiled into PDF, displays all mathematical symbols correctly—with no empty boxes or missing characters. The updated equations are clearly readable and consistent with standard academic formatting.

We are grateful for your vigilance; this correction has improved the technical quality of our paper. Should you have any further concerns, we would be happy to provide a separate plain‑text version of the equations or supplementary files as needed. Thank you again for your valuable time and suggestions.

**Minor Comment 3:** Line 467-468: as the author/authors uses/use OLS as their research method so what they investigate is the relation between independent variables and the dependent variable and not causality. Therefore, in my view, the statement “denotes a set of control variables that may influence the employment quality of individual labor force” is way too strong and should be rewritten so as to indicate that the author/authors investigates/investigate a relation between the independent variables and dependent variable.

**Response:** We are very grateful for your careful and precise reading of our manuscript. Your observation regarding the use of causal language in the model specification section is both insightful and methodologically important. We fully agree that ordinary least squares (OLS) regression, as a correlational method, does not by itself establish causality, and our wording should accurately reflect the nature of our empirical strategy.

In the original text (lines 467–468), we wrote: “*C_i_* denotes a set of control variables that may influence the employment quality of individual labor force *i*.”

As you rightly pointed out, this phrasing is too strong and implies a causal claim. In the revised manuscript, we have rewritten this sentence to clearly indicate that we are examining associations or relationships, not causal effects. The revised wording now reads (lines 485-486 in the revised manuscript):

“*C_i_* denotes a set of control variables that are associated with the employment quality of individual labor force *i*.”

We have made this change consistently throughout the manuscript wherever similar causal language appeared, including in the abstract, the theoretical hypothesis statements (which we have rephrased as “is positively associated with” rather than “promotes” or “enhances”), and in the discussion section where we now use terms such as “correlate,” “relationship,” and “association” instead of “impact” or “effect” when referring to the OLS results.

We believe that this revision aligns the language of our paper with the methodological rigor of our analytical approach, and we thank you for helping us improve the precision and clarity of our manuscript.

**Response to Reviewer 2**

**Comment 1: Causal Interpretation Remains Problematic.**

Although the authors have revised some language from "influence" to "association," the manuscript still occasionally implies causal relationships, particularly in the theoretical framework, discussion, and policy recommendations. The study relies primarily on cross-sectional observational data from CFPS 2022 and OLS regressions. Such a design does not permit strong causal inference. Even with the instrumental variable approach, the manuscript should more thoroughly justify instrument validity and acknowledge remaining limitations.

**Recommendation:**

Consistently use associative language throughout the manuscript.

Explicitly state in the limitations section that causality cannot be established.

Temper policy recommendations that assume causal effects.

**Response:** We sincerely thank you for your careful and rigorous evaluation of our revised manuscript. Your observation regarding the remaining causal language is both constructive and methodologically critical. We fully agree that our cross‑sectional OLS design, even with instrumental variable (IV) estimation, does not support strong causal claims, and that the manuscript should consistently reflect this limitation.

In response, we have undertaken a thorough revision of the entire manuscript to systematically replace causal language with associative terminology. Below we detail the specific actions taken, addressing each of your recommendations.

**1. Consistent use of associative language throughout the manuscript.**

We have carefully reviewed and revised every section to ensure that language reflects correlation or association rather than causation. Key modifications include:

**Title:**

**Original:** “Research on the Impact and Mechanism of Digital Literacy on Employment Quality: Evidence from the China Family Panel Studies”

**Revised:** “The Relationship Between Digital Literacy and Employment Quality: Evidence from a Chinese Household Survey”

**Abstract:** Changed “determinant” to “correlate”; replaced “promoting” with “associated with”; revised “enhance” to “correspond to”.

**Introduction and Theoretical Framework:** All hypotheses have been reworded. For example:

**Original:** “Digital literacy has a significant positive impact on labor employment quality.”

**Revised:** “Digital literacy is significantly positively associated with labor employment quality.”

Similarly, H2 and H3 now state “positively moderates the association” and “negatively moderates the association,” rather than “impact.”

**Methodology:** The model specification now states that we examine the “relationship” or “association” between variables, not their “effect” or “influence.” For instance, the sentence describing C_i_ (control variables) has been changed from “may influence” to “are associated with” (as previously adjusted per your earlier suggestion).

**Results and Discussion:** We replaced all instances of “effect” (when referring to OLS coefficients) with “association” or “coefficient.” For example, “the positive effect” becomes “the positive association”; “the impact of digital literacy” becomes “the relationship between digital literacy and employment quality.”

**Conclusions and Policy Implications:** We have significantly tempered the language. We now frame our policy recommendations as preliminary implications derived from observed associations, rather than established causal effects. We explicitly state that these suggestions should be considered alongside other evidence and are not definitive prescriptions.

All changes are marked in the revised manuscript using track changes. Thank you again for your valuable time and suggestions.

**2. Explicitly state in the limitations section that causality cannot be established.**

We have expanded the Limitations subsection (formerly in Discussion, now clearly labeled) to explicitly address the causal inference issue. The added text reads (lines 901-911 in the revised manuscript):

Fourthly, our empirical strategy primarily relies on cross‑sectional observational data from the 2022 CFPS and OLS regressions. While we have employed propensity score matching and instrumental variable estimation to mitigate endogeneity concerns, these methods do not fully eliminate the possibility of unobserved confounding or reverse causality. The IV approach, in particular, rests on the untestable exclusion restriction assumption, which we have justified on theoretical grounds but cannot verify directly. Therefore, we interpret all our findings as associative evidence rather than causal effects. Readers and policymakers should exercise caution when attributing changes in employment quality to improvements in digital literacy based solely on this study.

This addition is placed immediately after the discussion of IV limitations, ensuring that the overall conclusion clearly acknowledges the correlational nature of our evidence.

**3. Temper policy recommendations that assume causal effects.**

We have revised the policy recommendations section to reflect a more cautious tone. Instead of stating that policies “will” improve employment quality, we now emphasize that our findings suggest or indicate potential directions that merit consideration, and that pilot testing and further evaluation are necessary before large‑scale implementation.

For example:

**Original:** “Policies should prioritize … digital skills compensation training programs … to address their traditional skill shortages.”

Revised: “Our findings suggest that policies could prioritize … digital skills compensation training programs … as a means to potentially address traditional skill shortages, though such interventions should be rigorously evaluated.”

Similarly, in the regional strategies, we replaced definitive statements like “policies should expedite” with “policymakers may consider expediting,” and we added a caveat that local contexts and complementary investments are crucial for success.

For your convenience, we hereby present the revised policy recommendations below, with the changes highlighted in red (lines 934-1004 in the revised manuscript):

Given the correlational nature of our empirical evidence, the following policy implications should be interpreted as preliminary and tentative. They are intended to stimulate discussion and inform hypothesis generation for future evaluation, rather than to serve as definitive causal prescriptions. Any actual policy design should be preceded by rigorous pilot testing and contextual feasibility assessments.

Based on these associative findings, we offer the following tentative policy considerations:

Firstly, it may be worthwhile to consider formulating precise and incentive-compatible intervention programs tailored to different human capital groups. Considering that the positive correlation between digital literacy and employment quality is more pronounced among low-human-capital (low educational attainment) workers, policymakers could give priority to and concentrate on this group. It may be advisable to design and implement "digital skills compensation" training programs that closely align basic and advanced digital skills with specific and localized non-agricultural job requirements. Such programs could help address their traditional skill shortages and potentially maximize the utility of digital literacy as a "tool for crossing the employment threshold." Simultaneously, to potentially improve training conversion efficiency, "incentive modules" could be systematically incorporated into the courses. This might involve presenting real-life cases of learners with similar backgrounds who have successfully enhanced their employment quality through improving digital skills, offering clear career development path guidance, and exploring linkages between training certificates and employment recommendations, small-scale entrepreneurship support, and other incentive policies—thus possibly increasing participants' expected returns and stimulating their intrinsic motivation to learn and apply digital skills.

Secondly, policymakers may consider implementing a differentiated strategy to enhance digital literacy and promote industrial development in a coordinated manner. In the western regions, considering that the digital infrastructure is in a catch-up stage and the marginal returns of digital skills are high, two aspects deserve policy attention: First, expediting the popularization of broadband networks and public digital service platforms and lowering the access threshold. Second, vigorously launching the "Basic Digital Skills Popularization Campaign," focusing on training practical skills related to the digital transformation of local characteristic industries, so as to potentially improve the competitiveness of workers in the local labor market. Meanwhile, in the developed eastern regions, the policy focus might transition from "popularization" to "deepening" and "certification." Promoting the establishment of an advanced digital skills certification system, encouraging enterprises, educational institutions, and training organizations to offer relevant courses, and moderately linking them to salary systems and professional title evaluations could help meet the demand for high-level digital talents in industrial upgrading and assist workers in obtaining potential returns. Additionally, considering the characteristics of industrial structure transformation in the central regions, policies could emphasize "demand-driven" approaches. In combination with local manufacturing upgrade plans and service industry development initiatives, tailoring digital skills training content—such as intelligent equipment operation, industrial internet applications, and digital cultural and creative industries—may better align skill supply with future industrial demands and facilitate the realization of digital skill value.

Thirdly, it may be beneficial to utilize the radiation effect of urban agglomerations to construct cross-regional hubs for digital talent development and resource sharing. Fully capitalizing on the advantages of major urban agglomerations—such as the Beijing-Tianjin-Hebei region, the Yangtze River Delta, and the Pearl River Delta—as digital resource hubs, supporting core cities in these urban agglomerations to establish "Digital Talent Training and Exchange Centers" and develop high-quality, standardized training courses and online learning platforms could be considered. Through a "pairing assistance" mechanism, encouraging these centers to export course resources, teaching personnel, and training opportunities to non-agglomeration areas in surrounding regions may help narrow regional gaps. Simultaneously, promoting the mutual recognition of digital skills certifications and facilitating talent mobility within and among urban agglomerations could ensure that returns on digital skills are not confined to local areas but can be realized through broader job markets, thereby potentially amplifying the positive implications of improving digital literacy.

**Comment 2: Instrumental Variable Strategy Requires Stronger Justification.**

The response notes that Models (6) and (7) correspond to first- and second-stage regressions. However, it remains unclear whether the proposed instrument satisfies:

Relevance condition

Exclusion restriction

The manuscript should provide a stronger theoretical justification for why the instrument affects employment quality only through digital literacy.

**Recommendation:**

Discuss instrument validity in greater detail.

Report relevant diagnostics (e.g., weak instrument tests, first-stage F-statistic, over-identification tests if applicable).

Include a dedicated subsection discussing IV assumptions.

**Response:** We sincerely thank you for your rigorous and constructive feedback on our instrumental variable (IV) strategy. You are absolutely correct that the validity of the IV approach hinges critically on both the relevance condition and the exclusion restriction, and that our manuscript needed stronger theoretical justification and more comprehensive diagnostic reporting.

In response to your recommendation, we have undertaken the following revisions:

**1. Discuss instrument validity in greater detail.**

In the revised manuscript, we have added a dedicated paragraph to provide a more in-depth discussion of the validity of the instrumental variables (lines 601-632 in the revised manuscript). For your convenience, the updated content on the assessment of instrumental variable validity is presented below.

The exclusion restriction requires that the instrument has no direct effect on employment quality other than through digital literacy. We argue that this condition is plausibly satisfied for three interconnected reasons.

First, the perceived value of the Internet is strongly correlated with the endogenous explanatory variable, digital literacy. Individuals who regard the Internet as highly important in their daily routines are more likely to engage actively with digital devices and online platforms, thereby accumulating practical digital skills and knowledge through routine use, self-directed learning, or social interaction. This positive association between perceived importance and actual digital competence has been well-documented in prior literature [4, 12]. Second, the perceived value of the Internet affects the quality of employment solely by influencing digital literacy, and not through any other direct means. An individual's general attitude toward the Internet's importance in daily life is a broad, non‑specific psychological predisposition, rather than a targeted evaluation of its role in career advancement. Such a general perception is unlikely to directly determine specific labor market outcomes such as wage levels, the existence of a labor contract, social security coverage, or promotion opportunities—these are predominantly shaped by structural market factors, individual professional skills, work experience, and industry and occupation‑specific characteristics [18]. Third, we emphasize that this variable measures "importance in daily life" rather than specifically "importance in career development," which conceptually differentiates it from direct labor market drivers. While a person who values the Internet highly may also be more motivated to improve digital literacy, that same perception does not, in itself, influence employers' hiring, contracting, or promotion decisions, nor does it affect job characteristics such as industry or occupational rank, except through the digital skills it fosters, thereby satisfying the exclusion restriction required for a valid instrument [20]. Specifically, the instrumental variable should be correlated with the endogenous explanatory variable (digital literacy) and uncorrelated with the error term.

**2. Report relevant diagnostics (e.g., weak instrument tests, first-stage F-statistic, over-identification tests if applicable).**

We wholeheartedly agree with your perspective. To this end, we have incorporated the F-statistics and Lagrange Multiplier (LM) test statistics for the first stage into Table 6 of the revised manuscript and provided supplementary explanations of the relevant diagnostic results in the “Results and Analysis” section. For your convenience, the newly added diagnostic content in Table 6 and the associated explanatory notes are presented below (lines 633-649 in the revised manuscript).

**Table 6. Robustness test and instrumental variable test.**

| Variable | Replace the Explained Variable | Change the Sample Size | Winsorization | Digital Literacy | Employment Quality |
| --- | --- | --- | --- | --- | --- |
|  | Model (3) | Model (4) | Model (5) | Model (6) | Model (7) |
| Digital literacy | 1.040*** | 0.116*** | 0.092*** |  | 0.318*** |
|  | (0.043) | (0.012) | (0.010) |  | (0.040) |
| Instrumental variable |  |  |  | 0.056*** |  |
|  |  |  |  | (0.002) |  |
| Control variable | Yes | Yes | Yes | Yes | Yes |
| Constant | -1.853*** | 0.934*** | 0.950*** | 0.034 | 0.947*** |
|  | (0.312) | (0.081) | (0.074) | (0.078) | (0.078) |
| F-value |  |  |  | 597.53 |  |
| LM-value |  |  |  | 488.28*** |  |
| N | 7,625 | 6,862 | 7,444 | 7,625 | 7,625 |
| R^2^ | 0.231 | 0.047 | 0.037 |  |  |

Note: Model (6) showcases the first-stage regression results of the instrumental variable approach, and Model (7) showcases the corresponding second-stage results.

The IV estimation results are reported in Models (6) and (7) of Table 6. The findings indicate that the instrumental variable is significantly positively correlated with digital literacy. Moreover, the first-stage F-statistic is 597.53, which exceeds the critical value of 16.38 at the 10% significance level, thereby effectively eliminating the presence of weak instrumental variables[35]. The LM statistic is significant at the 1% significance level, indicating that the instrumental variable passes the test for underidentification. After correcting for endogeneity bias, digital literacy continues to exhibit a statistically significant positive association with employment quality in the labor market, and the absolute value of the coefficient increases. This further substantiates the findings of the benchmark regression.

While the exclusion restriction remains empirically untestable, we believe the theoretical reasoning provided above offers a credible basis for its plausibility. We also acknowledge this limitation explicitly in the Discussion section and call for future research using more exogenous instruments.

**3. Include a dedicated subsection discussing IV assumptions.**

We sincerely thank you for your recommendation to include a dedicated subsection discussing instrumental variable (IV) assumptions. We fully agree that a clearly delineated section focused on IV validity enhances the transparency and methodological rigor of our empirical strategy.

To this end, the “Endogenous Discussion” section is divided into two subsections: Propensity Score Matching (PSM) and Instrumental Variable (IV) Test. The former aims to address potential endogeneity arising from self-selection bias in digital literacy, while the latter rigorously examines the relevance condition and exclusion restriction, and presents detailed diagnostic results (The newly added subsection is located in lines 590 - 649 of the revised manuscript).

We are confident that this dedicated subsection fully addresses your recommendation and substantially enhances the methodological transparency of our manuscript. We are deeply grateful for your careful and constructive guidance.

**Comment 3: Construction of Composite Indices Needs More Transparency.**

Both digital literacy and employment quality are constructed using entropy-weighted indices. While this approach is reasonable, readers need additional information regarding:

Indicator selection rationale

Weighting procedures

Robustness to alternative weighting schemes

Reliability and validity assessments

**Recommendation:**

Supplementary tables showing indicator weights, or Sensitivity analyses using alternative index construction methods.

**Response:** We sincerely thank you for your thoughtful and constructive suggestion to enhance the transparency of our composite index construction. We fully agree that readers need clear information regarding indicator selection rationale, weighting procedures, robustness to alternative schemes, and reliability and validity assessments. In response, we have substantially revised the relevant sections of the manuscript and added supplementary materials to address each of your recommendations.

**1. Indicator Selection Rationale.**

We have expanded the rationale for indicator selection in both the “**Explained Variable**” and “**Core Explanatory Variable**” subsections. The revised text now includes:

**For Employment Quality (added text):**

“The selection of indicators for employment quality is grounded in the International Labour Organization’s ‘Decent Work’ framework [5], which emphasizes both objective conditions (remuneration, security, and career progression) and subjective perceptions (satisfaction with income, job security, work environment, working hours, and promotion prospects). We include labor remuneration indicators (wage, bonus, and fringe benefits) to capture economic returns; employment security indicators (labor contract, pension insurance, health insurance, and housing provident fund) to reflect job stability and social protection; and career prospects to capture upward mobility. Subjective satisfaction indicators are included to capture workers’ perceived well-being, which is increasingly recognized as a core dimension of employment quality [5]. All indicators are aligned with the multidimensional nature of employment quality and are commonly used in the existing literature [16]. Moreover, to address the limitations associated with relying on single indicators, this study employs the Entropy Method to construct a comprehensive employment quality index. The detailed evaluation framework and the weight of indicators are presented in Table 1 (lines 346-363 in the revised manuscript).”

**For Digital Literacy (added text):**

“The selection of indicators for digital literacy is guided by established digital literacy frameworks, including UNESCO’s Global Digital Literacy Framework [11] and the European Union’s DigComp framework, which conceptualize digital literacy as a multidimensional construct encompassing both technical skills and cognitive awareness. The ‘digital ability’ dimension captures behavioral engagement with digital tools across four domains—learning, shopping, entertainment, and social activities—which reflect the breadth and diversity of digital tool utilization. Sustained and diverse engagement with digital tools is a fundamental prerequisite for and behavioral manifestation of skill acquisition and development [18]. The ‘digital cognition’ dimension captures subjective perceived importance of the Internet across the same domains, reflecting the motivational and evaluative dimension of digital literacy that influences the depth and purposefulness of technology use. This dual-dimensional approach—combining observed behavioral engagement with subjective cognitive appraisal—provides a more holistic approximation of an individual’s digital literacy than usage alone, acknowledging that in real-world contexts, skill is often exercised and demonstrated through purposeful use [20]. The detailed evaluation indicator system and indicator weights are presented in Table 2.

It is worth noting that the selection of indicators for “digital capabilities”—namely online learning, online shopping, online entertainment, and online social activities—requires conceptual justification. While these items reflect usage frequency or participation rather than directly assessing proficiency levels, they serve as valid proxy measures within the constraints of large-scale social survey data like the CFPS. Grounded in digital literacy theory, sustained and diverse engagement with digital tools is a fundamental prerequisite for and a behavioral manifestation of skill acquisition and development [21, 22]. Furthermore, the inclusion of “digital cognition” (subjective perceived importance across the same domains) complements these behavioral measures by capturing the motivational and evaluative dimension of digital literacy, which influences the depth and purposefulness of technology use. This combined approach of observed behavioral engagement and subjective cognitive appraisal provides a more holistic approximation of an individual’s digital literacy than usage alone, acknowledging that in real-world contexts, skill is often exercised and demonstrated through purposeful use (lines 367-404 in the revised manuscript).”

**2. Weighting Procedures.**

We have expanded the “Comprehensive Index Construction Method” subsection to provide a detailed step-by-step explanation of the entropy weighting procedure. The revised text now reads (lines 463-475 in the revised manuscript):

Step 1: Dimensional normalization processing. To eliminate scale effects, we normalize each indicator using min – max normalization:

$$X_{ij}=\left\{ \begin{aligned} \frac{x_{ij}-min\left( x_{ij} \right)}{max\left( x_{ij} \right)-min\left( x_{ij} \right)}, if x_{ij}\text{ }\text{is}\text{ }\text{a}\text{ }\text{positive}\text{ }\text{indicator} \\ \frac{max\left( x_{ij} \right)-x_{ij}}{max\left( x_{ij} \right)-min\left( x_{ij} \right)}, if x_{ij}\text{ }\text{is}\text{ }\text{a}\text{ }\text{negative}\text{ }\text{indicator} \end{aligned} \right.$$

(1)

Step 2: Calculation of entropy value. For each indicator *j*, we calculate the entropy value *d_j_* as:

$$d_{j}=\sum_{i=1}^{n} \left\{ \frac{X_{ij}}{\int_{n=1}^{n} X_{ij}}\times\ln\frac{X_{ij}}{\int_{n=1}^{n} X_{ij}} \right\}$$

(2)

Step 3: Determination of indicator weights. The weight *W_j_* for indicator *j* is calculated as:

$$W_{j}=\frac{1-d_{i}}{\sum_{j=1}^{m} \left( 1-d_{i} \right)}$$

(3)

Step 4: Calculation of the comprehensive evaluation index. The final composite index *S_i_* for each individual *i* is calculated as:

$$S_{i}=\sum_{j=1}^{m} \left( W_{j}\times X_{ij} \right)$$

(4)

In Equations (1) - (4), *X_ij_* denotes the dimensionless normalized value of the *j*th evaluation index for the *i*th research unit; *n* represents the number of research units, and *m* represents the number of indicators; *d_j_* is the entropy value of the *j*th indicator; *W_j_* is the weight of the *j*th indicator; *S_i_* is the comprehensive evaluation index of the *i*th research unit.

**3. Supplementary tables showing indicator weights.**

We sincerely thank you for your thoughtful and constructive suggestion to enhance the transparency of our composite index construction. We have supplemented the weights of all indicators in Tables 1 and 2. The supplemented weights are presented below for your review.

**Table 1. Employment quality evaluation system.**

| Dimension | Variable Description | | Variable Assignment | Indicator weights |
| --- | --- | --- | --- | --- |
| Objective level | Labor remuneration | Wage income | Log-transformed monthly after-tax wage (¥/month) | 0.253 |
|  |  | Bonus income | Log-transformed monthly after-tax bonus (¥/month) | 0.228 |
|  |  | Fringe benefits income | Log-transformed monthly after-tax fringe benefits amount (¥/month) | 0.131 |
|  | Employment security | Labor contract | 1 = Yes; 0 = No | 0.049 |
|  |  | Pension insurance | 1 = Yes; 0 = No | 0.079 |
|  |  | Health insurance | 1 = Yes; 0 = No | 0.078 |
|  |  | Housing provident fund | 1 = Yes; 0 = No | 0.006 |
|  | Career prospects | Career promotion | Promoted in the past 12 months: 1 = Yes; 0 = No | 0.069 |
| Subjective level | Subjective satisfaction evaluation | Income satisfaction | 1–5 (Very dissatisfied – Very satisfied) | 0.032 |
|  |  | Job security perception | 1–5 (Very dissatisfied – Very satisfied) | 0.018 |
|  |  | Work environment satisfaction | 1–5 (Very dissatisfied – Very satisfied) | 0.015 |
|  |  | Working hours satisfaction | 1–5 (Very dissatisfied – Very satisfied) | 0.020 |
|  |  | Promotion prospects satisfaction | 1–5 (Very dissatisfied – Very satisfied) | 0.021 |

**Table 2. Digital literacy evaluation system.**

| Dimension | Variable Description | Variable Assignment | Indicator weights |
| --- | --- | --- | --- |
| Digital ability | Online learning activities | 1 = Yes; 0 = No | 0.218 |
|  | Online shopping activities | 1 = Yes; 0 = No | 0.128 |
|  | Online entertainment activities | 1 = Yes; 0 = No | 0.364 |
|  | Online social activities | 1 = Yes; 0 = No | 0.073 |
| Digital Application Cognition | The importance of using the internet for working | 1–5 (Very unimportant – Very important) | 0.065 |
|  | The importance of using the internet for learning | 1–5 (Very unimportant – Very important) | 0.056 |
|  | The importance of using the internet for entertainment | 1–5 (Very unimportant – Very important) | 0.041 |
|  | The importance of using the internet for social activities | 1–5 (Very unimportant – Very important) | 0.054 |

**4. Robustness to alternative weighting schemes.**

We have added a new sensitivity analysis subsection in the “Robustness Test (lines 545-569 in the revised manuscript)” section to test the robustness of our findings to alternative weighting methods. The added text and supplementary table are as follows:

**Table 6. Robustness test and instrumental variable test.**

| Variable | Replace the Explained Variable | Change the Sample Size | Winsorization | Digital Literacy | Employment Quality |
| --- | --- | --- | --- | --- | --- |
|  | Model (3) | Model (4) | Model (5) | Model (6) | Model (7) |
| Digital literacy | 1.040*** | 0.116*** | 0.092*** |  | 0.318*** |
|  | (0.043) | (0.012) | (0.010) |  | (0.040) |
| Instrumental variable |  |  |  | 0.056*** |  |
|  |  |  |  | (0.002) |  |
| Control variable | Yes | Yes | Yes | Yes | Yes |
| Constant | -1.853*** | 0.934*** | 0.950*** | 0.034 | 0.947*** |
|  | (0.312) | (0.081) | (0.074) | (0.078) | (0.078) |
| F-value |  |  |  | 597.53 |  |
| LM-value |  |  |  | 488.28*** |  |
| N | 7,625 | 6,862 | 7,444 | 7,625 | 7,625 |
| R^2^ | 0.231 | 0.047 | 0.037 |  |  |

Note: Model (6) showcases the first-stage regression results of the instrumental variable approach, and Model (7) showcases the corresponding second-stage results.

To verify the robustness of the benchmark regression results, three alternative approaches were employed: First, to examine whether the benchmark regression results are sensitive to the choice of weighting method, we re-estimated the composite index of digital literacy and employment quality using factor analysis, where weights were derived from the factor loadings. Subsequently, we re-ran the benchmark regression analysis using these alternative indices (see Table 6, Model (3)). The results show that in Model (3), the coefficient on digital literacy remains positive and statistically significant at the 1% level. These findings demonstrate that the main conclusions of this paper are robust to different weighting schemes and are not driven by the use of entropy weights; Second, Model (4) is derived by adjusting the sample size—specifically, by randomly selecting 90% of the original sample and repeating the regression process; Third, Model (5) performs a regression analysis after removing the extreme values in the top and bottom 1% of the sample data to mitigate the impact of potential outliers. The corresponding results are presented in Models (3) to (5) of Table 6. The findings reveal that the correlation and statistical significance of digital literacy with employment quality remain largely unchanged across all robustness models. This consistency supports the robustness and reliability of the benchmark regression results, thereby confirming the validity of Hypothesis 1.

**Comment 4: Moderation Analysis Requires Further Interpretation.**

The moderation results are interesting but remain largely descriptive.

For example:

Why does human capital negatively moderate the relationship?

Could this result reflect ceiling effects?

Are there multicollinearity concerns between education and digital literacy?

**Recommendation:**

Provide deeper theoretical interpretation and discuss alternative explanations.

**Response:** We sincerely thank you for your insightful and constructive feedback on our moderation analysis. You are absolutely right that the moderation results, while interesting, require deeper theoretical interpretation and consideration of alternative explanations—particularly regarding the negative moderating effect of human capital, the possibility of ceiling effects, and potential multicollinearity concerns. We have carefully considered each of your points and have substantially revised the manuscript accordingly. In response, we have substantially revised the relevant sections of the manuscript and added supplementary materials to address each of your recommendations.

**1. Expanded Theoretical Reasoning for Negative Moderation (H3).**

We have substantially expanded the theoretical analysis for Hypothesis 3 (negative moderating effect of human capital) in the “Theoretical Analysis and Research Hypothesis” section. The revised text now reads (lines 242-284 in the revised manuscript):

Human capital theory suggests that an individual’s educational attainment, professional skills, and practical experience serve as fundamental pillars for career development and employment quality [26, 27]. Digital literacy is not only a key indicator of human capital accumulation, but its influence on the quality of labour employment is also significantly mediated by the individual’s existing level of human capital [15].

We propose a negative moderating effect of human capital based on three complementary mechanisms. First, diminishing marginal returns: workers with high levels of human capital—such as advanced education and extensive work experience—have already developed strong employability through formal educational investments. Their employment quality is largely determined by these established credentials, leaving relatively less room for digital literacy to contribute additional improvements. In contrast, workers with low human capital face more substantial skill deficits, and the acquisition of digital literacy can serve as a powerful ‘skill compensator,’ generating larger marginal improvements in their employment outcomes.

Second, substitution and opportunity cost effects: highly educated workers may have already acquired a broad set of competencies that partially overlap with or substitute for digital skills (e.g., analytical reasoning, problem‑solving, and information evaluation). Moreover, they may face higher opportunity costs in terms of time and effort required to acquire new digital skills, potentially reducing their relative motivation to invest in digital literacy enhancement [28]. Workers with lower educational attainment, by contrast, may perceive digital skills as a more novel and valuable addition to their skill set, leading to greater relative gains.

Third, cognitive and behavioral inertia: individuals with high human capital may exhibit over‑reliance on their existing competencies, leading to lower engagement with and adoption of emerging digital skill sets [28]. This behavioral pattern can attenuate the potential benefits of digital literacy for this group. Conversely, low‑human‑capital workers often face disadvantages in the labour market [29], making them more receptive to new skill acquisition as a strategy for improving their employment prospects.

In summary, while digital literacy contributes positively to employment quality overall, the marginal benefit tends to be larger for individuals with lower human capital—a pattern consistent with the ‘inclusive growth’ potential of digital upskilling policies.” Based on the above analysis, this paper formulates Research Hypothesis 3：

**Hypothesis 3:** Human capital negatively moderates the association of digital literacy on labor employment quality.

**2. Discussion of Ceiling Effects.**

We have added a dedicated discussion of ceiling effects in the “Discussion” section (lines 791-820 in the revised manuscript):

Secondly, the negative moderating effect of human capital (H3) reveals a phenomenon with significant policy implications. The significant negative interaction term indicates that the positive association between digital literacy and employment quality is more pronounced among workers with lower education levels and weaker traditional human capital. This challenges the simplistic assumption that the returns to digital skills increase linearly with educational attainment [22]. One plausible explanation for the negative moderating effect of human capital is the presence of ceiling effects. For workers with high educational attainment—particularly those holding college degrees or above—their employment quality may already be near the upper bound of the distribution due to their strong human capital endowments. In such cases, the marginal contribution of digital literacy to further employment quality gains is naturally constrained by the limited remaining room for improvement. This interpretation is consistent with the concept of diminishing marginal returns to skill investment, which predicts that additional skill accumulation yields progressively smaller benefits as individuals approach higher competence levels [48]. However, ceiling effects alone cannot fully explain the observed pattern. If the negative moderation were driven solely by ceiling effects, we would expect the relationship between digital literacy and employment quality to be weakest among the highest‑educated workers but not necessarily stronger among the lowest‑educated workers. Our results, however, show a particularly pronounced positive association among low‑human‑capital workers, suggesting that digital literacy actively compensates for skill deficits rather than merely operating in a context of low baseline employment quality. This ‘skill compensation’ mechanism is an important complement to the ceiling effect explanation and underscores the potential of digital upskilling to reduce employment inequality.

**3. Multicollinearity Concerns.**

We present the variance inflation factor (VIF) test results for all variables in the moderating model in Table 4. As shown in Table 4, the VIF values for digital literacy (1.310) and human capital (education) (1.450) are both well below the conventional threshold of 10, and the mean VIF across all variables is only 1.240. These results indicate that multicollinearity between education and digital literacy is not severe in our analysis.

**Table 4. VIF test results.**

| Variables | Digital literacy | Gender | Age | Expected returns | Mean value of VIF |
| --- | --- | --- | --- | --- | --- |
| VIF | 1.310 | 1.030 | 1.530 | 1.010 |  |
| Variables | Marital status | Political status | Household registration type | Human capital | 1.240 |
| VIF | 1.260 | 1.120 | 1.230 | 1.450 |  |

We are confident that these revisions provide the deeper theoretical interpretation and consideration of alternative explanations you recommended, and we believe they substantially enhance the conceptual depth and methodological rigor of our moderation analysis. We are deeply grateful for your careful and constructive guidance.
