# Supplementary material for: The relationship between digital literacy and employment quality: Evidence from a Chinese Household Survey
Source: PLoS One. 2026 Sep 22;21(9):e0358194. doi: 10.1371/journal.pone.0358194 (PMC13596816; doi:10.1371/journal.pone.0358194)
Supplement: Supplementary file 3 [file pone.0358194.s001.zip › Supporting Information/Ethics and Informed Consent Statement.pdf]

Ethics and Informed Consent Statement

Admissibility number: YZNU-U-20250610 **Application date:** 10 June 2025

**Applicant's Name**	Xiaoyun Wu	**Institution**	College of Economics and Management, Yuzhang Normal University
**Project Source**	Science and Technology Research Project of the Jiangxi Provincial Department of Education [Grant No. GJJ191219]；Jiangxi Social Science "14th Five-Year Plan" Fund Project [Grant No. 24JY37D]		
**Purpose of the application for an ethics letter**	The data used in this study is sourced from the public database China Family Panel Studies (CFPS), which did not involve animal or human clinical trials. In accordance with the ethical principles outlined in the Declaration of Helsinki, all participants provided informed consent prior to participating in the study, participant anonymity and confidentiality were assured, and participation was entirely voluntary. This application for an ethical letter was used for the publication of the paper and related academic research results needed. Meanwhile, this database is publicly available for free use in China. During the usage process, users are only required to state in the article that the data source of the research institute's data is CFPS, and there is no need to apply for other permission certificates.		
**Thesis title**	Research on the Impact and Mechanism of Digital Literacy on Labor Employment Quality		
**Research Purpose**	In the digital era, digital literacy has emerged as a critical determinant of employment quality within the labor market. Based on the 2022 China Family Panel Studies (CFPS) database, this study empirically examines the impact of digital literacy on the quality of labor employment and its mechanism, using methods such as Ordinary least squares (OLS) regressions and moderating effect models, and proposes targeted intervention policies.		

Review Comments

1. The research is not part of the study of ethics.
2. The questionnaire was conducted anonymously and all participants were fully informed about the reasons for conducting the survey and the use of the relevant data. No personally identifiable information was collected in the course of the survey, and there was no conflict of interest or potential risk to rights holders.

College of Economics and Management, Yuzhang Normal University

Date: 2025.06.15

豫章师范学院 经济与管理学院
