# Supplementary material for: The relationship between digital literacy and employment quality: Evidence from a Chinese Household Survey
Source: PLoS One. 2026 Sep 22;21(9):e0358194. doi: 10.1371/journal.pone.0358194 (PMC13596816; doi:10.1371/journal.pone.0358194)
Supplement: Peer review history

# Peer review: Decision Letter 0

**DOI:** 10.1371/journal.pone.0358194.r001

**Authors:** Md. Rabiul Awal

16 Dec 2025

PONE-D-25-44300

How Can the Digital Era Facilitate the Sustainable Development of the Labor Market? A Microscopic Exploration of Digital Literacy and Employment Quality

PLOS One?

Dear Dr. Wu,

plosone@plos.org. When you're ready to submit your revision, log on to https://www.editorialmanager.com/pone/ and select the 'Submissions Needing Revision' folder to locate your manuscript file.

- A rebuttal letter that responds to each point raised by the academic editor and reviewer(s). You should upload this letter as a separate file labeled 'Response to Reviewers'.
- A marked-up copy of your manuscript that highlights changes made to the original version. You should upload this as a separate file labeled 'Revised Manuscript with Track Changes'.
- An unmarked version of your revised paper without tracked changes. You should upload this as a separate file labeled 'Manuscript'.

If you would like to make changes to your financial disclosure, please include your updated statement in your cover letter. Guidelines for resubmitting your figure files are available below the reviewer comments at the end of this letter.

If applicable, we recommend that you deposit your laboratory protocols in protocols.io to enhance the reproducibility of your results. Protocols.io assigns your protocol its own identifier (DOI) so that it can be cited independently in the future. For instructions see: https://journals.plos.org/plosone/s/submission-guidelines#loc-laboratory-protocols. Additionally, PLOS ONE offers an option for publishing peer-reviewed Lab Protocol articles, which describe protocols hosted on protocols.io. Read more information on sharing protocols at https://plos.org/protocols?utm_medium=editorial-email&utm_source=authorletters&utm_campaign=protocols.

We look forward to receiving your revised manuscript.

Kind regards,

Md. Rabiul Awal

Academic Editor

PLOS One

Bangladesh Army University of Science and Technology

Journal Requirements:

When submitting your revision, we need you to address these additional requirements.

1. Please ensure that your manuscript meets PLOS ONE's style requirements, including those for file naming. The PLOS ONE style templates can be found at

https://journals.plos.org/plosone/s/file?id=wjVg/PLOSOne_formatting_sample_main_body.pdf and https://journals.plos.org/plosone/s/file?id=ba62/PLOSOne_formatting_sample_title_authors_affiliations.pdf

2. Thank you for stating in your Funding Statement:

[This research was supported by the Science and Technology Research Project of the Jiangxi Provincial Department of Education (Grant NO. GJJ191219 (Recipient: Xiaoyun Wu)) and the Jiangxi Social Science "14th Five-Year Plan" Fund Project (Grant NO. 24JY37D (Recipient: Xiaoyun Wu)).].

Please provide an amended statement that declares *all* the funding or sources of support (whether external or internal to your organization) received during this study, as detailed online in our guide for authors at http://journals.plos.org/plosone/s/submit-now.  Please also include the statement “There was no additional external funding received for this study.” in your updated Funding Statement.

Please include your amended Funding Statement within your cover letter. We will change the online submission form on your behalf.

3. Thank you for stating the following in your manuscript:

[This research was supported by the Science and Technology Research Project of

the Jiangxi Provincial Department of Education (Grant NO. GJJ191219 (Recipient:

Xiaoyun Wu)) and the Jiangxi Social Science "14th Five-Year Plan" Fund Project

(Grant NO. 24JY37D (Recipient: Xiaoyun Wu)).]

We note that you have provided funding information that is currently declared in your Funding Statement. However, funding information should not appear in the Acknowledgments section or other areas of your manuscript. We will only publish funding information present in the Funding Statement section of the online submission form.

Please remove any funding-related text from the manuscript and let us know how you would like to update your Funding Statement. Currently, your Funding Statement reads as follows:

[This research was supported by the Science and Technology Research Project of the Jiangxi Provincial Department of Education (Grant NO. GJJ191219 (Recipient: Xiaoyun Wu)) and the Jiangxi Social Science "14th Five-Year Plan" Fund Project (Grant NO. 24JY37D (Recipient: Xiaoyun Wu)).]

Please include your amended statements within your cover letter; we will change the online submission form on your behalf.

4. Please amend the manuscript submission data (via Edit Submission) to include author Danting Cao.

5. Please amend your authorship list in your manuscript file to include author Beihe Wu.

6. We note that Figure 2 in your submission contains a map image which may be copyrighted. All PLOS content is published under the Creative Commons Attribution License (CC BY 4.0), which means that the manuscript, images, and Supporting Information files will be freely available online, and any third party is permitted to access, download, copy, distribute, and use these materials in any way, even commercially, with proper attribution. For these reasons, we cannot publish previously copyrighted maps or satellite images created using proprietary data, such as Google software (Google Maps, Street View, and Earth). For more information, see our copyright guidelines: http://journals.plos.org/plosone/s/licenses-and-copyright.

We require you to either (1) present written permission from the copyright holder to publish these figures specifically under the CC BY 4.0 license, or (2) remove the figures from your submission:

1. You may seek permission from the original copyright holder of Figure 2 to publish the content specifically under the CC BY 4.0 license.

We recommend that you contact the original copyright holder with the Content Permission Form (http://journals.plos.org/plosone/s/file?id=7c09/content-permission-form.pdf) and the following text:

“I request permission for the open-access journal PLOS ONE to publish XXX under the Creative Commons Attribution License (CCAL) CC BY 4.0 (http://creativecommons.org/licenses/by/4.0/). Please be aware that this license allows unrestricted use and distribution, even commercially, by third parties. Please reply and provide explicit written permission to publish XXX under a CC BY license and complete the attached form.”

Please upload the completed Content Permission Form or other proof of granted permissions as an "Other" file with your submission.

In the figure caption of the copyrighted figure, please include the following text: “Reprinted from [ref] under a CC BY license, with permission from [name of publisher], original copyright [original copyright year].”

2. If you are unable to obtain permission from the original copyright holder to publish these figures under the CC BY 4.0 license or if the copyright holder’s requirements are incompatible with the CC BY 4.0 license, please either i) remove the figure or ii) supply a replacement figure that complies with the CC BY 4.0 license. Please check copyright information on all replacement figures and update the figure caption with source information. If applicable, please specify in the figure caption text when a figure is similar but not identical to the original image and is therefore for illustrative purposes only.

The following resources for replacing copyrighted map figures may be helpful:

USGS National Map Viewer (public domain): http://viewer.nationalmap.gov/viewer/

The Gateway to Astronaut Photography of Earth (public domain): http://eol.jsc.nasa.gov/sseop/clickmap/

Maps at the CIA (public domain): https://www.cia.gov/library/publications/the-world-factbook/index.html and https://www.cia.gov/library/publications/cia-maps-publications/index.html

NASA Earth Observatory (public domain): http://earthobservatory.nasa.gov/

Landsat: http://landsat.visibleearth.nasa.gov/

USGS EROS (Earth Resources Observatory and Science (EROS) Center) (public domain): http://eros.usgs.gov/#

Natural Earth (public domain): http://www.naturalearthdata.com/

7. Please include captions for your Supporting Information files at the end of your manuscript, and update any in-text citations to match accordingly. Please see our Supporting Information guidelines for more information: http://journals.plos.org/plosone/s/supporting-information.

8. We note that there is identifying data in the Supporting Information file <data.xlsx>. Due to the inclusion of these potentially identifying data, we have removed this file from your file inventory. Prior to sharing human research participant data, authors should consult with an ethics committee to ensure data are shared in accordance with participant consent and all applicable local laws.

Data sharing should never compromise participant privacy. It is therefore not appropriate to publicly share personally identifiable data on human research participants. The following are examples of data that should not be shared:

-Name, initials, physical address

-Ages more specific than whole numbers

-Internet protocol (IP) address

-Specific dates (birth dates, death dates, examination dates, etc.)

-Contact information such as phone number or email address

-Location data

-ID numbers that seem specific (long numbers, include initials, titled “Hospital ID”) rather than random (small numbers in numerical order)

Data that are not directly identifying may also be inappropriate to share, as in combination they can become identifying. For example, data collected from a small group of participants, vulnerable populations, or private groups should not be shared if they involve indirect identifiers (such as sex, ethnicity, location, etc.) that may risk the identification of study participants.

Additional guidance on preparing raw data for publication can be found in our Data Policy (https://journals.plos.org/plosone/s/data-availability#loc-human-research-participant-data-and-other-sensitive-data) and in the following article: http://www.bmj.com/content/340/bmj.c181.long.

Please remove or anonymize all personal information (pid), ensure that the data shared are in accordance with participant consent, and re-upload a fully anonymized data set. Please note that spreadsheet columns with personal information must be removed and not hidden as all hidden columns will appear in the published file.

If the reviewer comments include a recommendation to cite specific previously published works, please review and evaluate these publications to determine whether they are relevant and should be cited. There is no requirement to cite these works unless the editor has indicated otherwise.

[Note: HTML markup is below. Please do not edit.]

Reviewers' comments:

Reviewer's Responses to Questions

**Comments to the Author**

1. Is the manuscript technically sound, and do the data support the conclusions?

Reviewer #1: Yes

Reviewer #2: No

Reviewer #3: Yes

**********

2. Has the statistical analysis been performed appropriately and rigorously?

Reviewer #1: Yes

Reviewer #2: No

Reviewer #3: No

**********

3. Have the authors made all data underlying the findings in their manuscript fully available?

The PLOS Data policy

Reviewer #1: No

Reviewer #2: No

Reviewer #3: Yes

**********

4. Is the manuscript presented in an intelligible fashion and written in standard English?

Reviewer #1: Yes

Reviewer #2: No

Reviewer #3: No

**********

Reviewer #1: This paper examines the relation between digital literacy and quality of employment using data for China. I find the paper interesting and well written. However in my opinion there are several minor issues in the paper which I present below (in order of appearance in the paper) and which, in my view, should be addressed by the author/authors before the paper is considered for publication.

Major Comments

1. I find the title of the paper rather awkward. What does “Microscopic Exploration” mean? I would suggest that the author/authors rewrite the title to make in more appealing to the reader and so that it better reflects the content of the paper.

2. The Introduction lacks one paragraph at the end with a description of the structure of the paper.

3. General comment: employment quality is only defined in line 230-239 whereas it appears for the first time in line 38. I would suggest that the author/authors defines/define employment quality already in the Introduction so much earlier in the paper than in line 230-239.

4. General comment: sustainable employment which appears in the Abstract is nowhere defined in the paper. As it only appears in the Abstract I would suggest to use a different word instead of introducing a definition of it.

5. General comment: the author/authors uses/use OLS as their research method so what they investigate is the relation between independent variables and the dependent variable and not causality. Therefore, in my view, statements like e.g. “Second, it uncovers the dual moderating mechanisms through which digital literacy influences employment quality.” (line 131-133) are way too strong and should be rewritten so as to indicate that the author/authors investigates/investigate a relation between the independent variables and dependent variable.

6. Table 3: I would suggest to at least include the median, minimum and maximum value in case of descriptive statistics for each of the variables, besides the mean and standard deviation.

7. Note for all Tables (line 335): in case of standard errors are those robust standard errors? This is not clear from the description in the Note.

8. Table 5: Model (6) and Model (7)) – I assume that Model (6) is the first stage regression and Model (7) the second stage regression in case of the instrumental variable approach. This should be indicated either in the text or in a note below the Table.

Minor comments:

1. Table 2: in the last row in Column 2 a word seems to be missing: “The importance of using the internet for social”.

2. Figure 2: it’s not exactly clear what is shown on the Legend for that Figure. Is it the number of observations?

3. All Tables and Figures lack an indication of source.

Reviewer #2: The introduction contains definitions and concepts such as digital twin; however, the quality of paragraph writing is not adequate. The purpose and conclusions of the paragraphs are not clear.

The purpose of the study, definitions of concepts, and the necessity and importance of the study are not stated clearly in the introduction.

The theoretical analysis and research hypotheses section, in addition to providing a proper title, contains superficial hypotheses, and the relationship to sustainable labor market development is not clear based on the manuscript's title.

Figure 1 does not provide sufficient explanation and is not bolded in the text.

What does "quality of work" have to do with job opportunities? Can the number of job opportunities necessarily indicate high quality of work?

The analysis framework is unclear, and the results are not presented well. Merely including a discussion and a few results tables does not meet the required quality. The study should provide in-depth discussions and demonstrate a thorough understanding of the issues and draw well-supported conclusions.

Reviewer #3: The title is overly broad and should be revised to include the China-specific context.

The abstract incorrectly uses causal language and should replace it with associative terms.

The abstract contains unnecessary repetition and should be shortened for conciseness.

The literature review lacks foundational theory and should add classical digital literacy frameworks.

The research gap is vague and should be clarified with specific methodological and conceptual gaps.

The digital literacy indicators measure usage rather than skills and should be conceptually justified or refined.

The subjective digital awareness items capture attitudes, not literacy, and should be renamed or clarified.

The entropy method is unjustified and should be supported with reasons for choosing it over PCA or factor analysis.

The employment quality index mixes subjective and objective items and should be validated through factor analysis.

The analysis ignores CFPS sampling weights and should incorporate them for representative results.

The instrumental variable is weakly justified and should be strengthened or replaced.

The PSM approach loses information by dichotomizing employment quality and should use a continuous outcome method.

The models lack multicollinearity testing and should include VIF diagnostics.

Regional fixed effects are missing and should be added to control for regional heterogeneity.

The moderation effect for return expectations is weakly significant and should be interpreted more cautiously or tested further.

The small effect sizes lack context and should include a discussion of practical significance.

The discussion uses causal wording and should adopt non-causal terminology consistent with the research design.

Policy recommendations are too general and should be made more specific to the empirical findings.

The limitations section omits measurement bias issues and should explicitly acknowledge them.

The data availability and ethics statements are incomplete and should provide proper CFPS access details and clarification

**********

what does this mean?). If published, this will include your full peer review and any attached files.

If you choose “no”, your identity will remain anonymous but your review may still be made public.

**Do you want your identity to be public for this peer review?**  For information about this choice, including consent withdrawal, please see our Privacy Policy

Reviewer #1: No

Reviewer #2: No

Reviewer #3: **Yes:** Nor Faiza Abd Rahman

**********

[NOTE: If reviewer comments were submitted as an attachment file, they will be attached to this email and accessible via the submission site. Please log into your account, locate the manuscript record, and check for the action link "View Attachments". If this link does not appear, there are no attachment files.]

To ensure your figures meet our technical requirements, please review our figure guidelines: https://journals.plos.org/plosone/s/figures

You may also use PLOS’s free figure tool, NAAS, to help you prepare publication quality figures: https://journals.plos.org/plosone/s/figures#loc-tools-for-figure-preparation.

NAAS will assess whether your figures meet our technical requirements by comparing each figure against our figure specifications.

# Author comment: Author response to Decision Letter 1

**DOI:** 10.1371/journal.pone.0358194.r002

30 Dec 2025

Response to Reviewers

Manuscript ID: [PONE-D-25-44300]

Title: Research on the Impact and Mechanism of Digital Literacy on Employment Quality: Evidence from the China Family Panel Studies

Dear Editors and Reviewers,

We sincerely thank the editors and reviewers for their valuable time and insightful comments on our manuscript. The constructive suggestions have significantly helped us improve the quality and clarity of our work. We have carefully considered all comments and made corresponding revisions to the manuscript. Point-by-point responses to the comments are listed below. All revisions in the "Revised Manuscript with Track Changes" have been highlighted in red for your review.

Response to Journal Requirements

Requirement 1: Please ensure that your manuscript meets PLOS ONE's style requirements, including those for file naming.

Response: Thank you for your suggestions. We have re-adjusted and revised the entire manuscript in strict accordance with the requirements of the PLOS ONE style template. Kindly review it. If there are any format-related issues, we would greatly appreciate it if you could promptly point them out.

Requirement 2: Please provide an amended statement that declares *all* the funding or sources of support (whether external or internal to your organization) received during this study. Please also include the statement “There was no additional external funding received for this study.” in your updated Funding Statement.

Response: Thank you for your suggestions. We have revised and supplemented the Funding Statement in the most recently submitted cover letter. For your convenience in reviewing, we present below the updated content of the Funding Statement, with the modifications highlighted in red:

Statement

This research was supported by the Science and Technology Research Project of the Jiangxi Provincial Department of Education (Grant NO. GJJ191219 (Recipient: Xiaoyun Wu)) and the Jiangxi Social Science "14th Five-Year Plan" Fund Project (Grant NO. 24JY37D (Recipient: Xiaoyun Wu)). There was no additional external funding received for this study.

Requirement 3: Please remove any funding-related text from the manuscript and let us know how you would like to update your Funding Statement.

Response: Thank you for your suggestions. All text related to funding has been removed from the manuscript, and relevant supplements and explanations have been provided in the Funding Statement in the cover letter. For specific details, please refer to the content marked in red in the response to Requirement 2 above.

Requirement 4: Please amend the manuscript submission data (via Edit Submission) to include author Danting Cao.

Response: Thank you for your suggestions. We have appended the relevant information of author Danting Cao in the Revised Manuscript with Track Changes and highlighted it in red font. In addition, the "Authorship Change Request form" has been uploaded as a submission file with the file type label "Other". Kindly review it once more.

Requirement 5: Please amend your authorship list in your manuscript file to include author Beihe Wu.

Response: Thank you for your suggestions. We have appended the relevant information about author Beihe Wu in the revised version, utilized the revision function, and highlighted it in red font. Kindly review it once more. It should be noted that, as stated in the Acknowledgments section of the manuscript, "Since our institution has not implemented institutional email authentication, the email address used for communication during the submission process was provided by Beihe Wu. To clarify, Beihe Wu was not involved in the study design, data collection and analysis, decision to publish, or manuscript preparation." Therefore, despite the email address being wubeihe@stu.jxau.edu.cn, the corresponding author of the manuscript remains Danting Cao.

Requirement 6: We note that Figure 2 in your submission contains a map image which may be copyrighted. We require you to either (1) present written permission from the copyright holder to publish these figures specifically under the CC BY 4.0 license, or (2) remove the figures from your submission.

Response: Thank you for your suggestions. Following a consultation within our team and taking into account that Figure 2 in the manuscript may be protected by copyright, we have decided to remove Figure 2 from this submission. Please review the revised version.

Requirement 7: Please include captions for your Supporting Information files at the end of your manuscript, and update any in-text citations to match accordingly.

Response: Thank you for your suggestion. During this submission process, apart from adding explanatory text for the supporting information file at the end of the manuscript (lines 920–921), we also considered that the data in the supporting information file might involve the privacy of respondents. Consequently, we have provided the ethical review certificate requested by the reviewers and uploaded it along with the research data to the "Supporting Information File".

Requirement 8: We note that there is identifying data in the Supporting Information file <data.xlsx>. Due to the inclusion of these potentially identifying data, we have removed this file from your file inventory. Please remove or anonymize all personal information (pid), ensure that the data shared are in accordance with participant consent, and re-upload a fully anonymized data set. Please note that spreadsheet columns with personal information must be removed and not hidden as all hidden columns will appear in the published file.

Response: Thank you for your valuable suggestions. We have anonymized all personal information (PID) within the support information file <data.xlsx> and re-uploaded a fully anonymous dataset. We earnestly request that you conduct a review once more.

Response to Reviewer 1

Major Comment 1: What does “Microscopic Exploration” mean? I would suggest that the author/authors rewrite the title to make in more appealing to the reader and so that it better reflects the content of the paper.

Response: We express our sincere gratitude for your valuable suggestions. We wholeheartedly concur with your viewpoints. After thorough deliberation by our team and taking into account the content of the article, we have decided to change the original title of the paper to "Research on the Impact and Mechanism of Digital Literacy on Employment Quality: Evidence from the China Family Panel Studies". This revision narrows the title's scope and emphasizes the micro-data source (CFPS) employed, rendering the title more consistent with the empirical background and contributions of the research. Once again, we appreciate your meticulous review and constructive suggestions.

Major Comment 2: The Introduction lacks one paragraph at the end with a description of the structure of the paper.

Response: We wholeheartedly concur with your viewpoints. In the revised manuscript, specifically in the last paragraph of the introduction (lines 180-185), we have incorporated a description of the paper's structure and highlighted it in red. For your review, the newly added content is presented as follows:

The remainder of this paper is organized as follows: Section 2 consists of the theoretical analysis and research hypotheses. Section 3 describes the methodology and data, including the research methodology, variable selection, and data sources. Section 4 presents and analyzes the main findings. Further discussion is provided in Section 5. Finally, key conclusions and policy implications are summarized in Section 6.

Major Comment 3: Employment quality is only defined in line 230-239 whereas it appears for the first time in line 38. I would suggest that the author/authors defines/define employment quality already in the Introduction so much earlier in the paper than in line 230-239.

Response: We express our sincere gratitude for your valuable suggestions. In accordance with your suggestions, we have incorporated a definition of the concept of employment quality in lines 66-73 of the "Revised Manuscript with Track Changes" and revised the definition in lines 230-239 of the initial draft. Kindly review it once more.

Major Comment 4: Sustainable employment which appears in the Abstract is nowhere defined in the paper. As it only appears in the Abstract I would suggest to use a different word instead of introducing a definition of it.

Response: We fully concur with your opinion. Consequently, in the "revised annotated manuscript," we have substituted the term "sustainable employment" with "employment quality" and revised the abstract (lines 14-36 in the revised manuscript). For your ease of review, we present the revised abstract as follows and earnestly request that you conduct another review:

Abstract

In the digital era, digital literacy has emerged as a critical determinant of employment quality within the labor market. Using nationally representative data from the 2022 China Family Panel Studies (CFPS), this study constructs multidimensional indices for digital literacy and employment quality. Ordinary least squares (OLS) regressions, robustness checks, and moderating effect models were employed to examine the relationships and underlying mechanisms between these variables. The findings suggest that digital literacy is significantly associated with higher employment quality. This relationship is positively moderated by return expectations and negatively moderated by human capital, indicating a more pronounced association among low-skilled workers with high return expectations. Regional heterogeneity reveals the most significant associations in Western China and urban agglomerations, attributable to higher marginal returns on digital skills in underdeveloped regions and denser digital economies in clustered zones. Digital literacy appears as a pivotal correlate of employment quality, particularly for vulnerable groups and lagging regions. Policy interventions should prioritize (1) inclusive, tiered digital training programs targeting low-skilled workers; (2) regionally differentiated strategies; and (3) leveraging urban clusters as hubs for cross-regional resource sharing. These approaches can narrow digital divides and advance sustainable, equitable, and high-quality employment in the digital era.

Major Comment 5: The author/authors uses/use OLS as their research method so what they investigate is the relation between independent variables and the dependent variable and not causality. Therefore, in my view, statements like e.g. “Second, it uncovers the dual moderating mechanisms through which digital literacy influences employment quality.” (line 131-133) are way too strong and should be rewritten so as to indicate that the author/authors investigates/investigate a relation between the independent variables and dependent variable.

Response: We sincerely appreciate your valuable comments on this article. We wholeheartedly accept and firmly concur with your perspectives regarding the limitations of ordinary least squares (OLS) in causal inference and the concern that certain sections of the text excessively emphasize "influence mechanisms" instead of "correlation." As you astutely pointed out, the OLS method primarily reveals the statistical association between variables, whereas strict causal inference necessitates more rigorous research designs (such as randomized experiments, instrumental variable methods, and natural experiments). Consequently, we will follow your advice and revise the relevant expressions in the text. The initial draft's original text (lines 131–133), "Second, it uncovers the dual moderating mechanisms through which digital literacy influences employment quality," will be revised to "(3) to uncover the moderating mechanisms of return expectations and human capital in their relationship (lines 171-173 in the revised manuscript)." Moreover, based on your suggestions, we have conducted systematic revisions of the entire text, with a specific emphasis on the "Introduction (lines 103-179 in the revised manuscript)," "Discussion (lines 729-833 in the revised manuscript)," "Conclusions and Implications (lines 835-919 in the revised manuscript)," and "Abstract (lines 15-36 in the revised manuscript)" sections. We have modified all wording that could potentially imply causal relationships (e.g., "influence," "promote," "lead to," etc.) to more precise non-causal expressions (e.g., "correlated with," "positively associated with," "association," etc.). These revisions ensure consistency between the terminology used and the observational design of the study, while preserving the clarity and academic rigor of the research conclusions. Once again, we are grateful for your incisive comments, which are highly beneficial for enhancing the academic rigor of this article. We eagerly anticipate your further guidance.

Major Comment 6: Table 3: I would suggest to at least include the median, minimum and maximum value in case of descriptive statistics for each of the variables, besides the mean and standard deviation.

Response: We have incorporated the median, minimum, and maximum values of each variable into the descriptive statistics in Table 3. For your review, the revised Table 3 is presented below:

Table 3. Variable definition and descriptive statistics.

Variable Variable Description Mean Std. Dev. Min. Max. Med.

Explained variable Digital literacy Comprehensive index calculated using the entropy method 0.584 0.256 0.028 0.999 0.580

Core explanatory variable Employment quality Comprehensive index calculated using the entropy method 0.973 0.224 0.015 1.551 0.978

Moderator variables Expected returns Mean score of measurement items 3.261 0.561 1 5 3

Human capital Educational attainment: 1=Primary school or below; 2=Junior high school; 3=Senior high school; 4=College or above 2.650 1.098 1 4 3

Control variables Gender 1=Male; 0=Female 0.557 0.497 0 1 1

Age Actual age 37.989 10.491 18 60 36

Marital status 1=Married; 0=Unmarried 0.753 0.431 0 1 1

Political status 1=Communist Party member; 0=Non-Communist Party member 0.120 0.325 0 1 0

Household registration type 1=Non-agricultural; 0=Agricultural 0.371 0.483 0 1 0

Major Comment 7: Note for all Tables (line 335): in case of standard errors are those robust standard errors? This is not clear from the description in the Note.

Response: Thank you for your comprehensive review and corrections of the paper. Your inquiry regarding whether the standard errors presented in the tables were robust standard errors is of great significance. In the original description, we failed to clearly state this fact, and we offer our sincere apologies for this oversight. We have conducted a thorough examination of the original text. To provide clarity, in the benchmark regression (Table 4) and other primary regression tables, robust standard errors were utilized for the regressions. This method was adopted to address potential heteroskedasticity in the cross-sectional data and to improve the reliability of the coefficient significance tests. Therefore, to ensure the rigor and transparency of the paper, we will implement the following revisions based on your suggestions:

In the note of Table 5 (lines 524-525 in the revised manuscript), we will explicitly change the current statement "Standard errors are presented in parentheses" to "Robust standard errors are presented in parentheses." Moreover, we have included an explanation regarding the standard error setting in the "Model Specification" section (lines 470-472 in the revised manuscript). For instance, after describing the benchmark regression model, we will add: "In all OLS regressions, robust standard errors are employed to ensure the reliability of statistical inference."

Thank you once again for your meticulous and professional review comments, which are essential for enhancing the methodological rigor of our paper.

Major Comment 8: Table 5: Model (6) and Model (7) – I assume that Model (6) is the first stage regression and Model (7) the second stage regression in case of the instrumental variable approach. This should be indicated either in the text or in a note below the Table.

Response: I sincerely appreciate your thorough review of the instrumental variable (IV) analysis section in the text. Your observation concerning the labeling of Model (6) and Model (7) in Table 5 is completely accurate. This was, in fact, an oversight on our end and might have led to confusion among readers. To address this issue, we will implement the following revision as per your suggestion:

Insert a note beneath Table 6 (lines 528-529 in the revised manuscript) indicating: "Note: Model (6) showcases the first-stage regression results of the instrumental variable approach, and Model (7) showcases the corresponding second-stage results."

Once again, I am grateful for your invaluable professional feedback, which is of great significance for improving the precision and readability of our paper.

Minor comment 1: Table 2: in the last row in Column 2 a word seems to be missing: “The importance of using the internet for social”.

Response: We sincerely appreciate your thorough review of the paper and for identifying this oversight. The issue you raised is completely valid; there is, in fact, a missing word in the variable description of the last row of Table 2. The original text "The importance of using the internet for social" should be revised to "The importance of using the internet for social activities". This omission resulted from an editing oversight. The complete expression is intended to measure an individual's perception of the significance of "using the internet for social activities". We will promptly rectify this error in the revised version and will also conduct a systematic check of all tables in the full-text to ensure the comprehensiveness and accuracy of the variable descriptions. Once again, we express our sincere gratitude for your rigorous review.

Minor comment 2: Figure 2: it’s not exactly clear what is shown on the Legend for that Figure. Is it the number of observations?

Response: Thank you for your thorough review of the figures in the text. We fully comprehend and acknowledge your concern regarding the ambiguity of the legend in Figure 2. Moreover, in accordance with the revision suggestions provided by the journal for Figure 2, we have decided to remove it to meet the journal's requirements for the final acceptance of the article.

Minor comment 3: All Tables and Figures lack an indication of source.

Response: Thank you once again for raising a crucial normative issue. You have correctly pointed out that the data sources for all the tables and figures in the text were not clearly specified, which was indeed an oversight on our part during the manuscript-writing process. In response to your comment, the following revisions will be made: A uniform data source will be added for all tables and charts based on the China Family Panel Studies (CFPS) data. As indicated in the "Data Sources" section of this article, the primary data for this study are derived from the 2022 China Family Panel Studies (CFPS). In this revision, we will uniformly append the note "Note: Data source: China Family Panel Studies (CFPS), 2022 (the same applies to the following tables)" at the end of the annotation for Table 1 (line 342 in the revised manuscript). Moreover, for the theoretical framework diagram (Figure 1), we will indicate in its caption that it was drawn by the authors based on the research analysis framework and add "Note: Source: Authors’ construction" (line 310 in the revised manuscript). Once again, we appreciate your rigorous and meticulous review.

Response to Reviewer 2

Comment 1: The introduction contains definitions and concepts such as digital twin; however, the quality of paragraph writing is not adequate. The purpose and conclusions of the paragraphs are not clear.

Response: We extend our sincere gratitude for your highly valuable comments on this article. We have meticulously reviewed your suggestions and thoroughly reflected on the introduction section. In response to your two points, the following is provided:

1. Concerning the "definition of concepts such as digital twin": Upon verification, the concept of "digital twin" is not covered in the introduction part of this article. Our main focus is on digital literacy and employment quality, and we elaborate on their definitions, the current research status, and the research gap addressed in this article. It is possible that the term usage or paragraph structure has led to misunderstandings. In the revision, we will further clarify the core concepts to prevent the emergence of irrelevant or ambiguous terms.

2. Regarding "insufficient writing quality of paragraphs, unclear purpose and conclusion": We acknowledge your criticism regarding the logic and clarity of expression in the introduction paragraphs. During the revision, we will conduct structural optimization of the introduction section. The specific measures are as follows: (1) Clearly articulate the purpose of each paragraph at the beginning; (2) Reinforce the summary at the end of each paragraph to emphasize its contribution to the overall discussion; (3) Simplify certain long sentences to enhance coherence and hierarchy of expression; (4) Strengthen the logical flow from "research background → literature review → research gap → contribution of this article" to make the introduction section more instructive and persuasive.

We sincerely appreciate your comprehensive review and constructive suggestions, which are extremely beneficial for enhancing the academic expression and logical clarity of this article. We will carefully revise the entire article in accordance with your suggestions and mark all changes in the revised version, with the hope of better meeting the requirements of the journal.

Comment 2: The purpose of the study, definitions of concepts, and the necessity and importance of the study are not stated clearly in the introduction.

Response: Thank you for your valuable comments on the introduction of this article. We fully acknowledge your critique that the original text was insufficiently prominent and clear regarding the research purpose, the definition of core concepts, and the necessity and significance of the research. In response to your suggestions, we have systematically revised the introduction section.

The main improvements are as follows:

Clarify the research purpose: A dedicated paragraph has been added at the end of the introduction to explicitly enumerate the four specific objectives of this study (lines 166-179 in the revised manuscript).

Focus on defining core concepts: The originally scattered definitions of "digital literacy" and "employment quality" have been integrated and strengthened, and a clear and multi-dimensional operational definition is provided within a single paragraph (lines 61-73 in the revised manuscript).

Enhance the necessity and significance of the research: After the literature review, a paragraph has been added to directly address the value and urgency of this research from the perspectives of "Methodologically" and "Conceptually" (lines 121-153 in the revised manuscript).

The following is the fully revised introduction, with the main modifications and additions presented in bold and red font:

Against this backdrop, the relationship between digital literacy and employment quality has emerged as a core issue. This study conceptualizes "digital literacy" as the comprehensive capacity of workers to acquire, process, and apply digital information and technology within a digital environment, encompassing both objective operational skills and subjective cognitive inclinations [4]. Meanwhile, drawing on the "Decent Work" framework of the International Labour Organization (ILO), this study defines "employment quality" as the comprehensive manifestation of the objective economic benefits and subjective value realization that workers attain during employment, and assesses it from multiple dimensions, including the objective dimension (wages, employment security, career development opportunities) and the subjective dimension (job satisfaction) [5] (lines 61-73 in the revised manuscript).

A substantial body of research has demonstrated that digital literacy significantly contributes to facilitating access to employment opportunities [12], enhancing employability [13], fostering entrepreneurship [14], and promoting innovative work behaviors [15]. However, when it comes to the more holistic and policy-critical outcome of “high-quality employment,” the extant literature exhibits discernible gaps at both methodological and conceptual levels, which this study aims to address (lines 121-128 in the revised manuscript).

Methodologically, three main shortcomings are evident. First, there is a prevalent reliance on oversimplified measurement. Employment outcomes are often reduced to binary employment status or singular metrics like income, failing to capture the multidimensional nature of employment quality that encompasses both objective conditions (e.g., security, prospects) and subjective perceptions (e.g., satisfaction) [16]. Similarly, digital literacy is frequently proxied by basic indicators such as internet access or usage frequency [17], neglecting its composite character involving both operational skills and cognitive dispositions. Second, there is a lack of systematic investigation into boundary conditions. While a direct positive link is often assumed, the moderating mechanisms (e.g., how individual expectations or existing human capital alter the strength of this link) remain underexplored and poorly tested. Third, studies often overlook substantial heterogeneity across regions and developmental contexts, treating the digital literacy-employment quality relationship as uniform despite plausible variations due to differing economic structures and digital infrastructure (lines 129-145 in the revised manuscript).

Conceptually, the literature is limited by narrow framing. On one hand, the conceptualization of digital literacy has not kept pace with its evolving complexity, often remaining confined to technical proficiency without integrating the awareness and evaluative capacities crucial for effective application in the workplace [9]. On the other hand, the concept of employment quality is seldom grounded in integrated frameworks like the ILO's Decent Work agenda [5], which would allow for a balanced assessment of both economic and psychosocial dimensions (lines 146-153 in the revised manuscript).

Based on this, this study employs the 2022 China Family Panel Studies (CFPS) data. Through empirical analysis, it aims to achieve the following specific research objectives: (1) to establish a multi-dimensional comprehensive evaluation index system for digital literacy and employment quality; (2) to empirically verify the overall influence of digital literacy on workers’ employment quality; (3) to uncover the moderating mechanisms of return expectations and human capital in their relationship; (4) to investigate the heterogeneity of the aforementioned impacts across different regions (eastern, central, and western regions) and areas with varying levels of resource agglomeration (urban agglomerations and non-urban agglomerations). This study anticipates providing micro-level evidence and policy implications for promoting higher-quality, fairer, and more sustainable labor market development during the digitalization process through the above analyses (lines 166-179 in the revised manuscript).

We firmly believe that subsequent to the aforementioned revisions, the logic within the introduction has become more lucid, the core elements are more pronounced, and it can more efficaciously guide readers to comprehend the value and design of this research. Once again, we sincerely express our gratitude for your invaluable time and professional insights, which have significantly elevated the quality of this article.

Comment 3: The theoretical analysis and research hypotheses section, in addition to providing a proper title, contains superficial hypotheses, and the relationship to sustainable labor market development is not clear based on the manuscript's title.

Response: We sincerely appreciate your in-depth and constructive comments on the "Theoretical Analysis and Research Hypotheses" section. We fully comprehend and concur with your critique that the original section had an improper title, the hypothesis argumentation was rather superficial, and it was not closely associated with the theme of "sustainable development of the labor market" in the article title. These comments accurately identified the deficiencies in the theoretical depth and overall logical consistency of the original manuscript. Moreover, given that your perspective aligns with the suggestion of another external reviewer regarding the modification of our article title, we have not only revised the original article title but also added chapter titles to the "2. Theoretical Mechanism Analysis and Research Hypotheses" section to more effectively reflect the core content and objectives of this section. For your review, we present the revised content as follows (the bolded and red-colored parts are the key revisions and additions):

From the perspective of sustainable labor market development, the "incentive effect" triggered by high return expectations can motivate workers to continuously invest in digital human capital, thereby establishing a virtuous cycle of "learning → application → improvement → re-learning". This contributes to maintaining the dynamic update of labor skills, addressing the challenges posed by digital transformation, and represents a crucial psychological mechanism for achieving long-term human capital accumulation and market vitality (lines230-238 in the revised manuscript).

In conclusion, individuals possessing high-level human capital generally exhibit strong employability as a result of their educational attainments. This situation may give rise to a diminishing marginal benefit of digital literacy. Conversely, for individuals with low human capital, digital literacy serves as a vital instrument to compensate for their skill deficits and enhance their employment prospects. The identification of this "negative moderation" phenomenon holds substantial "inclusive" significance for the sustainable development of the labor market. It suggests that well-targeted policies aimed at enhancing digital literacy can effectively empower disadvantaged workers, mitigate employment disparities stemming from differences in basic human capital, and foster more inclusive and equitable growth—this stands as one of the core objectives of sustainable development (lines 262-275 in the revised manuscript).

Based on the aforementioned analysis, this study presents the empirical analysis framework illustrated in Fig 1. This framework not only delineates the direct path through which digital literacy influences employment quality (H1) and two crucial boundary conditions (H2 and H3), but also links it with the multi-dimensional objectives of sustainable employment development at the underlying logic level. Digital literacy directly contributes to the efficiency and stability of the labor market by improving individual employment quality (H1); the moderating effect of return expectations (H2) reveals the psychological motivation for sustaining investment in human capital; whereas the moderating effect of human capital (H3) emphasizes the potential of digital literacy to facilitate inclusive growth and reduce group disparities. The verification of these three hypotheses constitutes a systematic exploration, from the micro-individual level, of the impact of individual digital literacy on employment quality in the digital age. It visually presents a summary of the relationships among the core variables (digital literacy, employment quality, return expectations, human capital), along with the three major hypotheses (H1, H2, H3) that this study intends to test. Among these, the core explanatory variable, "digital literacy," directly influences the explained variable, "employment quality," via the main path (corresponding to Hypothesis H1). The two moderating paths respectively demonstrate the moderating effects of "return expectations" and "human capital" on the aforementioned main effect (corresponding to Hypotheses H2 and H3). It is hypothesized that "return expectations" positively strengthen the impact of digital literacy (denoted by "+"), whereas "human capital" is hypothesized to negatively attenuate this impact (denoted by "−"). All analyses are carried out while controlling for a series of individual characteristic variables. This framework is designed to visualize the causal relationship network derived from the theoretical analysis section of this paper (lines 279-308 in the revised manuscript).

We firmly believe that through the aforementioned revisions, the theoretical depth of this section has been strengthened, and the logical coherence with the title and core theme of the paper has been significantly improved. Once again, we sincerely express our gratitude for your incisive review, which has greatly assisted us in refining the theoretical framework and presentation of our research.

Comment 4: Figure 1 does not provide sufficient explanation and is not bolded in the text.

Response: We sincerely appreciate your valuable comments regarding Figure 1 (the empirical analysis framework diagram). We wholeheartedly acknowledge your critique that the initial manuscript failed to offer adequate explanations for Figure 1 and did not highlight it conspicuously when referencing it in the main text. This oversight had an impact on readers' comprehension of and attention to this framework diagram. In light of your comments, we have implemented the following revisions to the relevant sections of the main text and the caption of Figure 1 (lines 279-308 in the revised manuscript):

Based on the aforementioned analysis, this study presents the empirical analysis framework illustrated in Fig 1. This framework not only delineates the direct path through which digital literacy influences employment quality (H1) and two crucial boundary conditions (H2 and H3), but also links it with the multi-dimensional objectives of sustainable employment development at the underlying logic level. Digital literacy directly contributes to the efficiency and stability of the labor market by improving individual employment quality (H1); the moderating effect of return expectations (H2) reveals the psychological motivation for sustaining investment in human capital; whereas the moderating effect of human capital (H3) emphasizes the potential of digital literacy to facilitate inclusive growth and reduce group disparities. The verification of these three hypotheses constitutes a systematic exploration, from the micro-individual level, of the impact of individual digital literacy on employment quality in the digital age. It visually presents a summary of the relationships among the core variables (digital literacy, employment quality, return expectations, human capital), along with the three major hypotheses (H1, H2, H3) that this study intends to test. Among these, the core explanatory variable, "digital literacy," directly influences the explained variable, "employment quality," via the main path (corresponding to Hypothesis H1). The two moderating paths respectively demonstrate the moderating effects of "return expectations" and "human capital" on the aforementioned main effect (corresponding to Hypotheses H2 and H3). It is hypothesized that "return expectations" positively strengthen the impact of digital literacy (denoted by "+"), whereas "human capital" is hypothesized to negatively attenuate this impact (denoted by "−"). All analyses are carried out while controlling for a series of individual characteristic variables. This framework is designed to visualize the causal relationship network derived from the theoretical analysis section of this paper (lines 279-308 in the revised manuscript).

Comment 5: What does "quality of work" have to do with job opportunities? Can the number of job opportunities necessarily indicate high quality of work?

Response: We express our sincere gratitude for bringing up this significant question, which has enabled us to clarify a crucial concept in our research that could have been ambiguous. Your perspective is entirely accurate: the quantity or accessibility of job opportunities does not necessarily signify high - quality jobs. Our research is in full agreement with this stance and does not merely equate the two. Certain expressions in the original manuscript may not have been sufficiently precise, resulting in misunderstandings. The following is a detailed response to your question and an explanation of the revisions we will implement in the relevant sections of the text.

In this study, "employment quality" is defined as a multi-dimensional and comprehensive construct. Its measurement system explicitly encompasses both objective and subjective components, with specific indicators such as labor remuneration, employment security (including contracts and social security), career development opportunities (such as promotion), and job satisfaction (related to income, safety, environment, working time, and career prospects) (see Table 1). "Job opportunities," in contrast, primarily denote the accessibility or probability of obtaining employment. They are merely a potential precondition or avenue that influences employment quality, rather than being equivalent to the quality itself. A large number of job opportunities may provide more options, yet they do not ensure that the jobs are of high quality (for example, they could be low-paying, insecure, or unstable positions).

We recognize that in the theoretical analysis section (Section 2) and the literature review when discussing the impact of digital literacy, the original article cited "obtaining better job opportunities" as one of its positive consequences. This phrasing might inadvertently obscure the distinction between "opportunity" and "quality." To articulate our theoretical logic more accurately, we will modify the theoretical analysis part of the original article from "... thereby improving work productivity and increasing their chances of securing better job opportunities and higher wages in the labor market [11, 16]" to "... thereby improving work productivity. This enhanced capacity enables them not only to access a wider array of job opportunities but, more crucially, to obtain positions featuring higher wages, better employment security, and greater prospects for career development—core aspects of employment quality [14, 19] (lines 194-199 in the revised manuscript)."

We hereby reiterate our sincere gratitude for your incisive review. Your comments have impelled us to make essential refinements to the articulation of our core concepts. During the comprehensive revision of the manuscript, we pledge to strictly distinguish between "job opportunities" as a channel or antecedent factor and "job quality" as a multi-dimensional outcome, thereby guaranteeing the rigor of both theoretical logic and textual expression. The aforementioned modifications will be directly integrated into the revised version.

Comment 6: The analysis framework is unclear, and the results are not presented well. Merely including a discussion and a few results tables does not meet the required quality. The study should provide in-depth discussions and demonstrate a thorough understanding of the issues and draw well-supported conclusions.

Response: We sincerely express our gratitude for your valuable comments regarding the analysis framework and the discussion of the results in this article. We thoroughly comprehend and concur with your critique that the initial analysis framework was inadequately explained, the presentation of the results was relatively superficial, and the depth of the discussion and the overall understanding of the research questions required enhancement. We have systematically rewritten and deepened the "Discussion" section to substantially improve the logic, analytical depth, and quality of the argumentation in the paper. For your review, we present the revised content as follows:

Next, this study employed moderating effect analysis to deconstruct the impact mechanisms of digital literacy. Firstly, the positive moderating effect of return expectation (H2 is supported) highlights the "catalyst" role of psychological motivation. The significant positive interaction term is consistent with the view that for workers who firmly believe that "efforts will be rewarded," the association between digital skills and employment benefits may be stronger. This finding is highly consistent with expectancy theory [45], which connects micro-level individual psychological expectations with macro-level skill investment behavior. In practice, this implies that if digital training programs can be integrated with clear career prospects and successful case demonstrations to stimulate learners' positive expectations, they will be more effective in converting skills into actual improvements in employment quality [46, 47]. This offers important implications for the design of "incentive-compatible" skill intervention policies. Secondly, the negative moderating effect of human capital (H3 holds) reveals a phenomenon with significant policy implications. The significant negative interaction term indicates that the positive association between digital literacy and employment quality is more pronounced among workers with lower education levels and weaker traditional human capital. This challenges the simplistic assumption that the returns to digital skills increase linearly with educational attainment [22]. The underlying logic is that for high-human-capital groups, their employment competitiveness is largely established by educational qualifications and professional experience, and the addition of digital literacy may lead to diminishing marginal benefits. In contrast, for low-human-capital groups, digital literacy plays a crucial role as a "skill compensator" or "threshold crosser," effectively compensating for their disadvantages in traditional human capital and opening up pathways to higher-quality employment [48]. This finding is of core value for considering how to utilize digitalization to promote social inclusion and alleviate structural employment inequality (lines 729-758 in the revised manuscript).

In terms of regional gradients, the positive association of digital literacy with employment quality is most pronounced in the western region, followed by the eastern region, and relatively least significant in the central region. This contradicts the intuitive assumption that "the more developed the economy, the higher the return," yet it aligns with the theory of marginal returns of digital infrastructure [51]. In the western region, where digital infrastructure is in the catch-up phase, workers can acquire a substantial competitive edge by mastering basic digital skills, yielding high marginal returns. In the eastern region, where the digital economy is well-established, the penetration rate of digital skills is high and competition is intense, leading to a relatively smaller excess return from further improvement. In the central region, which is in the "difficult period" of industrial transformation and upgrading, the demand for high-level digital skills in traditional industries is inadequate, and emerging industries have not fully matured, resulting in relatively limited application opportunities for digital skills and impeding their value realization [38]. This pattern implies that digital literacy policies must be precisely tailored to the regional development stage and industrial structure (lines 763-781 in the revised manuscript).

In the context of agglomeration patterns, the intensity of the effect in urban agglomeration regions is notably higher than that in non-urban agglomeration regions. This emphasizes the amplification effect of resource agglomeration [52, 53]. Urban agglomerations are not merely the "high ground" of digital infrastructure but also the "incubators" and "clusters" of new business models and new occupations in the digital economy. The dense industrial network, frequent knowledge dissemination, and abundant application scenarios offer a favorable environment for the realization of the value of digital skills. Conversely, the digital transformation of industries in non-urban agglomeration regions progresses relatively slowly, and the application scenarios of digital skills are restricted, thus diminishing their role in enhancing employment quality. This highlights the significance of leveraging urban agglomerations as hubs to promote the digital transformation and coordinated development of digital talents in the surrounding areas (lines 782-796 in the revised manuscript).

We firmly believe that through the aforementioned systematic revisions, the paper's analytical framework has been clarified, and the presentation and discussion of the results have achieved the necessary depth and academic rigor. Once again, we sincerely thank you for your incisive review comments, which are highly significant in improving the overall quality of this research.

Response to Reviewer 3

Comment 1: The title is overly broad and should be revised to include the China-specific context.

Response: We express our sincere gratitude for your valuable comments on this article. We wholeheartedly concur with your suggestion that the original title had an overly broad scope and failed to comprehensively reflect the specific national context and data foundation of this research. Furthermore, your perspective aligns with that of another external reviewer. Consequently, we have revised the title to "Research on the Impact and Mechanism of Digital Literacy on Employment Quality: Evidence from the China Family Panel Studies." This revision narrows the title's scope and emphasizes the micro-data source (CFPS) employed, rendering the title more consistent with the empirical background and contributions of the research. Once again, we appreciate your meticulous review and constructive suggestions.

Comment 2: The abstract incorrectly uses causal language and should replace it with associative terms.

Response: Thank you very much for your invaluable suggestions regarding the abstract of this article. We wholeheartedly concur with your viewpoints and have systematically substituted the expressions in the original abstract that could potentially imply causal relationships with more rigorous expressions denoting correlation and association in the revised abstract. The specific modifications are as follows:

Abstract (lines 14–36 in the revised manuscript)

In the digital era, digital literacy has emerged as a critical determinant of employment quality within the labor market. Using nationally representative data from the 2022 China Family Panel Studies (CFPS), this study constructs multidimensional indices for digital literacy and employment quality. Ordinary least squares (OLS) regressions, robustness checks, and moderating effect models were employed to examine the relationships and underlying mechanisms between these variables. The findings suggest that digital literacy is significantly associated with higher employment quality. This relationship is positively moderated by return expectations and negatively moderated by human capital, indicating a more pronounced association among low-skilled workers with high return expectations. Regional heterogeneity reveals the most significant associations in Western China and urban agglomerations, attributable to higher marginal returns on digital skills in underdeveloped regions and denser digital economies in clustered zones. Digital literacy appears as a pivotal correlate of employment quality, particularly for vulnerable groups and lagging regions. Policy interventions should prioritize (1) inclusive, tiered digital training programs targeting low-skilled workers; (2) regionally differentiated strategies; and (3) leveraging urban clusters as hubs for cross-regional resource sharing. These approaches can narrow digital divides and advance sustainable, equitable, and high-quality employment in the digital era.

The aforementioned revisions have modified the causal expressions, such as "improves employment quality" and "effect is moderated," to phrasing more suitable for observational studies, like "is significantly associated with higher employment quality" and "relationship is moderated," in order to more precisely reflect that the findings of this study are based on cross-sectional data correlation analysis. Once again, I express my gratitude for your detailed review and valuable suggestions.

Comment 3: The abstract contains unnecessary repetition and should be shortened for conciseness.

Response: We wholeheartedly concur with your viewpoint and have methodically rewritten the original abstract in the revised version to render the content of the abstract more succinct. You may refer to lines 14 to 36 of the revised draft or directly consult my response to your second comment. Thank you again for your detailed review and valuable suggestions.

Comment 4: The literature review lacks foundational theory and should add classical digital literacy frameworks.

Response: Thank you for your valuable suggestions regarding the literature review section. We wholeheartedly concur with your view that the original text was deficient in its review of theoretical foundations and classic frameworks. Consequently, we have incorporated a paragraph in the literature review part of the "Introduction" (lines 103-120 in the revised manuscript) to systematically examine the classic theoretical foundations and representative frameworks of digital literacy, aiming to enhance the theoretical basis of the research. The specific modifications are as follows:

To ground this study in established theoretical discourse, it is essential to acknowledge foundational digital literacy frameworks. Digital divide theory conceptualizes digital inequality across multiple levels—from access to skills, usage, and tangible outcomes—providing a structural lens to analyze how literacy disparities translate into labor market inequities [10]. Complementing this, UNESCO's Global Digital Literacy Framework defines it as a life skill encompassing seven core competency areas (including device and software operation, information and data literacy, communication and collaboration, and safety), emphasizing its role in personal development and social participation [11]. At the policy level, the European Union's DigComp framework has been instrumental in operationalizing digital competence for citizens, outlining five key areas (information and data literacy, communication and collaboration, digital content creation, safety, and problem-solving) that align closely with the demands of evolving labor markets . These theoretical and policy-oriented frameworks collectively affirm that digital literacy is not merely a technical skill but a multidimensional capability critical for socio-economic integration and opportunity (lines 103-120 in the revised manuscript).

This supplementation incorporates the classic theories and frameworks of digital literacy (UNESCO, EU DigComp) into the discussion, thereby clarifying the theoretical foundation of this research. Additionally, it establishes a dialogue with and connects to the two-dimensional index system of "objective digital ability–subjective digital awareness" developed in the subsequent text, thus enhancing the theoretical depth and comprehensiveness of the literature review. Once again, I express my sincere gratitude for your professional review and constructive suggestions.

Comment 5: The research gap is vague and should be clarified with specific methodological and conceptual gaps.

Response: Thank you very much for your valuable comments on this article. Your suggestion, stating that "the research gap is expressed ambiguously and should be specifically clarified from the methodological and conceptual levels," is highly pertinent and will significantly contribute to our efforts in further enhancing the logical clarity and academic rigor of our paper. In line with your advice, we have made crucial revisions and improvements to the description of the "research gap" in the introduction section. The essence of the revision is to clearly delineate the previously rather general limitations of existing research into specific gaps at the methodological and conceptual levels and directly associate them with the contributions this study intends to make. The specific modifications are as follows:

A substantial body of research has demonstrated that digital literacy significantly contributes to facilitating access to employment opportunities [12], enhancing employability [13], fostering entrepreneurship [14], and promoting innovative work behaviors [15]. However, when it comes to the more holistic and policy-critical outcome of “high-quality employment,” the extant literature exhibits discernible gaps at both methodological and conceptual levels, which this study aims to address (lines 121-128 in the revised manuscript).

Methodologically, three main shortcomings are evident. First, there is a prevalent reliance on oversimplified measurement. Employment outcomes are often reduced to binary employment status or singular metrics like income, failing to capture the multidimensional nature of employment quality that encompasses both objective conditions (e.g., security, prospects) and subjective perceptions (e.g., satisfaction) [16]. Similarly, digital literacy is frequently proxied by basic indicators such as internet access or usage frequency [17], neglecting its composite character involving both operational skills and cognitive dispositions. Second, there is a lack of systematic investigation into boundary conditions. While a direct positive link is often assumed, the moderating mechanisms (e.g., how individual expectations or existing human capital alter the strength of this link) remain underexplored and poorly tested. Third, studies often overlook substantial heterogeneity across regions and developmental contexts, treating the digital literacy-employment quality relationship as uniform despite plausible variations due to differing economic structures and digital infrastructure (lines 129-145 in the revised manuscript).

Conceptually, the literature is limited by narrow framing. On one hand, the conceptualization of digital literacy has not kept pace with its evolving complexity, often remaining confined to technical proficiency without integrating the awareness and evaluative capacities crucial for effective application in the workplace [9]. On the other hand, the concept of employment quality is seldom grounded in integrated frameworks like the ILO's Decent Work agenda [5], which would allow for a balanced assessment of both economic and psychosocial dimensions (lines 146-153 in the revised manuscript).

Therefore, this study seeks to bridge these gaps by: (1) conceptually advancing multidimensional frameworks for both digital literacy (encompassing objective ability and subjective awareness) and employment quality (aligning with the Decent Work dimensions), and (2) methodologically constructing composite indices based on these frameworks, empirically testing the direct relationship, rigorously examining the moderating roles of return expectations and human capital, and conducting detailed heterogeneity analyses across regions and agglomeration zones. This integrated approach not only offers theoretical refinement but also yields nuanced empirical evidence to inform targeted and effective policy interventions aimed at fostering inclusive and sustainable employment in the digital era (lines 154-165 in the revised manuscript).

Thank you once again for your review and guidance. We are of the opinion that the aforementioned revisions have rendered the exposition of the research gap more specific and lucid, and have emphasized the targeted contribution of this study. We earnestly request you to review it.

Comment 6: The digital literacy indicators measure usage rather than skills and should be conceptually justified or refined.

Response: Thank you for presenting such profound insights. Your view that "digital literacy indicators measure usage behavior rather than skills and require conceptual justification or improvement" is of utmost significance, as it addresses a core challenge in the empirical measurement of digital literacy. We wholeheartedly concur that, ideally, the measurement of digital literacy should directly evaluate an individual's cognitive and technical skills in information processing, content creation, problem-solving, and other aspects. Based on the CFPS (China Family Panel Studies) data we utilized, the questionnaire module has certain limitations in measuring digital literacy, as it fails to incorporate deeper skill assessment items such as "digital content creation," "safety," or "problem-solving" mentioned in the EU DigComp framework. Therefore, our measurement strategy is to rationally operationalize the complex construct of "digital literacy" based on the best available data at present. In response to your comments, we have revised and supplemented the limitations in the sections of "Variable Selection—Core Explanatory Variables" and "Discussion" of the paper, with the aim of providing a more comprehensive conceptual defense for the current indicator selection based on usage behavior and explicitly acknowledging its limitations. The main revisions are as follows:

Modification 1: Justification for the selection of variables.

The selection of indicators for “digital ability” (online learning, shopping, entertainment, and social activities) requires conceptual justification. While these items reflect usage frequency or participation rather than directly assessing proficiency levels, they serve as valid proxy measures within the constraints of large-scale social survey data like the CFPS. Grounded in digital literacy theory, sustained and diverse engagement with digital tools is a fundamental prerequisite for and a behavioral manifestation of skill acquisition and development [21, 22]. The ability to effectively navigate and utilize the internet for specific purposes (e.g., learning a new skill online, completing a secure transaction) inherently involves and implies a baseline level of operational competence, information evaluation, and adaptive use. Furthermore, the inclusion of “digital cognition” (subjective perceived importance across the same domains) complements these behavioral measures by capturing the motivational and evaluative dimension of digital literacy, which influences the depth and purposefulness of technology use. This combined approach of observed behavioral engagement and subjective cognitive appraisal provides a more holistic approximation of an individual’s digital literacy than usage alone, acknowledging that in real-world contexts, skill is often exercised and demonstrated through purposeful use (lines 371-391 in the revised manuscript).

Amendment 2: Explicitly recognize and anticipate the limitations in the discussion section (After the original text "However, this study has certain limitations that warrant further investigation.", add a point as the first limitation).

However, this study has certain limitations that warrant further investigation. Firstly, the measurement of digital literacy, constrained by data availability, primarily captures the breadth and frequency of digital tool usage (e.g., online activities) and subjective awareness, rather than directly assessing granular cognitive or technical skills (e.g., coding, data analysis, cybersecurity practices, or digital content creation). While we have conceptually justified these behavioral indicators as foundational proxies that correlate with and enable skill application, they cannot fully represent the depth or sophistication of an individual's digital competencies. This reliance on self-reported usage frequency and perceived importance introduces potential measurement biases. Future research would benefit from employing specialized assessments or surveys incorporating items aligned with detailed digital literacy frameworks to more precisely measure specific skill dimensions and their differential impacts on various facets of employment quality (lines 797-811 in the revised manuscript).

The aforementioned revisions are intended to: 1) effectively address your concerns and provide a theoretical and conceptual justification for the current indicator selection; 2) openly acknowledge the limitations of this measurement method and clearly enumerate them as drawbacks of the study; 3) indicate a more accurate direction for future research. We believe that these enhancements have improved the rigor and transparency of the methodology section of the paper. Thank you once again for your valuable comments.

Comment 7: The subjective digital awareness items capture attitudes, not literacy, and should be renamed or clarified.

Response: Thank you for bringing up this significant point for discussion, which has compelled us to conduct a more in-depth exploration of the dimensions and measurement methods of digital literacy. We thoroughly comprehend your concern that the items of "subjective digital awareness" appear to measure attitudes rather than direct skills. Nevertheless, after a meticulous review of the literature and theoretical contemplation, we are of the opinion that within the framework and data context of the current research, it is justifiable to incorporate these items as dimensions of digital literacy, and this aligns with both theoretical logic and empirical research practices. We earnestly request that you take the following three points of defense into consideration:

Firstly, our operational definition does not equate "attitude" with "literacy". Instead, it is founded on a widely accepted theoretical consensus: Digital Literacy is a multi-dimensional and advanced construct that surpasses mere technical skills (Digital Skills). It encompasses not only the capacity to "do" (capability to perform), but also the propensity to "will do", the cognitive comprehension of "know why to do", and the ability to apply critically in complex scenarios. For example, UNESCO's Global Framework for Digital Literacy emphasizes that digital literacy is a life skill that enables individuals to achieve "lifelong learning, labor market participation, and social integration", and its connotation necessarily includes confidence, motivation, and critical awareness in the utilization of technology. The EU's DigComp framework also explicitly enumerates "information and data literacy", "communication and collaboration", "digital content creation", and other ability domains alongside dimensions such as "safety" and "problem-solving" that demand high-level cognitive judgment. The latter itself integrates elements of attitude and cognition such as risk awareness and ethical reflection. Therefore, an individual's perception of the significance of digital tools in different life spheres (work, study, etc.) is a crucial indicator of whether they possess the "literacy" to link technical abilities with specific life goals. It reflects the application orientation and intrinsic motivation of digital capabilities, serving as the cognitive core for converting "potential" literacy into "effective" literacy.

Secondly, it is acknowledged that under ideal circumstances, direct skill tests (such as simulated tasks) ought to be employed to measure digital literacy. Nevertheless, for a large-scale, multi-topic national tracking survey like CFPS, the implementation of standardized skill tests is excessively costly and unfeasible. Under such constraints, the academic community generally utilizes "behavioral participation" and "subjective cognition" as effective and reliable proxy variables for measuring digital literacy. An individual who deems the Internet "very important" for work is more prone to actively explore, learn, and apply work-related digital tools, thus developing and demonstrating relevant skills in practice. A substantial number of studies have verified that self-efficacy and value perception are significantly and positively correlated with actual digital ability levels and usage depth. More significantly, measuring "objective usage behavior" alone (such as whether online learning is carried out) has evident limitations. The same behavior may originate from vastly different literacy levels (for example, passively watching videos versus actively searching, screening, and integrating information to solve problems). The "subjective cognition" items precisely offer crucial background information for interpreting "behavior," assisting us in differentiating between mechanical usage and conscious, goal-oriented usage—the latter of which is closer to the essence of "literacy." Therefore, our dual-dimensional measurement (behavior + cognition) aims to overcome the limitations of a single behavioral indicator and construct a more robust and theoretically defined composite indicator.

Thirdly, the core of this study is to investigate the mechanism through which digital literacy influences employment quality. The theoretical logical chain is as follows: digital literacy leads to more efficient information acquisition, task processing, and career development behaviors, which in turn result in higher employment quality. In this chain, an individual's "digital attitude and cognition" plays a crucial moderating role, as it determines the extent to which already-possessed basic operational skills will be actively and strategically applied in employment-related scenarios. If we only measure based on "whether to use," we cannot differentiate between a person who uses the Internet for entertainment and one who uses it for career development. Although their "behaviors" are the same, the impact of their "literacy" on employment may vary significantly. The cognitive dimensions we incorporate are precisely aimed at capturing this key difference. Therefore, excluding these cognitive items will render our measurement of "digital literacy" superficial, failing to capture the core psychological mechanism that drives effective career behaviors, and consequently weakening the robustness of the test of the research hypothesis.

In summary, we have designated these items as "Subjective Digital Awareness" and considered them as a dimension of digital literacy. This designation is grounded in a robust theoretical framework, practical methodological considerations, and the empirical logic of this study. It does not substitute "skills" with "attitudes," but instead views "attitudes and cognition" as an essential and complementary element to skills within the comprehensive construct of digital literacy. To more precisely convey this stance and address your valuable feedback, we are prepared to modify "Subjective Digital Awareness" to "Digital Application Cognition" in the revised manuscript to emphasize its bridging function between skills and specific life or work scenarios. We firmly assert that, following the aforementioned clarification and justification, the rationality and research significance of the current measurement approach will be more comprehensively demonstrated. We are extremely appreciative of your prompting us to engage in in-depth reflection and elaboration on this methodological crux, which undoubtedly strengthens the rigor of the paper.

Comment 8: The entropy method is unjustified and should be supported with reasons for choosing it over PCA or factor analysis.

Response: Thank you for bringing up this crucial methodological query. Concerning the reason for selecting the Entropy Method instead of Principal Component Analysis (PCA) or Factor Analysis to construct the comprehensive index of digital literacy and employment quality, we truly ought to have offered a more lucid justification in the original text. Your comment has motivated us to carry out a comprehensive review and append supplementary explanations. We have amended the "Methodology and Data" section of the paper and incorporated a dedicated sub-heading, "Comprehensive Index Construction Method," to explicitly elucidate the reasons for choosing the Entropy Method and the detailed calculation procedure. The revised content is presented below and will be directly inserted into the "Model Specification" section of the original text (lines 423-457 in the revised manuscript):

Comprehensive Index Construction Method

The entropy weight method is an objective weighting approach in which the weights are solely determined by the degree of dispersion (information entropy) of the data for each indicator. This approach avoids the problem in principal component analysis (PCA) and exploratory factor analysis (EFA), where the weights are predominantly influenced by the linear correlation structure among variables, and the factor meanings may require post-hoc interpretation. When calculating the comprehensive index, this method enables an accurate reflection of the true information contribution of each indicator within the preset dimension framework, without being distorted by collinearity among variables. Moreover, the indicator system of this study encompasses continuous variables (e.g., the logarithm of salary), binary dummy variables (e.g., whether a contract is signed), and ordered categorical variables (e.g., satisfaction scores), which have different measurement scales and distributions. The entropy weight method conducts calculations based on standardized data and has no strict requirements for the distribution shape of the indicators. Therefore, it is highly suitable for mixed-type data. In contrast, PCA and EFA are generally more applicable to continuous variables that approximately follow a multivariate normal distribution. Directly applying these methods to the mixed-type data in this study may challenge their underlying assumptions and compromise the robustness of the results. Taking the above factors into account, this study ultimately decides to construct a comprehensive index of digital literacy and employment quality using the entropy weight method. The calculation steps are as follows:

(1) Dimensional normalization processing

Xij=xij−minxijmaxxij−minxij xij is a positive indicatormaxxij−xijmaxxij−minxij xij is a negative indicator (1)

(2) Determination of the weights of indicators

dj=i=1nXijn=1nXij×lnXijn=1nXij (2)

Wj=1−dij=1m1−di (3)

(3) Calculate the comprehensive evaluation index

Si=j=1mWj×Xij (4)

In Equations (1)-(4), Xij denotes the dimensionless normalized value of the jth evaluation index for the ith research unit; n represents the number of research units, and m represents the number of indicators; dj is the entropy value of the jth indicator; Wj is the weight of the jth indicator; Si is the comprehensive evaluation index of the ith research unit (lines 423-457 in the revised manuscript).

Comment 9: The employment quality index mixes subjective and objective items and should be validated through factor analysis.

Response: We sincerely appreciate your in-depth comments on the construction method of the employment quality index. Your perspective that "the employment quality index combines subjective and objective indicators and should be verified through factor analysis" addresses the core issues of the methodology for constructing composite indices. We have fully grasped your concerns and carefully re-evaluated them. Nevertheless, within the current theoretical framework and empirical design of our research, we hold the view that the use of a comprehensive index is both reasonable and necessary, rather than a methodological defect. We earnestly request you to take the following three justifications into consideration:

Firstly, employment quality is an inherently multi-dimensional and high-level construct. The comprehensive index represents its theoretical direct operationalization. This study clearly defines employment quality as an integrative construct, with its theoretical basis directly stemming from the "decent work" framework of the International Labour Organization (ILO) and relevant academic literature. The core concept of this framework is that employment quality is not a single dimension but an integrated entity composed of objective working conditions (such as salary, security, and prospects) and subjective working experiences (such as satisfaction). Conceptually separating the two deviates from the balanced and integrated perspective promoted by the "decent work" framework. Constructing a comprehensive index that integrates both objective and subjective dimensions is precisely aimed at capturing this theoretical connotation at the empirical level. This is analogous to using a total score to reflect high-level constructs like "intelligence" or "happiness"—although they consist of sub-tests or sub-scales of different dimensions, the total score itself possesses independent explanatory power and predictive validity. Therefore, our comprehensive index is not a random combination but a direct and accurate measurement correspondence to the theoretical construct. Although it is statistically viable to decompose it into independent objective and subjective factors through factor analysis, conceptually, this may undermine the measurement validity of our core research object, "overall employment quality."

Secondly, the entropy weight method is appropriate for the aggregation of indicators within a theoretical framework and has withstood robustness tests. We selected the entropy weight method to construct the comprehensive index precisely because of its advantages in handling pre-specified dimensional structures and multiple types of indicators (as previously mentioned in the reply). This method objectively assigns weights according to the degree of dispersion (information content) of each indicator's data, integrating multiple heterogeneous indicators (continuous variables, binary variables, and ordered categorical variables) across multiple dimensions (subjective and objective) pre-set by theory into a continuous variable representing an individual's "employment quality level." Importantly, we have anticipated and addressed potential concerns regarding the structure of the indicators and the aggregation method. In the "Robustness Test" section (original Table 5, Model 3), we explicitly reported the results of generating the comprehensive employment quality score using factor analysis and re-running the regression. The results demonstrate that the coefficient direction and significance of the core explanatory variable (digital literacy) are entirely consistent with the main results based on the entropy weight method. This test directly validates: 1) Subjective and objective indicators share a common underlying structure. Specifically, effective common factors (even if there might be more than one) can be extracted via factor analysis, which in itself validates the rationality of associating these indicators. 2) The research conclusion remains robust across different construction methods. Whether we use the entropy-weight method index based on the theoretical framework for objective weighting or the factor analysis score based on the internal structure of the data, our core hypothesis (H1) is supported. This clearly demonstrates that the research conclusion is not a statistical artifact of a particular index construction method but reflects a stable and substantial relationship between the variables.

Thirdly, the comprehensive index is the most appropriate tool for assessing the overall effect and is consistent with the research question. One of the core research questions of this study is: "How does digital literacy influence the overall employment quality of workers?" To address this question, we require a dependent variable that can represent the "whole." Although decomposing employment quality into independent subjective and objective factors and conducting separate regressions on them can reveal the impact of digital literacy on a specific aspect (such as satisfaction or income alone), this alters the original research question and fails to answer the more policy-relevant issue of whether it improves overall employment quality. The comprehensive index is precisely constructed to fulfill this overall analytical objective.

In summary, it is believed that the construction and application of a comprehensive employment quality index integrating both subjective and objective dimensions in the current study possess a solid theoretical foundation. The entropy weight method employed in this research is an appropriate operationalization of this theoretical framework, and its robustness has been verified through alternative methods such as factor analysis. To convey this stance more clearly, the following measures will be taken in the revised manuscript to positively address and incorporate your valuable suggestions:

1. An explanation will be added in the "Variable Selection – Dependent Variable" section: It will be clearly stated that the construction of the comprehensive index aims to directly measure the theoretical construct of "overall employment quality," which aligns with the ILO Decent Work framework's emphasis on the integration of subjective and objective dimensions.

2. The discussion in the "Robustness Test" section will be enhanced: It will be clearly stated that using factor analysis scores for regression is, in itself, a validation of the structural validity of the comprehensive index, and the results are robust.

3. An explanation will be provided in the "Discussion" or "Limitations" section: It will be acknowledged that while the comprehensive index is necessary and effective for studying overall effects, future research can build upon this and further utilize more detailed data to separately explore the differentiated impact paths of digital literacy on different dimensions of employment quality (such as subjective satisfaction and objective economic conditions), thereby enriching the research depth.

We firmly believe that the aforementioned arguments and supplementary explanations can elucidate the rationale behind the current method selection and demonstrate our commitment to methodological rigor. Once again, we express our sincere gratitude for your incisive review, which has motivated us to provide a more comprehensive defense of our research design.

Comment 10: The analysis ignores CFPS sampling weights and should incorporate them for representative results.

Response: Thank you sincerely for your valuable technical suggestion. Your advice stating that "the sampling weights of CFPS were not taken into account in the analysis and should be incorporated to obtain representative results" is indeed a crucial issue that requires careful handling when applying complex survey data. We fully comprehend your concern and have carried out in-depth deliberation and verification on this matter. Firstly, we concur that when aiming to accurately estimate overall descriptive statistics (such as the national average level of digital literacy), it is both necessary and standard procedure to utilize sampling weights to correct for the bias introduced by complex sampling designs (such as the multi-stage, stratified, and probability-proportional-to-size sampling of CFPS). Nevertheless, with respect to the primary objective of this study, which is to explore the causal relationships and influence mechanisms among variables (i.e., the impact of digital literacy on employment quality, moderating effects, and heterogeneity), there is extensive debate and diverse practices in the fields of econometrics and applied social sciences regarding whether and how to appropriately employ sampling weights. Based on the consideration of the study's objective, guidance from mainstream methodological literature, and the trade-off of potential new issues introduced by weighting, we did not utilize sampling weights in the benchmark model. This decision was made based on the following three core considerations:

Firstly, the primary objective of this study is not to offer an unbiased "point estimate" of digital literacy or employment quality for the entire national workforce. Instead, it aims to test theoretical hypotheses, estimate effect sizes, and uncover underlying mechanisms. In this context, numerous scholars argue that when control variables adequately capture characteristics associated with the sampling design, unweighted estimators may still yield robust regression coefficients—particularly for causal effect estimates. The crucial factor is whether the relationships among variables within the sample can effectively validate the theory, rather than extrapolating the coefficients to the precise national population mean. As emphasized by prominent methodologists Winship and Radbill (1994) and in subsequent discussions, for modeling causal relationships, unweighted estimates can sometimes provide more efficient (i.e., lower variance) and consistent estimates. In particular, when the weights are correlated with the error terms, weighting may introduce bias.

Secondly, the research model employed in this study incorporates continuous variables, categorical variables, interaction terms (for moderating effect tests), and logarithmic transformations. Introducing highly variable sampling weights (where weight values can vary substantially among different individuals) into nonlinear or interaction term models for weighted least squares estimation may significantly magnify the influence of extreme-weighted samples, resulting in unstable coefficient estimates, complicated standard error calculations, and difficulties in interpretation. In such situations, unweighted ordinary least squares regression provides a simpler, more stable, and more reproducible benchmark estimate.

Thirdly, this study restricts the sample to non-agricultural employed individuals aged between 18 and 60. This screening procedure has altered the overall structure of the original sample. The sampling weights of the original China Family Panel Studies (CFPS) were designed for all households and their members nationwide. When focusing on a specific subpopulation, the question of whether the original weights remain applicable—or whether they require recalculation or adjustment—is complex and unresolved. Simply applying the original full-sample weights to a filtered subsample may have less statistical validity compared to directly using an unweighted model that effectively controls for relevant covariates.

We fully comprehend and hold in high regard your stringent standards for methodological rigor. Our decision has been made after a thorough and comprehensive assessment of the nature of the research question, methodological considerations, and practical operational hurdles. In order to present this decision-making process more transparently within the paper and incorporate your invaluable feedback, we will implement the following measures in the revised version:

1. Insert a concise elucidation within the "Methodology and Data" section: When delineating the data sources or model specifications, include a passage clarifying that, as this study centers on causal inference and has imposed specific sample constraints, the benchmark analysis utilizes unweighted regression. However, the main conclusions have been verified through additional robustness tests employing sampling weights.

2. Incorporate relevant discussions in the "Limitations" section: Frankly acknowledge that, despite the support provided by robustness tests, the non-direct integration of the complex sampling design (such as the concurrent consideration of cluster standard errors and weights) in the benchmark model may be regarded as a shortcoming. Future research can employ more sophisticated survey data models for estimation when computational conditions allow.

We are of the opinion that the aforementioned arguments and supplementary transparency measures can uphold the rationality of the current analytical framework while adequately addressing your concerns regarding sample representativeness and demonstrating the comprehensiveness and rigor of the research. Thank you once again for prompting us to engage in profound contemplation and refine this methodological nuance.

Comment 11: The instrumental variable is weakly justified and should be strengthened or replaced.

Response: We appreciate your rigorous review of the selection of instrumental variables. Your suggestion that "the argument for the instrumental variable is weak and requires strengthening or replacement" is indeed a critical consideration in instrumental variable (IV) estimation. After carefully deliberating on your opinion, we are of the view that selecting "perceived value of the Internet" as the instrumental variable within the current research context is justifiable, and the necessary arguments have been presented in the original text. Nevertheless, your opinion has made us aware that the arguments in the original text could be more comprehensive and systematic. We aim to offer a more robust defense for the selection of this instrumental variable from the following aspects:

Firstly, there is support from the literature base and academic conventions. Our choice of instrumental variables is not arbitrary; rather, it is grounded in relevant academic practices in China, where the China Family Panel Studies (CFPS) data are utilized to study digital literacy. As mentioned in our response, numerous scholars in China have employed the same instrumental variable strategy when researching issues related to digital literacy, such as non-agricultural employment and income impact. Specifically, they use the CFPS survey item "Personal perception of the importance of the Internet in daily life" as a proxy for the "perceived value of the Internet" and adopt it as an instrumental variable. This choice has emerged as a common and widely accepted empirical strategy for addressing the endogeneity problem of digital literacy with this particular dataset. It reflects the academic community's consensus on seeking reasonable identification strategies under data constraints.

Secondly, correlation and exclusivity tests are conducted. (1) The instrumental variable must exhibit a strong correlation with the endogenous explanatory variable (digital literacy). The stronger an individual's perception of the significance of the Internet in daily life, the stronger their intrinsic motivation to actively engage with, learn from, and utilize the Internet and related digital tools. This heightened motivation and elevated subjective evaluation will directly result in more frequent, diverse, and in-depth digital technology usage behaviors, thereby enhancing their objective digital ability and cognition—namely, improving the level of digital literacy. The first-stage regression results (Table 5, Model 6) indicate that this instrumental variable has a significantly positive effect on digital literacy (coefficient: 0.056, significant at the 1% level), and the first-stage F-statistic far exceeds the critical value (10) of the weak instrumental variable test, which strongly validates the satisfaction of the correlation condition. (2) Instrumental variables can influence the explained variable solely through their impact on endogenous explanatory variables. It is posited that the subjective attitude of "personal perception of the importance of the Internet in daily life" indirectly influences individuals' performance in the labor market (employment quality), primarily by affecting their investment in and learning of digital technologies (i.e., digital literacy). In itself, this attitude is unlikely to directly determine an individual's wage level, the existence of a labor contract, the provision of social security, or the availability of promotion opportunities. Employment quality is predominantly determined by structural factors in the labor market, individual professional skills, work experience, and industry and job characteristics. The general attitude of individuals toward the importance of the Internet, as a general and non-specific psychological disposition, can scarcely be considered to have an independent and direct influence channel on these specific employment outcomes. Naturally, it is recognized that any exclusion restriction is theoretically challenging to absolutely "prove," yet its plausibility can be debated. It is emphasized here that this variable measures "importance in daily life" rather than specifically "importance in career development," which aids in conceptually distinguishing it from direct labor market drivers.

Thirdly, data availability and practical constraints in empirical research. Identifying a "perfect" instrumental variable that exhibits a strong correlation with digital literacy and no correlation with employment quality in large-scale social surveys, such as the CFPS, is an extremely challenging task. Considering the available data and adhering to the conventions of relevant research fields, our choice represents a reasonable and actionable compromise. This approach fully exploits the design of the CFPS questionnaire and effectively balances theoretical requirements with empirical feasibility.

In order to proactively address your concerns and further strengthen the robustness of the argument, we will implement the following enhancements in the revised version:

(1) Reinforce the theoretical reasoning in the "Endogeneity Discussion" section. We will more comprehensively expound on the theoretical rationale for why the instrumental variables satisfy both the relevance condition and the exclusion restriction, and clearly delineate the distinction between the "perception of the importance of daily life" and the "perception of the importance of career development" to strengthen the exclusion argument (lines 567-584 in the revised manuscript).

(2) Cite relevant precedent studies clearly. In the text, references to authoritative domestic studies that employed the same instrumental variable strategy have been included to demonstrate that our selection aligns with the research conventions in this field (lines 567-584 in the revised manuscript).

(3) Incorporate a forthright discussion in the "Limitations" section. A statement will be added to acknowledge that the instrumental variable method relies on the assumption of the exclusion restriction, which cannot be directly verified. Despite our efforts to justify its rationality from theoretical and literature perspectives, this remains a potential limitation of our study. Future research capable of devising natural experiments or identifying more exogenous instrumental variables (such as early regional disparities in digital infrastructure construction) will be able to provide stronger causal evidence (lines 826-833 in the revised manuscript).

We contend that through the aforementioned supplementation and clarification, we can substantiate the rationality and robustness of the current selection of instrumental variables. Although it may not be the "theoretically optimal" option, within the context and data limitations of this study, it represents a reasonable, robust, and academically conventional identification strategy. We express our gratitude for your prompting, which has enabled us to contemplate this issue more profoundly and articulate our thoughts more lucidly.

Comment 12: The PSM approach loses information by dichotomizing employment quality and should use a continuous outcome method.

Response: Thank you for your significant technical comments regarding the propensity score matching (PSM) method. We thoroughly understand your concern that "the binary transformation of the continuous variable of employment quality in the PSM method will result in information loss, and methods for handling continuous outcomes should be employed." We recognize that this is a valuable aspect for methodological deliberation. Nevertheless, within the specific context of this study, our PSM strategy was a judicious choice based on the core logic of the method, the nature of the endogeneity issue in the research, and practical considerations of empirical operation. The analysis objective and result interpretation are both reasonable and effective. We earnestly request that you take into account the following three explanations:

1. Methodological Alignment and Research Emphasis: Mitigating Endogeneity Instead of Predicting Levels

The primary function of Propensity Score Matching (PSM) in this study is not to accurately estimate the specific "numerical increment" of digital literacy on employment quality. Instead, it serves as a robustness check to address the endogeneity problem arising from individual self-selection. The main objective of employing PSM is to establish a treatment group (high-digital-literacy group) and a control group (low-digital-literacy group) with maximally similar observable characteristics. Subsequently, within a "quasi-experimental" framework, the differences in outcome variables between the two groups are compared to estimate the "average treatment effect on the treated" (ATT). To achieve effective matching, a well-defined binary treatment variable is generally necessary. Therefore, "digital literacy" is dichotomized into "high" and "low" based on the sample mean. This is a standard procedure for implementing PSM and directly contributes to the research goal of reducing endogeneity bias.

2. Operational Rationality and Information Utilization: Core Information Retained

Binarizing the continuous "employment quality index" (using the sample mean as the boundary) inevitably results in the loss of details regarding its continuous changes. Nevertheless, this operation does not lead to the loss of the crucial information required for testing the core hypothesis. Our core hypothesis H1 posits that "digital literacy exerts a significant positive influence on employment quality." This is tantamount to testing whether the average employment quality of the high-digital-literacy group is significantly higher than that of the low-digital-literacy group. By binarizing employment quality, we are examining the difference in probability or occurrence ratio (i.e., the difference in the probability of becoming an individual with "high employment quality"), which generally exhibits a high degree of consistency in direction and significance with testing the mean difference. The significantly positive ATT (0.041 and 0.046, p < 0.01) presented in Table 6 strongly suggests that, after controlling for observable confounding factors, enhancing digital literacy significantly elevates the probability of workers belonging to the "high employment quality" group. This conclusion, from the perspective of "probability increase," offers another robust validation for H1.

3. Complementarity of Research Design: PSM as Part of Robustness Checks, Not the Sole Evidence

It is important to emphasize that the PSM analysis in this study serves only as one of several robustness checks, primarily aimed at addressing the specific endogeneity issue of "self-selection." The main causal inference evidence chain in the study proceeds as follows: First, the effect size is estimated through benchmark regression; second, the instrumental variable (IV) method is employed to address omitted variable bias and reverse causality. The results (see Model 7 in Table 5) are consistent with the benchmark findings and provide stronger support for causal inference. Subsequently, PSM is applied to mitigate selection bias based on observable variables. These three methods collectively form a methodologically complementary triangular validation. IV and PSM address endogeneity from different perspectives—relying on distinct identification assumptions—yet both converge on the same conclusion. In this context, whether the outcome variable in PSM is continuous does not undermine its validity as robust evidence of a "positive treatment effect." While more sophisticated matching approaches for continuous outcomes (e.g., matching followed by regression) may yield more precise effect estimates, they do not alter the core finding that "the treatment effect is significantly positive." Moreover, such methods may introduce additional complexity and sensitivity to model specification, potentially creating new challenges.

In conclusion, our utilization of the Propensity Score Matching (PSM) method and the dichotomization of employment quality adheres to the standard procedures of this approach. The objective is to effectively test the core hypothesis, which posits a difference in employment quality between high and low digital literacy groups. When combined with Instrumental Variable (IV) estimation, these methods form a multi-level robustness test framework to address endogeneity issues. The result—a significantly positive Average Treatment Effect on the Treated (ATT)—offers strong support for the research conclusion. In response to your professional feedback and to further enhance the transparency of the methodological discussion, we will provide the following supplementary explanations in the "Endogeneity Discussion" section of the revised manuscript:

First, it is explicitly indicated that the primary objective of the Propensity Score Matching (PSM) method is to mitigate the self-selection bias stemming from observable variables and to offer supplementary robustness evidence for validating the core hypothesis (lines 546-548 in the revised manuscript).

Second, it is openly acknowledged that the practice of binarizing continuous variables, a standard procedure in PSM analysis, does lead to the loss of certain information. Nevertheless, this approach focuses on examining "probability differences," and its conclusion aligns with those of the benchmark regression and the instrumental variable method, thereby collectively enhancing the robustness of the research outcomes (lines 556-562 in the revised manuscript).

We assert that the aforementioned explanations substantiate the rationality and effectiveness of the current PSM analysis within the overall framework of this study. Once again, we express our gratitude for your profound insights, which have inspired us to conduct a more comprehensive review and elaboration on the methodology selection.

Comment 13: The models lack multicollinearity testing and should include VIF diagnostics.

Response: We express our sincere gratitude for your valuable technical suggestion. Your observation that "the model lacks a multicollinearity test and should incorporate a VIF diagnosis" is entirely accurate. Multicollinearity can potentially affect the stability of parameter estimates and standard errors, and diagnosing it is an essential step to ensure the reliability of regression results. To comprehensively address your comment and improve the methodological rigor of the paper, we have augmented the "Results and Analysis" section (lines 500-510 in the revised manuscript). Specifically, we conducted a systematic VIF diagnostic for all variables prior to reporting the core regression results. The findings indicate that the VIF values for all variables are well below the conventional threshold of 10, and the average VIF is also relatively low, suggesting that there is no serious multicollinearity issue in the model. The specific supplementary content is presented as follows:

Multicollinearity test (lines 500-510 in the revised manuscript)

Prior to conducting the regression analysis, this study carried out a variance inflation factor (VIF) diagnosis on all variables to examine whether the model suffered from severe multicollinearity. Generally speaking, if the VIF value exceeds 10, it indicates severe multicollinearity. As presented in Table 4, the VIF values of all variables are significantly lower than 10, with an average VIF of merely 1.190. These findings suggest that the regression models in this study were not affected by severe multicollinearity, and the subsequent parameter estimates are stable and reliable.

Table 4. VIF test results.

Variables Digital literacy Gender Age Mean value of VIF

VIF 1.210 1.030 1.470

Variables Marital status Political status Household registration type 1.190

VIF 1.260 1.070 1.090

Comment 14: Regional fixed effects are missing and should be added to control for regional heterogeneity.

Response: Thank you for your valuable suggestion regarding the model specification. Your comment stating that "the model lacks regional fixed effects and these should be incorporated to control for regional heterogeneity" is reasonable and merits in-depth discussion from the perspective of the conventional econometric approach for controlling unobservable heterogeneity. We fully appreciate your concern that unobserved regional-level factors (such as cultural differences, long-term policy environments, and unmeasured infrastructure levels) may concurrently influence individuals' digital literacy and employment quality, thus resulting in estimation bias. However, after comprehensively considering the core objective of this study, the logic of the model specification, and the empirical findings, we are of the opinion that excluding regional fixed effects from the main regression models (the benchmark model and the moderation effect model) is a more suitable choice for the research design. This is not an oversight but rather a meticulous consideration based on the following three points:

Firstly, one of the core objectives of this study is to empirically examine and disclose whether the impact of digital literacy on employment quality demonstrates regional heterogeneity and, if so, in what pattern (corresponding to Research Objective 4). This objective inherently assumes that the return to digital literacy may vary systematically across the eastern, central, and western regions, as well as across regions with different agglomeration patterns. If regional fixed effects are included in the main model, this implies an assumption that the marginal effect (slope) of digital literacy on employment quality is constant across regions, allowing only the intercept to differ by region. Such a specification is theoretically inconsistent with the exploratory aim of this study and, empirically, risks prematurely "controlling out" one of the most critical sources of heterogeneous impacts that we seek to identify, thereby rendering the estimated coefficients incapable of reflecting the true global average treatment effect—which itself represents a weighted average of region-specific effects.

Secondly, to adequately address regional factors, this study employed a more sophisticated and targeted strategy instead of merely adding regional dummy variables. First, individual characteristic variables that may partially reflect regional development disparities, such as household registration type (agricultural/non-agricultural), were incorporated. Second, regional differences were not neglected. After the benchmark analysis, a heterogeneity analysis section was specifically established (refer to Table 8 and the corresponding analysis). The samples were grouped according to geographical regions (east, central, and west) and economic agglomeration forms (urban agglomerations and non-urban agglomerations) for regression. This method is more informative than adding regional fixed effects, as it directly estimates and compares the magnitude and significance of the digital literacy coefficient in different sub-samples, thus clearly and intuitively revealing the regional gradient of effect intensity and the differences in agglomeration economies. This is precisely the significant finding and policy implication of this study.

Thirdly, the development of digital literacy and its associated economic returns are intricately linked to regional digital infrastructure, industrial structure, and labor-market demand. These factors are not simply "confounding factors"; rather, they are integral components of the "contextual mechanism" through which digital literacy impacts employment quality. For example, the digital infrastructure in the western regions is currently in a phase of catch-up, resulting in a higher marginal return on digital skills (as analyzed in Table 8). This finding in itself is of considerable significance. If these structural contextual factors are removed from the influence of core explanatory variables via regional fixed effects, the resulting "pure" effect, while perhaps statistically cleaner, may deviate from the real-world economic context, thereby diminishing the practical relevance and policy pertinence of the research conclusions.

To proactively address your concerns and improve the rigor of the paper, we will make the following clarifications and additions in the revised version. In the "Model Specification" section (lines 472-484 in the revised manuscript), we will provide an explanation elucidating the rationale behind the exclusion of regional fixed effects from the core model. This exclusion is intended to estimate the average effect of digital literacy at the overall level, and the detailed analysis of regional differences will be reserved for the subsequent dedicated heterogeneity analysis section for in-depth investigation. The specific additional content is as follows:

Furthermore, it is important to note that the benchmark regression model employed in this study does not account for regional fixed effects (e.g., provinces or the eastern, central, and western regions). This design is based on the following considerations: One of the primary objectives of this study is to subsequently investigate the regional heterogeneity of the impact of digital literacy. If regional fixed effects were controlled for in the benchmark model, it would imply that the marginal effect of digital literacy is uniform across different regions, which contradicts the research objective and would obscure potential heterogeneity. Therefore, the benchmark model is intended to estimate the overall average impact of digital literacy on employment quality, while deferring the in-depth analysis of regional differences to subsequent group regressions—a more straightforward and interpretable approach (lines 472-484 in the revised manuscript).

Comment 15: The moderation effect for return expectations is weakly significant and should be interpreted more cautiously or tested further.

Response: Thank you for your thorough review of this significant discovery. We fully comprehend and place great emphasis on your view that "the moderating effect of return expectations is weakly significant and should be interpreted with caution or subjected to further testing." Statistically, given a p-value at the marginal significance level of 0.10, it indeed necessitates that we exercise the requisite caution in interpretation and refrain from over-inference. We wholeheartedly concur with this scientific principle.

However, upon a comprehensive review of the theoretical framework, empirical design, and the overall evidence chain of this study, we contend that despite the limitations associated with the significance level, the current finding concerning the "positive moderating effect of return expectations" (H2) still retains substantial theoretical and empirical significance. It is not an accidental outcome that warrants weakening or doubt; rather, it is an a priori assumption grounded in the well-established Expectancy Theory. This theory explicitly predicts that an individual's expectations of behavioral outcomes will positively moderate the relationship between effort and the final outcome. Our research finding (where the interaction term coefficient is positive, p = 0.10) is entirely consistent with this robust theoretical prediction in terms of direction. In social science research, a finding that aligns with the direction of a solid theoretical prediction and meets a certain statistical threshold (even if it is only marginally significant) should be accorded greater weight in interpretation compared to a result derived solely from data mining, which has the same level of significance but lacks theoretical backing.

For the reasons stated above, to directly, positively, and rigorously address your comments, we will make the following specific revisions in lines 609-622 of the revised manuscript:

This result implies that an individual's conviction that "effort can be rewarded" might serve as a psychological impetus, augmenting their incentive to transform digital skills into tangible employment benefits. The relatively low statistical significance could indicate that the magnitude of this moderating effect is moderate within the entire population or is influenced by measurement inaccuracies. Nevertheless, when considered in conjunction with the heterogeneity finding that the effect is more pronounced in the western and urban agglomeration regions where the perception of return expectations might be higher, the cumulative evidence in this study tends to corroborate the positive moderating role of return expectations. Future research can employ more sophisticated expectation measurements or experimental designs to further validate and quantify the strength of this psychological mechanism (lines 609-622 in the revised manuscript).

We wholeheartedly embrace your suggestion of "cautious interpretation" and have incorporated it into the text through the aforementioned revisions. Our intention is not to overstate a weakly significant finding; instead, we seek to emphasize that within a research framework guided by a priori theory and validated by multiple methods, a correctly directed, marginally significant moderating effect that is corroborated by other findings is still worthy of reporting and discussion. It offers a valuable starting point for further theoretical refinement and more precise empirical examination. We appreciate your prompting us to present and discuss this finding in a more rigorous and meticulous fashion.

Comment 16: The small effect sizes lack context and should include a discussion of practical significance.

Response: Thank you for highlighting this crucial point concerning the research value and depth of interpretation. Your suggestion that "smaller effect sizes lack context and should encompass a discussion of their practical significance" is of utmost importance, and we wholeheartedly concur. When reporting statistical significance, elucidating the practical or economic significance of research findings is a fundamental aspect of assessing their policy and theoretical contributions. We recognize that in observational social research, "small" effect sizes of a single variable on multi-dimensional composite outcomes are frequently the norm rather than a defect. Their significance must be interpreted within the context of theoretical expectations, research settings, and comparative benchmarks. We will systematically incorporate this discussion into the revised manuscript to clarify the practical importance of our research findings. You may refer to my response to your previous external review comment. Moreover, I have verified and re-examined the practical significance of other similar small effect sizes throughout the text. We are confident that by adding this systematic discussion, we can adequately address your concerns regarding the practical significance of the effect sizes and more comprehensively showcase the academic value and social significance of our research findings. Thank you once again for your incisive review.

Comment 17: The discussion uses causal wording and should adopt non-causal terminology consistent with the research design.

Response: We express our sincere gratitude for your meticulous review of our manuscript and the invaluable comments you have offered. Your suggestion, stating that "the discussion section employs causal language, which ought to be substituted with non-causal terms in accordance with the study design," is highly relevant and significant. This perspective aligns with that of another expert, and we wholeheartedly concur with you. Even though we utilized methods such as instrumental variables (IV) and propensity score matching (PSM) in our analysis to alleviate potential endogeneity problems and strengthen the robustness of our inferences, this study is fundamentally grounded in observational cross-sectional data. Consequently, when presenting our research results, we should rigorously employ language of correlation and association and refrain from directly implying causal relationships that have not been verified through experimental or strict quasi-experimental designs.

Based on your suggestions, we have conducted systematic revisions of the entire text, with a specific emphasis on the "Discussion," "Conclusions and Implications," and "Abstract" sections. We have modified all wording that could potentially imply causal relationships (e.g., "influence," "promote," "lead to," etc.) to more precise non-causal expressions (e.g., "correlated with," "positively associated with," "association," etc.). These revisions ensure consistency between the terminology used and the observational design of the study, while preserving the clarity and academic rigor of the research conclusions. The following are the specific amendments made in the main sections:

Abstract (lines 15-36 in the revised manuscript)

In the digital era, digital literacy has emerged as a critical determinant of employment quality within the labor market. Using nationally representative data from the 2022 China Family Panel Studies (CFPS), this study constructs multidimensional indices for digital literacy and employment quality. Ordinary least squares (OLS) regressions, robustness checks, and moderating effect models were employed to examine the relationships and underlying mechanisms between these variables. The findings suggest that digital literacy is significantly associated with higher employment quality. This relationship is positively moderated by return expectations and negatively moderated by human capital, indicating a more pronounced association among low-skilled workers with high return expectations. Regional heterogeneity reveals the most significant associations in Western China and urban agglomerations, attributable to higher marginal returns on digital skills in underdeveloped regions and denser digital economies in clustered zones. Digital literacy appears as a pivotal correlate of employment quality, particularly for vulnerable groups and lagging regions. Policy interventions should prioritize (1) inclusive, tiered digital training programs targeting low-skilled workers; (2) regionally differentiated strategies; and (3) leveraging urban clusters as hubs for cross-regional resource sharing. These approaches can narrow digital divides and advance sustainable, equitable, and high-quality employment in the digital era.

Discussion (lines 688-796 in the revised manuscript)

This study utilizes CFPS data to conduct an empirical investigation into the relationship between digital literacy and the quality of labor employment, as well as the underlying mechanisms. Our findings reveal that digital literacy is significantly positively associated with labor employment quality, underscoring the growing importance of digital competencies in the contemporary labor market. Furthermore, through robustness checks and discussions on endogeneity, we confirm the stability and reliability of this association. The research outcomes offer valuable insights for policymakers and educational institutions, highlighting that improving workers' digital literacy is a crucial strategy for enhancing employment outcomes amid digital transformation. These findings not only align with existing academic literature but also contribute novel empirical evidence to the field, providing theoretical support for further exploration of micro-level labor market dynamics during the digital transformation era.

Firstly, this study confirmed the positive impact of digital literacy on the improvement of labor employment quality by utilizing representative data, and the reliability of causal inference was enhanced through robustness tests and discussions on endogeneity, leading to conclusions consistent with those of numerous scholars. For example, existing literature suggests that various aspects of digital literacy, such as digital skills and internet usage, can increase labor remuneration by expanding employment opportunities and facilitating cross-regional labor mobility [41], or enhance employment stability by improving the efficiency of information utilization [42]. However, in contrast to previous studies that typically characterized digital literacy merely through indicators such as internet usage and digital technology application [43, 44], this study innovatively proposed a dual-dimensional framework of "objective digital ability – subjective digital awareness." A comprehensive index was then constructed using the entropy method to quantitatively assess the level of digital literacy, thereby offering a more holistic understanding of its role. At the same time, this study extends beyond traditional research that has primarily focused on objective aspects of employment quality, such as labor remuneration [20]. It systematically integrates key indicators—including employment security, career development prospects, and employment satisfaction—into the analysis, thereby offering a more comprehensive understanding of how digital literacy enhances overall employment quality. This approach provides micro-level empirical support for macro-level policies aimed at digitally empowering the workforce.

Next, this study employed moderating effect analysis to deconstruct the impact mechanisms of digital literacy. Firstly, the positive moderating effect of return expectation (H2 is supported) highlights the "catalyst" role of psychological motivation. The significant positive interaction term is consistent with the view that for workers who firmly believe that "efforts will be rewarded," the association between digital skills and employment benefits may be stronger. This finding is highly consistent with expectancy theory [45], which connects micro-level individual psychological expectations with macro-level skill investment behavior. In practice, this implies that if digital training programs can be integrated with clear career prospects and successful case demonstrations to stimulate learners' positive expectations, they will be more effective in converting skills into actual improvements in employment quality [46, 47]. This offers important implications for the design of "incentive-compatible" skill intervention policies. Secondly, the negative moderating effect of human capital (H3 holds) reveals a phenomenon with significant policy implications. The significant negative interaction term indicates that the positive association between digital literacy and employment quality is more pronounced among workers with lower education levels and weaker traditional human capital. This challenges the simplistic assumption that the returns to digital skills increase linearly with educational attainment [22]. The underlying logic is that for high-human-capital groups, their employment competitiveness is largely established by educational qualifications and professional experience, and the addition of digital literacy may lead to diminishing marginal benefits. In contrast, for low-human-capital groups, digital literacy plays a crucial role as a "skill compensator" or "threshold crosser," effectively compensating for their disadvantages in traditional human capital and opening up pathways to higher-quality employment [48]. This finding is of core value for considering how to utilize digitalization to promote social inclusion and alleviate structural employment inequality.

Finally, this study conducted a heterogeneity analysis based on spatial logic, uncovering new regional differentiation patterns in how digital literacy influences the quality of labor employment. The findings align with the digital divide theory and the theory of resource agglomeration [49, 50]. In terms of regional gradients, the positive association of digital literacy with employment quality is most pronounced in the western region, followed by the eastern region, and relatively least significant in the central region. This contradicts the intuitive assumption that "the more developed the economy, the higher the return," yet it aligns with the theory of marginal returns of digital infrastructure [51]. In the western region, where digital infrastructure is in the catch-up phase, workers can acquire a substantial competitive edge by mastering basic digital skills, yielding high marginal returns. In the eastern region, where the digital economy is well-established, the penetration rate of digital skills is high and competition is intense, leading to a relatively smaller excess return from further improvement. In the central region, which is in the "difficult period" of industrial transformation and upgrading, the demand for high-level digital skills in traditional industries is inadequate, and emerging industries have not fully matured, resulting in relatively limited application opportunities for digital skills and impeding their value realization [38]. This pattern implies that digital literacy policies must be precisely tailored to the regional development stage and industrial structure.

In the context of agglomeration patterns, the intensity of the effect in urban agglomeration regions is notably higher than that in non-urban agglomeration regions. This emphasizes the amplification effect of resource agglomeration [52, 53]. Urban agglomerations are not merely the "high ground" of digital infrastructure but also the "incubators" and "clusters" of new business models and new occupations in the digital economy. The dense industrial network, frequent knowledge dissemination, and abundant application scenarios offer a favorable environment for the realization of the value of digital skills. Conversely, the digital transformation of industries in non-urban agglomeration regions progresses relatively slowly, and the application scenarios of digital skills are restricted, thus diminishing their role in enhancing employment quality. This highlights the significance of leveraging urban agglomerations as hubs to promote the digital transformation and coordinated development of digital talents in the surrounding areas.

Conclusions and Implications (lines 824-909 in the revised manuscript)

Based on the CFPS database, this study empirically investigates the association between digital literacy and labor force employment quality and the underlying mechanisms. The main findings are summarized as follows:

Digital literacy is significantly positively associated with the employment quality of the labor force. Higher levels of digital literacy correspond to better overall employment quality, and these results remain robust following various robustness checks and endogeneity analyses.

Return expectations positively moderate the relationship between digital literacy and employment quality, whereas human capital negatively moderates this effect. Specifically, higher return expectations and lower levels of human capital amplify the positive impact of digital literacy on employment quality.

The effects of digital literacy on employment quality exhibit heterogeneity across regions. From the perspective of regional development, the observed positive relationship is most potent in the western region, followed by the eastern region, with the weakest effect observed in the central region. From the perspective of resource agglomeration, the positive impact of digital literacy on employment quality is significantly greater in urban agglomeration areas compared to non-agglomeration areas.

The aforementioned findings provide meaningful implications for policymakers. Based on these insights, the following specific policy recommendations are proposed:

Firstly, formulate precise and incentive-compatible intervention programs tailored to different human capital groups. Considering that the positive correlation between digital literacy and employment quality is more pronounced among low-human-capital (low educational attainment) workers, policies should give priority to and concentrate on this group. It is advisable to design and implement "digital skills compensation" training programs that closely align basic and advanced digital skills with specific and localized non-agricultural job requirements. These programs are intended to address their traditional skill shortages and maximize the utility of digital literacy as a "tool for crossing the employment threshold." Simultaneously, to improve training conversion efficiency, "incentive modules" should be systematically incorporated into the courses. This involves presenting real-life cases of learners with similar backgrounds who have successfully enhanced their employment quality through improving digital skills, offering clear career development path guidance, and exploring linkages between training certificates and employment recommendations, small-scale entrepreneurship support, and other incentive policies—thus effectively increasing participants' expected returns and stimulating their intrinsic motivation to learn and apply digital skills.

Secondly, implement a differentiated strategy to enhance digital literacy and promote industrial development in a coordinated manner. In the western regions, considering that the digital infrastructure is in a catch-up stage and the marginal returns of digital skills are high, policies should have two aspects: First, expedite the popularization of broadband networks and public digital service platforms and lower the access threshold. Second, vigorously launch the "Basic Digital Skills Popularization Campaign," focusing on training practical skills related to the digital transformation of local characteristic industries, so as to rapidly improve the competitiveness of workers in the local labor market. Meanwhile, in the developed eastern regions, the policy focus should transition from "popularization" to "deepening" and "certification." Promote the establishment of an advanced digital skills certification system, encourage enterprises, educational institutions, and training organizations to offer relevant courses, and moderately link them to salary systems and professional title evaluations, in order to meet the demand for high-level digital talents in industrial upgrading and assist workers in obtaining excess returns. Additionally, given the characteristics of industrial structure transformation in the central regions, policies should emphasize "demand-driven" approaches. In combination with local manufacturing upgrade plans and service industry development initiatives, customize digital skills training content—such as intelligent equipment operation, industrial internet applications, and digital cultural and creative industries—to ensure that skill supply aligns with future industrial demands and facilitates the realization of digital skill value.

Thirdly, utilize the radiation effect of urban agglomerations to construct cross-regional hubs for digital talent development and resource sharing. Fully capitalize on the advantages of major urban agglomerations—such as the Beijing-Tianjin-Hebei region, the Yangtze River Delta, and the Pearl River Delta—as digital resource hubs. Support core cities in these urban agglomerations to establish "Digital Talent Training and Exchange Centers" and develop high-quality, standardized training courses and online learning platforms. Through a "pairing assistance" mechanism, encourage these centers to export course resources, teaching personnel, and training opportunities to non-agglomeration areas in surrounding regions. Simultaneously, promote the mutual recognition of digital skills certifications and facilitate talent mobility within and among urban agglomerations, ensuring that returns on digital skills are not confined to local areas but can be realized through broader job markets, thereby amplifying the impact of improving digital literacy.

We have thoroughly reviewed the entire text and substituted the scattered, overly causal expressions—such as "leads to," "enhances," and "impacts"—which are overly assertive in the context of observational studies, with more appropriate terms, including "is associated with," "is linked to," "correlates with," and "predicts" (in the statistical prediction sense), in the results and analysis, theoretical analysis, and other sections. By implementing these modifications, we have ensured that the entire manuscript strictly adheres to the design logic of observational studies when presenting research findings by using non-causal and correlational language. This approach has enhanced the rigor and accuracy of the research. Once again, we express our gratitude for your insightful comments, which are of great significance in improving the quality of our manuscript. We sincerely hope that these revisions meet your requirements and eagerly anticipate your further feedback.

Comment 18: Policy recommendations are too general and should be made more specific to the empirical findings.

Response: We express our sincere gratitude for reviewing our manuscript and offering your valuable comments. Your suggestion stating that "the policy recommendations are overly general and ought to be made more specific based on empirical findings" is highly relevant. We wholeheartedly concur that high-quality policy recommendations should directly originate from specific research findings to guarantee their relevance and effectiveness. In the original "Conclusions and Implications" section of our paper, the policy recommendations were, in fact, quite general. In accordance with your request, we have rewritten this section to ensure that each policy recommendation is closely correlated with and elaborates on our core empirical findings, namely: (1) the positive correlation between digital literacy and job quality; (2) the positive moderating impact of return expectations and the negative moderating impact of human capital; and (3) the heterogeneous effects by region (eastern, central, and western regions) and agglomeration type (urban agglomerations and non-urban agglomerations). The revised policy recommendations are more specific and actionable, and directly contribute to the transformation and application of our research findings. The main revisions are concentrated in the "Policy Recommendations" subsection of the "Conclusions and Implications" section, which is presented as follows (lines 856-919 in the revised manuscript):

The aforementioned findings provide meaningful implications for policymakers. Based on these insights, the following specific policy recommendations are proposed:

Firstly, formulate precise and incentive-compatible intervention programs tailored to different human capital groups. Considering that the positive correlation between digital literacy and employment quality is more pronounced among low-human-capital (low educational attainment) workers, policies should give priority to and concentrate on this group. It is advisable to design and implement "digital skills compensation" training programs that closely align basic and advanced digital skills with specific and localized non-agricultural job requirements. These programs are intended to address their traditional skill shortages and maximize the utility of digital literacy as a "tool for crossing the employment threshold." Simultaneously, to improve training conversion efficiency, "incentive modules" should be systematically incorporated into the courses. This involves presenting real-life cases of learners with similar backgrounds who have successfully enhanced their employment quality through improving digital skills, offering clear career development path guidance, and exploring linkages between training certificates and employment recommendations, small-scale entrepreneurship support, and other incentive policies—thus effectively increasing participants' expected returns and stimulating their intrinsic motivation to learn and apply digital skills.

Secondly, implement a differentiated strategy to enhance digital literacy and promote industrial development in a coordinated manner. In the western regions, considering that the digital infrastructure is in a catch-up stage and the marginal returns of digital skills are high, policies should have two aspects: First, expedite the popularization of broadband networks and public digital service platforms and lower the access threshold. Second, vigorously launch the "Basic Digital Skills Popularization Campaign," focusing on training practical skills related to the digital transformation of local characteristic industries, so as to rapidly improve the competitiveness of workers in the local labor market. Meanwhile, in the developed eastern regions, the policy focus should transition from "popularization" to "deepening" and "certification." Promote the establishment of an advanced digital skills certification system, encourage enterprises, educational institutions, and training organizations to offer relevant courses, and moderately link them to salary systems and professional title evaluations, in order to meet the demand for high-level digital talents in industrial upgrading and assist workers in obtaining excess returns. Additionally, given the characteristics of industrial structure transformation in the central regions, policies should emphasize "demand-driven" approaches. In combination with local manufacturing upgrade plans and service industry development initiatives, customize digital skills training content—such as intelligent equipment operation, industrial internet applications, and digital cultural and creative industries—to ensure that skill supply aligns with future industrial demands and facilitates the realization of digital skill value.

Thirdly, utilize the radiation effect of urban agglomerations to construct cross-regional hubs for digital talent development and resource sharing. Fully capitalize on the advantages of major urban agglomerations—such as the Beijing-Tianjin-Hebei region, the Yangtze River Delta, and the Pearl River Delta—as digital resource hubs. Support core cities in these urban agglomerations to establish "Digital Talent Training and Exchange Centers" and develop high-quality, standardized training courses and online learning platforms. Through a "pairing assistance" mechanism, encourage these centers to export course resources, teaching personnel, and training opportunities to non-agglomeration areas in surrounding regions. Simultaneously, promote the mutual recognition of digital skills certifications and facilitate talent mobility within and among urban agglomerations, ensuring that returns on digital skills are not confined to local areas but can be realized through broader job markets, thereby amplifying the impact of improving digital literacy.

Thank you again for your valuable suggestions. These revisions have significantly enhanced the policy relevance of our research. We hope the modified content meets your requirements.

Comment 19: The limitations section omits measurement bias issues and should explicitly acknowledge them.

Response: We sincerely appreciate your review of our manuscript and the provision of valuable comments. Besides the original limitations—limitations in data sources for deep skill measurement and assumptions regarding instrumental variables—we have explicitly incorporated and expounded upon the potential measurement biases in digital literacy and employment quality, especially those stemming from self-reporting and social desirability. Moreover, we have discussed the possible influence of these biases on the interpretation of results and outlined directions for future improvement. The revised content acknowledges the measurement challenges while ensuring logical consistency with the empirical design of this study. The specific revisions are as follows (lines 797-833 in the revised manuscript):

However, this study has certain limitations that warrant further investigation. Firstly, the measurement of digital literacy, constrained by data availability, primarily captures the breadth and frequency of digital tool usage (e.g., online activities) and subjective awareness, rather than directly assessing granular cognitive or technical skills (e.g., coding, data analysis, cybersecurity practices, or digital content creation). While we have conceptually justified these behavioral indicators as foundational proxies that correlate with and enable skill application, they cannot fully represent the depth or sophistication of an individual's digital competencies. This reliance on self-reported usage frequency and perceived importance introduces potential measurement biases. Future research would benefit from employing specialized assessments or surveys incorporating items aligned with detailed digital literacy frameworks to more precisely measure specific skill dimensions and their differential impacts on various facets of employment quality.

Secondly, similar measurement considerations apply to our dependent variable. The multi-dimensional index of employment quality, while an advancement, is also based on self-reported data for key components like income, satisfaction, and promotion prospects. Self-reported income may be subject to recall error or rounding, and subjective satisfaction measures can be influenced by transient affective states or individual response tendencies. The potential for common method variance, given that both core variables are derived from the same survey respondent, cannot be entirely ruled out, although the use of composite indices and the inclusion of objective indicators (e.g., contract status, insurance) mitigate this concern to some extent. Future studies could strengthen measurement by linking survey data with administrative records (e.g., social security data for income and contract details) or employing multi-source evaluations.

Thirdly, the instrumental variable method adopted in this paper relies on the assumption of exclusion restriction, which cannot be directly verified. Despite our comprehensive efforts to substantiate its validity from theoretical and empirical perspectives, this remains a potential limitation of the study. Future research that devises natural experiments or identifies more exogenous instrumental variables---such as early-stage disparities in regional digital infrastructure construction---will be able to provide more robust causal evidence.

Thank you for highlighting this critical point, which enables us to delineate the boundaries and limitations of this research more comprehensively and precisely. We trust that the revised content will fulfill your requirements.

Comment 20: The data availability and ethics statements are incomplete and should provide proper CFPS access details and clarification.

Response: Thank you for your valuable feedback. You pointed out that "the data availability and ethical statement are incomplete, and the correct CFPS access details should be provided and clarified." We wholeheartedly concur and have made the necessary additions and corrections to guarantee the transparency and compliance of the research. In line with your suggestion, we have appended a clear statement regarding the data acquisition process, usage permission, and ethical review at the end of the "Data Source" subsection in "Methodology and Data". The specific modifications are as follows:

Data Source (lines 312-338 in the revised manuscript)

This study utilizes data from the China Family Panel Studies (CFPS), administered by the Institute of Social Science Survey (ISSS) at Peking University. The CFPS adopts a multi-stage, multi-level random probability sampling approach across the country. The dataset encompasses various domains, including economics, sociology, demography, and education, and is characterized by strong sample representativeness. Given the specific selection of variables relevant to this research, the empirical analysis draws on data collected in 2022. The study focuses on individuals within the working-age population, specifically those aged between 18 and 60. After selecting the relevant indicators and excluding responses that were missing, abnormal, or indicated “do not know,” a final sample of 7,625 valid observations was obtained.

It is noteworthy that the CFPS data are publicly available for academic research upon application through its official website (http://www.isss.pku.edu.cn/cfps/). The authors accessed the 2022 wave of data under the terms of the CFPS data use agreement, which mandates compliance with confidentiality and usage regulations. The original CFPS survey protocol received ethical approval from the Biomedical Ethics Review Committee of Peking University. As this study involves secondary analysis of anonymized, publicly available data, it did not require additional institutional review board approval. All procedures performed in the original studies involving human participants were in accordance with the ethical standards of the institutional and national research committees and with the 1964 Helsinki Declaration and its later amendments or comparable ethical standards.

These additions guarantee the transparency of data sources, acquisition methods, and ethical compliance, fully adhering to academic publication standards. Additionally, we have uploaded and submitted an additional ethical review certificate to the journal to meet your requirements. Once again, I express my sincere gratitude for your valuable suggestions, which have contributed to enhancing the rigor of our manuscript.

Attachment Submitted filename: Response to Reviewers.docx

# Peer review: Decision Letter 1

**DOI:** 10.1371/journal.pone.0358194.r003

**Authors:** Md. Rabiul Awal

11 Jun 2026

Dear Dr. Wu,

Thank you for submitting your manuscript to PLOS ONE. After careful consideration, we feel that it has merit but does not fully meet PLOS ONE’s publication criteria as it currently stands. Therefore, we invite you to submit a revised version of the manuscript that addresses the points raised during the review process.

Please submit your revised manuscript by Jul 26 2026 11:59PM. If you will need more time than this to complete your revisions, please reply to this message or contact the journal office at plosone@plos.org. When you're ready to submit your revision, log on to https://www.editorialmanager.com/pone/ and select the 'Submissions Needing Revision' folder to locate your manuscript file.

- A letter that responds to each point raised by the academic editor and reviewer(s). You should upload this letter as a separate file labeled 'Response to Reviewers'.
- A marked-up copy of your manuscript that highlights changes made to the original version. You should upload this as a separate file labeled 'Revised Manuscript with Track Changes'.
- An unmarked version of your revised paper without tracked changes. You should upload this as a separate file labeled 'Manuscript'.

If you would like to make changes to your financial disclosure, please include your updated statement in your cover letter. Guidelines for resubmitting your figure files are available below the reviewer comments at the end of this letter.

If applicable, we recommend that you deposit your laboratory protocols in protocols.io to enhance the reproducibility of your results. Protocols.io assigns your protocol its own identifier (DOI) so that it can be cited independently in the future. For instructions see: https://journals.plos.org/plosone/s/submission-guidelines#loc-laboratory-protocols. Additionally, PLOS ONE offers an option for publishing peer-reviewed Lab Protocol articles, which describe protocols hosted on protocols.io. Read more information on sharing protocols at https://plos.org/protocols?utm_medium=editorial-email&utm_source=authorletters&utm_campaign=protocols.

As the corresponding author, your ORCID iD is verified in the submission system and will appear in the published article. PLOS supports the use of ORCID, and we encourage all coauthors to register for an ORCID iD and use it as well. Please encourage your coauthors to verify their ORCID iD within the submission system before final acceptance, as unverified ORCID iDs will not appear in the published article. *Only*  the individual author can complete the verification step; PLOS staff *cannot*  verify ORCID iDs on behalf of authors.

We look forward to receiving your revised manuscript.

Kind regards,

Md. Rabiul Awal

Bangladesh Army University of Science and Technology

Academic Editor

PLOS One

Journal Requirements:

If the reviewer comments include a recommendation to cite specific previously published works, please review and evaluate these publications to determine whether they are relevant and should be cited. There is no requirement to cite these works unless the editor has indicated otherwise.

Please review your reference list to ensure that it is complete and correct. If you have cited papers that have been retracted, please include the rationale for doing so in the manuscript text, or remove these references and replace them with relevant current references. Any changes to the reference list should be mentioned in the rebuttal letter that accompanies your revised manuscript. If you need to cite a retracted article, indicate the article’s retracted status in the References list and also include a citation and full reference for the retraction notice.

Additional Editor Comments:

I kindly request that the authors have the entire manuscript carefully reviewed and edited by a native English speaker to address language issues, grammatical errors, and overly technical jargon.

[Note: HTML markup is below. Please do not edit.]

Reviewers' comments:

Reviewer's Responses to Questions

**Comments to the Author**

Reviewer #1: (No Response)

Reviewer #3: All comments have been addressed

**********

2. Is the manuscript technically sound, and do the data support the conclusions?

Reviewer #1: Yes

Reviewer #3: Yes

**********

3. Has the statistical analysis been performed appropriately and rigorously?

Reviewer #1: Yes

Reviewer #3: Yes

**********

4. Have the authors made all data underlying the findings in their manuscript fully available?

The PLOS Data policy

Reviewer #1: Yes

Reviewer #3: Yes

**********

5. Is the manuscript presented in an intelligible fashion and written in standard English?

Reviewer #1: Yes

Reviewer #3: No

**********

Reviewer #1: Thank you very much for the revised version of the paper and for addressing all of my comments. The paper now makes a much better reading than the previous version.

However there are some minor issues which have appeared in the revised version and which I describe in my comments below. Therefore I would recommend a minor revision and an acceptance of the paper for publication after those issues have been addressed.

Minor comments:

1. Something is wrong with the mathematical symbols in Equation (1) – I see only empty boxes in that equation.

2. A similar comment concerns line 452-457: empty boxes instead of mathematical symbols.

3. Line 467-468: as the author/authors uses/use OLS as their research method so what they investigate is the relation between independent variables and the dependent variable and not causality. Therefore, in my view, the statement “denotes a set of control variables that may influence the employment quality of individual labor force” is way too strong and should be rewritten so as to indicate that the author/authors investigates/investigate a relation between the independent variables and dependent variable.

Reviewer #3: The authors have responded constructively to many reviewer concerns and the manuscript addresses an important topic. Nevertheless, concerns remain regarding causal interpretation, transparency of index construction, instrumental variable validity, and ethical reporting. I recommend Minor Revision to ensure methodological rigor and compliance with PLOS ONE reporting standards. Some additional issues need to address:

1. Causal Interpretation Remains Problematic

Although the authors have revised some language from "influence" to "association," the manuscript still occasionally implies causal relationships, particularly in the theoretical framework, discussion, and policy recommendations.

The study relies primarily on cross-sectional observational data from CFPS 2022 and OLS regressions. Such a design does not permit strong causal inference. Even with the instrumental variable approach, the manuscript should more thoroughly justify instrument validity and acknowledge remaining limitations.

Recommendation:

Consistently use associative language throughout the manuscript.

Explicitly state in the limitations section that causality cannot be established.

Temper policy recommendations that assume causal effects.

2. Instrumental Variable Strategy Requires Stronger Justification

The response notes that Models (6) and (7) correspond to first- and second-stage regressions. However, it remains unclear whether the proposed instrument satisfies:

Relevance condition

Exclusion restriction

The manuscript should provide a stronger theoretical justification for why the instrument affects employment quality only through digital literacy.

Recommendation:

Discuss instrument validity in greater detail.

Report relevant diagnostics (e.g., weak instrument tests, first-stage F-statistic, over-identification tests if applicable).

Include a dedicated subsection discussing IV assumptions.

3. Construction of Composite Indices Needs More Transparency

Both digital literacy and employment quality are constructed using entropy-weighted indices. While this approach is reasonable, readers need additional information regarding:

Indicator selection rationale

Weighting procedures

Robustness to alternative weighting schemes

Reliability and validity assessments

Recommendation:

Supplementary tables showing indicator weights, or

Sensitivity analyses using alternative index construction methods.

4. Moderation Analysis Requires Further Interpretation

The moderation results are interesting but remain largely descriptive.

For example:

Why does human capital negatively moderate the relationship?

Could this result reflect ceiling effects?

Are there multicollinearity concerns between education and digital literacy?

Recommendation:

Provide deeper theoretical interpretation and discuss alternative explanations.

**********

what does this mean?). If published, this will include your full peer review and any attached files.

If you choose “no”, your identity will remain anonymous but your review may still be made public.

**Do you want your identity to be public for this peer review?**  For information about this choice, including consent withdrawal, please see our Privacy Policy

Reviewer #1: No

Reviewer #3: No

**********

[NOTE: If reviewer comments were submitted as an attachment file, they will be attached to this email and accessible via the submission site. Please log into your account, locate the manuscript record, and check for the action link "View Attachments". If this link does not appear, there are no attachment files.]

To ensure your figures meet our technical requirements, please review our figure guidelines: https://journals.plos.org/plosone/s/figures

You may also use PLOS’s free figure tool, NAAS, to help you prepare publication quality figures: https://journals.plos.org/plosone/s/figures#loc-tools-for-figure-preparation.

NAAS will assess whether your figures meet our technical requirements by comparing each figure against our figure specifications.

# Author comment: Author response to Decision Letter 2

**DOI:** 10.1371/journal.pone.0358194.r004

24 Jun 2026

Response to Reviewers

Manuscript ID: [PONE-D-25-44300R1]

Title: The Relationship Between Digital Literacy and Employment Quality: Evidence from a Chinese Household Survey

Dear Editors and Reviewers,

We express our sincere gratitude to the editor and reviewers for dedicating their valuable time and providing insightful comments on this manuscript. Following the second round of external review, we believe these detailed suggestions will significantly enhance the quality and clarity of our paper. We have carefully considered all feedback and implemented corresponding revisions to the manuscript. Specific responses to each comment are provided below. In the “revised manuscript with tracked changes,” all modifications have been highlighted in red for your convenience.

Response to Editor Comments

Requirement 1: I kindly request that the authors have the entire manuscript carefully reviewed and edited by a native English speaker to address language issues, grammatical errors, and overly technical jargon.

Response: Thank you very much for your careful and constructive feedback on our manuscript (PONE-D-25-44300R1). We appreciate the time and effort you and the reviewers have invested in evaluating our work. We also sincerely apologize for the language issues and the use of overly technical jargon that may have hindered the clarity and readability of our paper.

In response to your request that the entire manuscript be carefully reviewed and edited by a native English speaker, we have taken the following measures:

1. Professional Language Editing – We engaged a native English speaking editor with extensive experience in social science and economics manuscripts to perform a comprehensive language review. The editor scrutinized every section—from the abstract and introduction to the methodology, results, discussion, and conclusions—correcting grammatical errors, improving sentence structure, and ensuring consistent and precise use of terminology.

2. Reduction of Jargon – In addition to grammatical corrections, we simplified and clarified technical expressions that might be overly specialized. For example:

In the Methodology section, we replaced phrases such as “entropy method” with “entropy weighting method” and provided a plain language explanation of the calculation steps to make the procedure more accessible.

In the Theoretical Analysis section, we rephrased complex theoretical terms (e.g., “diminishing marginal benefit of digital literacy”) with more straightforward expressions while retaining academic rigor.

The Discussion and Conclusions have been rewritten to avoid unnecessary acronyms and to clearly state policy implications in plain English, ensuring that readers from diverse backgrounds can easily grasp the practical takeaways.

3. Consistency and Flow – The editor also checked for coherence across paragraphs, improved transition sentences, and harmonized the use of key terms (e.g., “digital literacy,” “employment quality”) throughout the manuscript to avoid ambiguity.

All changes are marked in the revised manuscript using the “Track Changes” function. We believe that the revised text now meets the high linguistic standards expected by PLOS ONE and that the content is both scientifically precise and broadly understandable.

We are grateful for your guidance and hope that the revised version is now suitable for publication. Should you or the reviewers have any further concerns, we are happy to address them promptly.

Response to Reviewer 1

Minor Comment 1: Something is wrong with the mathematical symbols in Equation (1) – I see only empty boxes in that equation.

Response: Thank you for your careful reading and for bringing to our attention the display issue with the mathematical symbols in Equation (1) of our manuscript. We sincerely apologize for the inconvenience this has caused.

To address this issue, we have thoroughly rewritten Equation (1) in the revised manuscript using a more robust and widely compatible format, and we have also submitted a PDF version of the manuscript. For your convenience, the corrected equation is shown below as an image:

We have verified that the revised equation displays properly in the PDF generated from the updated manuscript. We have also checked the other equations (2)–(7) to ensure they are free of similar issues.

We are grateful for your vigilance, and we hope that this revision fully addresses your concern. Should you encounter any further technical difficulties, please do not hesitate to let us know, and we will be happy to provide alternative formats if needed. Thank you again for your valuable guidance.

Minor Comment 2: A similar comment concerns line 452-457: empty boxes instead of mathematical symbols.

Response: We sincerely thank you for your careful review and for pointing out the display issue with mathematical symbols in lines 452–457 of our original manuscript. We apologize for the inconvenience caused by these rendering problems.

To ensure complete clarity and correct display, we have thoroughly revised all equations in the manuscript. Specifically:

In Equations (1) - (4), Xij denotes the dimensionless normalized value of the jth evaluation index for the ith research unit; n represents the number of research units, and m represents the number of indicators; dj is the entropy value of the jth indicator; Wj is the weight of the jth indicator; Si is the comprehensive evaluation index of the ith research unit.

We have now verified that the revised manuscript, when compiled into PDF, displays all mathematical symbols correctly—with no empty boxes or missing characters. The updated equations are clearly readable and consistent with standard academic formatting.

We are grateful for your vigilance; this correction has improved the technical quality of our paper. Should you have any further concerns, we would be happy to provide a separate plain text version of the equations or supplementary files as needed. Thank you again for your valuable time and suggestions.

Minor Comment 3: Line 467-468: as the author/authors uses/use OLS as their research method so what they investigate is the relation between independent variables and the dependent variable and not causality. Therefore, in my view, the statement “denotes a set of control variables that may influence the employment quality of individual labor force” is way too strong and should be rewritten so as to indicate that the author/authors investigates/investigate a relation between the independent variables and dependent variable.

Response: We are very grateful for your careful and precise reading of our manuscript. Your observation regarding the use of causal language in the model specification section is both insightful and methodologically important. We fully agree that ordinary least squares (OLS) regression, as a correlational method, does not by itself establish causality, and our wording should accurately reflect the nature of our empirical strategy.

In the original text (lines 467–468), we wrote: “Ci denotes a set of control variables that may influence the employment quality of individual labor force i.”

As you rightly pointed out, this phrasing is too strong and implies a causal claim. In the revised manuscript, we have rewritten this sentence to clearly indicate that we are examining associations or relationships, not causal effects. The revised wording now reads (lines 485-486 in the revised manuscript):

“Ci denotes a set of control variables that are associated with the employment quality of individual labor force i.”

We have made this change consistently throughout the manuscript wherever similar causal language appeared, including in the abstract, the theoretical hypothesis statements (which we have rephrased as “is positively associated with” rather than “promotes” or “enhances”), and in the discussion section where we now use terms such as “correlate,” “relationship,” and “association” instead of “impact” or “effect” when referring to the OLS results.

We believe that this revision aligns the language of our paper with the methodological rigor of our analytical approach, and we thank you for helping us improve the precision and clarity of our manuscript.

Response to Reviewer 2

Comment 1: Causal Interpretation Remains Problematic.

Although the authors have revised some language from "influence" to "association," the manuscript still occasionally implies causal relationships, particularly in the theoretical framework, discussion, and policy recommendations. The study relies primarily on cross-sectional observational data from CFPS 2022 and OLS regressions. Such a design does not permit strong causal inference. Even with the instrumental variable approach, the manuscript should more thoroughly justify instrument validity and acknowledge remaining limitations.

Recommendation:

Consistently use associative language throughout the manuscript.

Explicitly state in the limitations section that causality cannot be established.

Temper policy recommendations that assume causal effects.

Response: We sincerely thank you for your careful and rigorous evaluation of our revised manuscript. Your observation regarding the remaining causal language is both constructive and methodologically critical. We fully agree that our cross sectional OLS design, even with instrumental variable (IV) estimation, does not support strong causal claims, and that the manuscript should consistently reflect this limitation.

In response, we have undertaken a thorough revision of the entire manuscript to systematically replace causal language with associative terminology. Below we detail the specific actions taken, addressing each of your recommendations.

1. Consistent use of associative language throughout the manuscript.

We have carefully reviewed and revised every section to ensure that language reflects correlation or association rather than causation. Key modifications include:

Title:

Original: “Research on the Impact and Mechanism of Digital Literacy on Employment Quality: Evidence from the China Family Panel Studies”

Revised: “The Relationship Between Digital Literacy and Employment Quality: Evidence from a Chinese Household Survey”

Abstract: Changed “determinant” to “correlate”; replaced “promoting” with “associated with”; revised “enhance” to “correspond to”.

Introduction and Theoretical Framework: All hypotheses have been reworded. For example:

Original: “Digital literacy has a significant positive impact on labor employment quality.”

Revised: “Digital literacy is significantly positively associated with labor employment quality.”

Similarly, H2 and H3 now state “positively moderates the association” and “negatively moderates the association,” rather than “impact.”

Methodology: The model specification now states that we examine the “relationship” or “association” between variables, not their “effect” or “influence.” For instance, the sentence describing Ci (control variables) has been changed from “may influence” to “are associated with” (as previously adjusted per your earlier suggestion).

Results and Discussion: We replaced all instances of “effect” (when referring to OLS coefficients) with “association” or “coefficient.” For example, “the positive effect” becomes “the positive association”; “the impact of digital literacy” becomes “the relationship between digital literacy and employment quality.”

Conclusions and Policy Implications: We have significantly tempered the language. We now frame our policy recommendations as preliminary implications derived from observed associations, rather than established causal effects. We explicitly state that these suggestions should be considered alongside other evidence and are not definitive prescriptions.

All changes are marked in the revised manuscript using track changes. Thank you again for your valuable time and suggestions.

2. Explicitly state in the limitations section that causality cannot be established.

We have expanded the Limitations subsection (formerly in Discussion, now clearly labeled) to explicitly address the causal inference issue. The added text reads (lines 901-911 in the revised manuscript):

Fourthly, our empirical strategy primarily relies on cross sectional observational data from the 2022 CFPS and OLS regressions. While we have employed propensity score matching and instrumental variable estimation to mitigate endogeneity concerns, these methods do not fully eliminate the possibility of unobserved confounding or reverse causality. The IV approach, in particular, rests on the untestable exclusion restriction assumption, which we have justified on theoretical grounds but cannot verify directly. Therefore, we interpret all our findings as associative evidence rather than causal effects. Readers and policymakers should exercise caution when attributing changes in employment quality to improvements in digital literacy based solely on this study.

This addition is placed immediately after the discussion of IV limitations, ensuring that the overall conclusion clearly acknowledges the correlational nature of our evidence.

3. Temper policy recommendations that assume causal effects.

We have revised the policy recommendations section to reflect a more cautious tone. Instead of stating that policies “will” improve employment quality, we now emphasize that our findings suggest or indicate potential directions that merit consideration, and that pilot testing and further evaluation are necessary before large scale implementation.

For example:

Original: “Policies should prioritize … digital skills compensation training programs … to address their traditional skill shortages.”

Revised: “Our findings suggest that policies could prioritize … digital skills compensation training programs … as a means to potentially address traditional skill shortages, though such interventions should be rigorously evaluated.”

Similarly, in the regional strategies, we replaced definitive statements like “policies should expedite” with “policymakers may consider expediting,” and we added a caveat that local contexts and complementary investments are crucial for success.

For your convenience, we hereby present the revised policy recommendations below, with the changes highlighted in red (lines 934-1004 in the revised manuscript):

Given the correlational nature of our empirical evidence, the following policy implications should be interpreted as preliminary and tentative. They are intended to stimulate discussion and inform hypothesis generation for future evaluation, rather than to serve as definitive causal prescriptions. Any actual policy design should be preceded by rigorous pilot testing and contextual feasibility assessments.

Based on these associative findings, we offer the following tentative policy considerations:

Firstly, it may be worthwhile to consider formulating precise and incentive-compatible intervention programs tailored to different human capital groups. Considering that the positive correlation between digital literacy and employment quality is more pronounced among low-human-capital (low educational attainment) workers, policymakers could give priority to and concentrate on this group. It may be advisable to design and implement "digital skills compensation" training programs that closely align basic and advanced digital skills with specific and localized non-agricultural job requirements. Such programs could help address their traditional skill shortages and potentially maximize the utility of digital literacy as a "tool for crossing the employment threshold." Simultaneously, to potentially improve training conversion efficiency, "incentive modules" could be systematically incorporated into the courses. This might involve presenting real-life cases of learners with similar backgrounds who have successfully enhanced their employment quality through improving digital skills, offering clear career development path guidance, and exploring linkages between training certificates and employment recommendations, small-scale entrepreneurship support, and other incentive policies—thus possibly increasing participants' expected returns and stimulating their intrinsic motivation to learn and apply digital skills.

Secondly, policymakers may consider implementing a differentiated strategy to enhance digital literacy and promote industrial development in a coordinated manner. In the western regions, considering that the digital infrastructure is in a catch-up stage and the marginal returns of digital skills are high, two aspects deserve policy attention: First, expediting the popularization of broadband networks and public digital service platforms and lowering the access threshold. Second, vigorously launching the "Basic Digital Skills Popularization Campaign," focusing on training practical skills related to the digital transformation of local characteristic industries, so as to potentially improve the competitiveness of workers in the local labor market. Meanwhile, in the developed eastern regions, the policy focus might transition from "popularization" to "deepening" and "certification." Promoting the establishment of an advanced digital skills certification system, encouraging enterprises, educational institutions, and training organizations to offer relevant courses, and moderately linking them to salary systems and professional title evaluations could help meet the demand for high-level digital talents in industrial upgrading and assist workers in obtaining potential returns. Additionally, considering the characteristics of industrial structure transformation in the central regions, policies could emphasize "demand-driven" approaches. In combination with local manufacturing upgrade plans and service industry development initiatives, tailoring digital skills training content—such as intelligent equipment operation, industrial internet applications, and digital cultural and creative industries—may better align skill supply with future industrial demands and facilitate the realization of digital skill value.

Thirdly, it may be beneficial to utilize the radiation effect of urban agglomerations to construct cross-regional hubs for digital talent development and resource sharing. Fully capitalizing on the advantages of major urban agglomerations—such as the Beijing-Tianjin-Hebei region, the Yangtze River Delta, and the Pearl River Delta—as digital resource hubs, supporting core cities in these urban agglomerations to establish "Digital Talent Training and Exchange Centers" and develop high-quality, standardized training courses and online learning platforms could be considered. Through a "pairing assistance" mechanism, encouraging these centers to export course resources, teaching personnel, and training opportunities to non-agglomeration areas in surrounding regions may help narrow regional gaps. Simultaneously, promoting the mutual recognition of digital skills certifications and facilitating talent mobility within and among urban agglomerations could ensure that returns on digital skills are not confined to local areas but can be realized through broader job markets, thereby potentially amplifying the positive implications of improving digital literacy.

Comment 2: Instrumental Variable Strategy Requires Stronger Justification.

The response notes that Models (6) and (7) correspond to first- and second-stage regressions. However, it remains unclear whether the proposed instrument satisfies:

Relevance condition

Exclusion restriction

The manuscript should provide a stronger theoretical justification for why the instrument affects employment quality only through digital literacy.

Recommendation:

Discuss instrument validity in greater detail.

Report relevant diagnostics (e.g., weak instrument tests, first-stage F-statistic, over-identification tests if applicable).

Include a dedicated subsection discussing IV assumptions.

Response: We sincerely thank you for your rigorous and constructive feedback on our instrumental variable (IV) strategy. You are absolutely correct that the validity of the IV approach hinges critically on both the relevance condition and the exclusion restriction, and that our manuscript needed stronger theoretical justification and more comprehensive diagnostic reporting.

In response to your recommendation, we have undertaken the following revisions:

1. Discuss instrument validity in greater detail.

In the revised manuscript, we have added a dedicated paragraph to provide a more in-depth discussion of the validity of the instrumental variables (lines 601-632 in the revised manuscript). For your convenience, the updated content on the assessment of instrumental variable validity is presented below.

The exclusion restriction requires that the instrument has no direct effect on employment quality other than through digital literacy. We argue that this condition is plausibly satisfied for three interconnected reasons.

First, the perceived value of the Internet is strongly correlated with the endogenous explanatory variable, digital literacy. Individuals who regard the Internet as highly important in their daily routines are more likely to engage actively with digital devices and online platforms, thereby accumulating practical digital skills and knowledge through routine use, self-directed learning, or social interaction. This positive association between perceived importance and actual digital competence has been well-documented in prior literature [4, 12]. Second, the perceived value of the Internet affects the quality of employment solely by influencing digital literacy, and not through any other direct means. An individual's general attitude toward the Internet's importance in daily life is a broad, non specific psychological predisposition, rather than a targeted evaluation of its role in career advancement. Such a general perception is unlikely to directly determine specific labor market outcomes such as wage levels, the existence of a labor contract, social security coverage, or promotion opportunities—these are predominantly shaped by structural market factors, individual professional skills, work experience, and industry and occupation specific characteristics [18]. Third, we emphasize that this variable measures "importance in daily life" rather than specifically "importance in career development," which conceptually differentiates it from direct labor market drivers. While a person who values the Internet highly may also be more motivated to improve digital literacy, that same perception does not, in itself, influence employers' hiring, contracting, or promotion decisions, nor does it affect job characteristics such as industry or occupational rank, except through the digital skills it fosters, thereby satisfying the exclusion restriction required for a valid instrument [20]. Specifically, the instrumental variable should be correlated with the endogenous explanatory variable (digital literacy) and uncorrelated with the error term.

2. Report relevant diagnostics (e.g., weak instrument tests, first-stage F-statistic, over-identification tests if applicable).

We wholeheartedly agree with your perspective. To this end, we have incorporated the F-statistics and Lagrange Multiplier (LM) test statistics for the first stage into Table 6 of the revised manuscript and provided supplementary explanations of the relevant diagnostic results in the “Results and Analysis” section. For your convenience, the newly added diagnostic content in Table 6 and the associated explanatory notes are presented below (lines 633-649 in the revised manuscript).

Table 6. Robustness test and instrumental variable test.

Variable Replace the Explained Variable Change the Sample Size Winsorization Digital Literacy Employment Quality

Model (3) Model (4) Model (5) Model (6) Model (7)

Digital literacy 1.040*** 0.116*** 0.092*** 0.318***

(0.043) (0.012) (0.010) (0.040)

Instrumental variable 0.056***

(0.002)

Control variable Yes Yes Yes Yes Yes

Constant -1.853*** 0.934*** 0.950*** 0.034 0.947***

(0.312) (0.081) (0.074) (0.078) (0.078)

F-value 597.53

LM-value 488.28***

N 7,625 6,862 7,444 7,625 7,625

R2 0.231 0.047 0.037

Note: Model (6) showcases the first-stage regression results of the instrumental variable approach, and Model (7) showcases the corresponding second-stage results.

The IV estimation results are reported in Models (6) and (7) of Table 6. The findings indicate that the instrumental variable is significantly positively correlated with digital literacy. Moreover, the first-stage F-statistic is 597.53, which exceeds the critical value of 16.38 at the 10% significance level, thereby effectively eliminating the presence of weak instrumental variables[35]. The LM statistic is significant at the 1% significance level, indicating that the instrumental variable passes the test for underidentification. After correcting for endogeneity bias, digital literacy continues to exhibit a statistically significant positive association with employment quality in the labor market, and the absolute value of the coefficient increases. This further substantiates the findings of the benchmark regression.

While the exclusion restriction remains empirically untestable, we believe the theoretical reasoning provided above offers a credible basis for its plausibility. We also acknowledge this limitation explicitly in the Discussion section and call for future research using more exogenous instruments.

3. Include a dedicated subsection discussing IV assumptions.

We sincerely thank you for your recommendation to include a dedicated subsection discussing instrumental variable (IV) assumptions. We fully agree that a clearly delineated section focused on IV validity enhances the transparency and methodological rigor of our empirical strategy.

To this end, the “Endogenous Discussion” section is divided into two subsections: Propensity Score Matching (PSM) and Instrumental Variable (IV) Test. The former aims to address potential endogeneity arising from self-selection bias in digital literacy, while the latter rigorously examines the relevance condition and exclusion restriction, and presents detailed diagnostic results (The newly added subsection is located in lines 590 - 649 of the revised manuscript).

We are confident that this dedicated subsection fully addresses your recommendation and substantially enhances the methodological transparency of our manuscript. We are deeply grateful for your careful and constructive guidance.

Comment 3: Construction of Composite Indices Needs More Transparency.

Both digital literacy and employment quality are constructed using entropy-weighted indices. While this approach is reasonable, readers need additional information regarding:

Indicator selection rationale

Weighting procedures

Robustness to alternative weighting schemes

Reliability and validity assessments

Recommendation:

Supplementary tables showing indicator weights, or Sensitivity analyses using alternative index construction methods.

Response: We sincerely thank you for your thoughtful and constructive suggestion to enhance the transparency of our composite index construction. We fully agree that readers need clear information regarding indicator selection rationale, weighting procedures, robustness to alternative schemes, and reliability and validity assessments. In response, we have substantially revised the relevant sections of the manuscript and added supplementary materials to address each of your recommendations.

1. Indicator Selection Rationale.

We have expanded the rationale for indicator selection in both the “Explained Variable” and “Core Explanatory Variable” subsections. The revised text now includes:

For Employment Quality (added text):

“The selection of indicators for employment quality is grounded in the International Labour Organization’s ‘Decent Work’ framework [5], which emphasizes both objective conditions (remuneration, security, and career progression) and subjective perceptions (satisfaction with income, job security, work environment, working hours, and promotion prospects). We include labor remuneration indicators (wage, bonus, and fringe benefits) to capture economic returns; employment security indicators (labor contract, pension insurance, health insurance, and housing provident fund) to reflect job stability and social protection; and career prospects to capture upward mobility. Subjective satisfaction indicators are included to capture workers’ perceived well-being, which is increasingly recognized as a core dimension of employment quality [5]. All indicators are aligned with the multidimensional nature of employment quality and are commonly used in the existing literature [16]. Moreover, to address the limitations associated with relying on single indicators, this study employs the Entropy Method to construct a comprehensive employment quality index. The detailed evaluation framework and the weight of indicators are presented in Table 1 (lines 346-363 in the revised manuscript).”

For Digital Literacy (added text):

“The selection of indicators for digital literacy is guided by established digital literacy frameworks, including UNESCO’s Global Digital Literacy Framework [11] and the European Union’s DigComp framework, which conceptualize digital literacy as a multidimensional construct encompassing both technical skills and cognitive awareness. The ‘digital ability’ dimension captures behavioral engagement with digital tools across four domains—learning, shopping, entertainment, and social activities—which reflect the breadth and diversity of digital tool utilization. Sustained and diverse engagement with digital tools is a fundamental prerequisite for and behavioral manifestation of skill acquisition and development [18]. The ‘digital cognition’ dimension captures subjective perceived importance of the Internet across the same domains, reflecting the motivational and evaluative dimension of digital literacy that influences the depth and purposefulness of technology use. This dual-dimensional approach—combining observed behavioral engagement with subjective cognitive appraisal—provides a more holistic approximation of an individual’s digital literacy than usage alone, acknowledging that in real-world contexts, skill is often exercised and demonstrated through purposeful use [20]. The detailed evaluation indicator system and indicator weights are presented in Table 2.

It is worth noting that the selection of indicators for “digital capabilities”—namely online learning, online shopping, online entertainment, and online social activities—requires conceptual justification. While these items reflect usage frequency or participation rather than directly assessing proficiency levels, they serve as valid proxy measures within the constraints of large-scale social survey data like the CFPS. Grounded in digital literacy theory, sustained and diverse engagement with digital tools is a fundamental prerequisite for and a behavioral manifestation of skill acquisition and development [21, 22]. Furthermore, the inclusion of “digital cognition” (subjective perceived importance across the same domains) complements these behavioral measures by capturing the motivational and evaluative dimension of digital literacy, which influences the depth and purposefulness of technology use. This combined approach of observed behavioral engagement and subjective cognitive appraisal provides a more holistic approximation of an individual’s digital literacy than usage alone, acknowledging that in real-world contexts, skill is often exercised and demonstrated through purposeful use (lines 367-404 in the revised manuscript).”

2. Weighting Procedures.

We have expanded the “Comprehensive Index Construction Method” subsection to provide a detailed step-by-step explanation of the entropy weighting procedure. The revised text now reads (lines 463-475 in the revised manuscript):

Step 1: Dimensional normalization processing. To eliminate scale effects, we normalize each indicator using min – max normalization:

Xij=xij−minxijmaxxij−minxij, if xij is a positive indicatormaxxij−xijmaxxij−minxij, if xij is a negative indicator (1)

Step 2: Calculation of entropy value. For each indicator j, we calculate the entropy value dj as:

dj=i=1nXijn=1nXij×lnXijn=1nXij (2)

Step 3: Determination of indicator weights. The weight Wj for indicator j is calculated as:

Wj=1−dij=1m1−di (3)

Step 4: Calculation of the comprehensive evaluation index. The final composite index Si for each individual i is calculated as:

Si=j=1mWj×Xij (4)

In Equations (1) - (4), Xij denotes the dimensionless normalized value of the jth evaluation index for the ith research unit; n represents the number of research units, and m represents the number of indicators; dj is the entropy value of the jth indicator; Wj is the weight of the jth indicator; Si is the comprehensive evaluation index of the ith research unit.

3. Supplementary tables showing indicator weights.

We sincerely thank you for your thoughtful and constructive suggestion to enhance the transparency of our composite index construction. We have supplemented the weights of all indicators in Tables 1 and 2. The supplemented weights are presented below for your review.

Table 1. Employment quality evaluation system.

Dimension Variable Description Variable Assignment Indicator weights

Objective level Labor remuneration Wage income Log-transformed monthly after-tax wage (¥/month) 0.253

Bonus income Log-transformed monthly after-tax bonus (¥/month) 0.228

Fringe benefits income Log-transformed monthly after-tax fringe benefits amount (¥/month) 0.131

Employment security Labor contract 1 = Yes; 0 = No 0.049

Pension insurance 1 = Yes; 0 = No 0.079

Health insurance 1 = Yes; 0 = No 0.078

Housing provident fund 1 = Yes; 0 = No 0.006

Career prospects Career promotion Promoted in the past 12 months: 1 = Yes; 0 = No 0.069

Subjective level Subjective satisfaction evaluation Income satisfaction 1–5 (Very dissatisfied – Very satisfied) 0.032

Job security perception 1–5 (Very dissatisfied – Very satisfied) 0.018

Work environment satisfaction 1–5 (Very dissatisfied – Very satisfied) 0.015

Working hours satisfaction 1–5 (Very dissatisfied – Very satisfied) 0.020

Promotion prospects satisfaction 1–5 (Very dissatisfied – Very satisfied) 0.021

Table 2. Digital literacy evaluation system.

Dimension Variable Description Variable Assignment Indicator weights

Digital ability Online learning activities 1 = Yes; 0 = No 0.218

Online shopping activities 1 = Yes; 0 = No 0.128

Online entertainment activities 1 = Yes; 0 = No 0.364

Online social activities 1 = Yes; 0 = No 0.073

Digital Application Cognition The importance of using the internet for working 1–5 (Very unimportant – Very important) 0.065

The importance of using the internet for learning 1–5 (Very unimportant – Very important) 0.056

The importance of using the internet for entertainment 1–5 (Very unimportant – Very important) 0.041

The importance of using the internet for social activities 1–5 (Very unimportant – Very important) 0.054

4. Robustness to alternative weighting schemes.

We have added a new sensitivity analysis subsection in the “Robustness Test (lines 545-569 in the revised manuscript)” section to test the robustness of our findings to alternative weighting methods. The added text and supplementary table are as follows:

Table 6. Robustness test and instrumental variable test.

Variable Replace the Explained Variable Change the Sample Size Winsorization Digital Literacy Employment Quality

Model (3) Model (4) Model (5) Model (6) Model (7)

Digital literacy 1.040*** 0.116*** 0.092*** 0.318***

(0.043) (0.012) (0.010) (0.040)

Instrumental variable 0.056***

(0.002)

Control variable Yes Yes Yes Yes Yes

Constant -1.853*** 0.934*** 0.950*** 0.034 0.947***

(0.312) (0.081) (0.074) (0.078) (0.078)

F-value 597.53

LM-value 488.28***

N 7,625 6,862 7,444 7,625 7,625

R2 0.231 0.047 0.037

Note: Model (6) showcases the first-stage regression results of the instrumental variable approach, and Model (7) showcases the corresponding second-stage results.

To verify the robustness of the benchmark regression results, three alternative approaches were employed: First, to examine whether the benchmark regression results are sensitive to the choice of weighting method, we re-estimated the composite index of digital literacy and employment quality using factor analysis, where weights were derived from the factor loadings. Subsequently, we re-ran the benchmark regression analysis using these alternative indices (see Table 6, Model (3)). The results show that in Model (3), the coefficient on digital literacy remains positive and statistically significant at the 1% level. These findings demonstrate that the main conclusions of this paper are robust to different weighting schemes and are not driven by the use of entropy weights; Second, Model (4) is derived by adjusting the sample size—specifically, by randomly selecting 90% of the original sample and repeating the regression process; Third, Model (5) performs a regression analysis after removing the extreme values in the top and bottom 1% of the sample data to mitigate the impact of potential outliers. The corresponding results are presented in Models (3) to (5) of Table 6. The findings reveal that the correlation and statistical significance of digital literacy with employment quality remain largely unchanged across all robustness models. This consistency supports the robustness and reliability of the benchmark regression results, thereby confirming the validity of Hypothesis 1.

Comment 4: Moderation Analysis Requires Further Interpretation.

The moderation results are interesting but remain largely descriptive.

For example:

Why does human capital negatively moderate the relationship?

Could this result reflect ceiling effects?

Are there multicollinearity concerns between education and digital literacy?

Recommendation:

Provide deeper theoretical interpretation and discuss alternative explanations.

Response: We sincerely thank you for your insightful and constructive feedback on our moderation analysis. You are absolutely right that the moderation results, while interesting, require deeper theoretical interpretation and consideration of alternative explanations—particularly regarding the negative moderating effect of human capital, the possibility of ceiling effects, and potential multicollinearity concerns. We have carefully considered each of your points and have substantially revised the manuscript accordingly. In response, we have substantially revised the relevant sections of the manuscript and added supplementary materials to address each of your recommendations.

1. Expanded Theoretical Reasoning for Negative Moderation (H3).

We have substantially expanded the theoretical analysis for Hypothesis 3 (negative moderating effect of human capital) in the “Theoretical Analysis and Research Hypothesis” section. The revised text now reads (lines 242-284 in the revised manuscript):

Human capital theory suggests that an individual’s educational attainment, professional skills, and practical experience serve as fundamental pillars for career development and employment quality [26, 27]. Digital literacy is not only a key indicator of human capital accumulation, but its influence on the quality of labour employment is also significantly mediated by the individual’s existing level of human capital [15].

We propose a negative moderating effect of human capital based on three complementary mechanisms. First, diminishing marginal returns: workers with high levels of human capital—such as advanced education and extensive work experience—have already developed strong employability through formal educational investments. Their employment quality is largely determined by these established credentials, leaving relatively less room for digital literacy to contribute additional improvements. In contrast, workers with low human capital face more substantial skill deficits, and the acquisition of digital literacy can serve as a powerful ‘skill compensator,’ generating larger marginal improvements in their employment outcomes.

Second, substitution and opportunity cost effects: highly educated workers may have already acquired a broad set of competencies that partially overlap with or substitute for digital skills (e.g., analytical reasoning, problem solving, and information evaluation). Moreover, they may face higher opportunity costs in terms of time and effort required to acquire new digital skills, potentially reducing their relative motivation to invest in digital literacy enhancement [28]. Workers with lower educational attainment, by contrast, may perceive digital skills as a more novel and valuable addition to their skill set, leading to greater relative gains.

Third, cognitive and behavioral inertia: individuals with high human capital may exhibit over reliance on their existing competencies, leading to lower engagement with and adoption of emerging digital skill sets [28]. This behavioral pattern can attenuate the potential benefits of digital literacy for this group. Conversely, low human capital workers often face disadvantages in the labour market [29], making them more receptive to new skill acquisition as a strategy for improving their employment prospects.

In summary, while digital literacy contributes positively to employment quality overall, the marginal benefit tends to be larger for individuals with lower human capital—a pattern consistent with the ‘inclusive growth’ potential of digital upskilling policies.” Based on the above analysis, this paper formulates Research Hypothesis 3：

Hypothesis 3: Human capital negatively moderates the association of digital literacy on labor employment quality.

2. Discussion of Ceiling Effects.

We have added a dedicated discussion of ceiling effects in the “Discussion” section (lines 791-820 in the revised manuscript):

Secondly, the negative moderating effect of human capital (H3) reveals a phenomenon with significant policy implications. The significant negative interaction term indicates that the positive association between digital literacy and employment quality is more pronounced among workers with lower education levels and weaker traditional human capital. This challenges the simplistic assumption that the returns to digital skills increase linearly with educational attainment [22]. One plausible explanation for the negative moderating effect of human capital is the presence of ceiling effects. For workers with high educational attainment—particularly those holding college degrees or above—their employment quality may already be near the upper bound of the distribution due to their strong human capital endowments. In such cases, the marginal contribution of digital literacy to further employment quality gains is naturally constrained by the limited remaining room for improvement. This interpretation is consistent with the concept of diminishing marginal returns to skill investment, which predicts that additional skill accumulation yields progressively smaller benefits as individuals approach higher competence levels [48]. However, ceiling effects alone cannot fully explain the observed pattern. If the negative moderation were driven solely by ceiling effects, we would expect the relationship between digital literacy and employment quality to be weakest among the highest educated workers but not necessarily stronger among the lowest educated workers. Our results, however, show a particularly pronounced positive association among low human capital workers, suggesting that digital literacy actively compensates for skill deficits rather than merely operating in a context of low baseline employment quality. This ‘skill compensation’ mechanism is an important complement to the ceiling effect explanation and underscores the potential of digital upskilling to reduce employment inequality.

3. Multicollinearity Concerns.

We present the variance inflation factor (VIF) test results for all variables in the moderating model in Table 4. As shown in Table 4, the VIF values for digital literacy (1.310) and human capital (education) (1.450) are both well below the conventional threshold of 10, and the mean VIF across all variables is only 1.240. These results indicate that multicollinearity between education and digital literacy is not severe in our analysis.

Table 4. VIF test results.

Variables Digital literacy Gender Age Expected returns Mean value of VIF

VIF 1.310 1.030 1.530 1.010

Variables Marital status Political status Household registration type Human capital 1.240

VIF 1.260 1.120 1.230 1.450

We are confident that these revisions provide the deeper theoretical interpretation and consideration of alternative explanations you recommended, and we believe they substantially enhance the conceptual depth and methodological rigor of our moderation analysis. We are deeply grateful for your careful and constructive guidance.

Attachment Submitted filename: Response_to_Reviewers_auresp_2.docx

# Peer review: Decision Letter 2

**DOI:** 10.1371/journal.pone.0358194.r005

**Authors:** Md. Rabiul Awal

27 Aug 2026

The Relationship Between Digital Literacy and Employment Quality: Evidence from a Chinese Household Survey

PONE-D-25-44300R2

Dear Dr. Wu

We’re pleased to inform you that your manuscript has been judged scientifically suitable for publication and will be formally accepted for publication once it meets all outstanding technical requirements.

Within one week, you’ll receive an e-mail detailing the required amendments. When these have been addressed, you’ll receive a formal acceptance letter and your manuscript will be scheduled for publication.

An invoice will be generated when your article is formally accepted. Please note, if your institution has a publishing partnership with PLOS and your article meets the relevant criteria, all or part of your publication costs will be covered. Please make sure your user information is up-to-date by logging into Editorial Manager at Editorial Manager® and clicking the ‘Update My Information' link at the top of the page. For questions related to billing, please contact billing support.

If your institution or institutions have a press office, please notify them about your upcoming paper to help maximize its impact. If they’ll be preparing press materials, please inform our press team as soon as possible -- no later than 48 hours after receiving the formal acceptance. Your manuscript will remain under strict press embargo until 2 pm Eastern Time on the date of publication. For more information, please contact onepress@plos.org.

Kind regards,

Md. Rabiul Awal

Academic Editor

PLOS One

Bangladesh Army University of Science and Technology

Additional Editor Comments (optional):

Reviewers' comments:

Reviewer's Responses to Questions

**Comments to the Author**

Reviewer #1: All comments have been addressed

**********

2. Is the manuscript technically sound, and do the data support the conclusions?

Reviewer #1: (No Response)

**********

3. Has the statistical analysis been performed appropriately and rigorously?

Reviewer #1: (No Response)

**********

4. Have the authors made all data underlying the findings in their manuscript fully available?

The PLOS Data policy

Reviewer #1: (No Response)

**********

5. Is the manuscript presented in an intelligible fashion and written in standard English?

Reviewer #1: (No Response)

**********

Reviewer #1: (No Response)

**********

what does this mean?). If published, this will include your full peer review and any attached files.

If you choose “no”, your identity will remain anonymous but your review may still be made public.

**Do you want your identity to be public for this peer review?**  For information about this choice, including consent withdrawal, please see our Privacy Policy

Reviewer #1: No

**********

# Editor report: Acceptance letter

**DOI:** 10.1371/journal.pone.0358194.r006

**Authors:** Md. Rabiul Awal

PONE-D-25-44300R2

PLOS One

Dear Dr. Wu,

I'm pleased to inform you that your manuscript has been deemed suitable for publication in PLOS One. Congratulations! Your manuscript is now being handed over to our production team.

At this stage, our production department will prepare your paper for publication. This includes ensuring the following:

* All references, tables, and figures are properly cited

* All relevant supporting information is included in the manuscript submission,

* There are no issues that prevent the paper from being properly typeset

You will receive further instructions from the production team, including instructions on how to review your proof when it is ready. Please keep in mind that we are working through a large volume of accepted articles, so please give us a few days to review your paper and let you know the next and final steps.

Lastly, if your institution or institutions have a press office, please let them know about your upcoming paper now to help maximize its impact. If they'll be preparing press materials, please inform our press team within the next 48 hours. Your manuscript will remain under strict press embargo until 2 pm Eastern Time on the date of publication. For more information, please contact onepress@plos.org.

You will receive an invoice from PLOS for your publication fee after your manuscript has reached the completed accept phase. If you receive an email requesting payment before acceptance or for any other service, this may be a phishing scheme. Learn how to identify phishing emails and protect your accounts at https://explore.plos.org/phishing.

If we can help with anything else, please email us at customercare@plos.org.

Thank you for submitting your work to PLOS One and supporting open access.

Kind regards,

PLOS One Editorial Office Staff

on behalf of

Dr. Md. Rabiul Awal

Academic Editor

PLOS One
